# Experimental evolution links post-transcriptional regulation to *Leishmania* fitness gain

**DOI:** 10.1371/journal.ppat.1010375

**Published:** 2022-03-16

**Authors:** Laura Piel, K. Shanmugha Rajan, Giovanni Bussotti, Hugo Varet, Rachel Legendre, Caroline Proux, Thibaut Douché, Quentin Giai-Gianetto, Thibault Chaze, Thomas Cokelaer, Barbora Vojtkova, Nadav Gordon-Bar, Tirza Doniger, Smadar Cohen-Chalamish, Praveenkumar Rengaraj, Céline Besse, Anne Boland, Jovana Sadlova, Jean-François Deleuze, Mariette Matondo, Ron Unger, Petr Volf, Shulamit Michaeli, Pascale Pescher, Gerald F. Späth

**Affiliations:** 1 Institut Pasteur, Université de Paris, INSERM U1201, Unité de Parasitologie moléculaire et Signalisation, Paris, France; 2 The Mina and Everard Goodman Faculty of Life Sciences and Advanced Materials and Nanotechnology Institute, Bar-Ilan University, Ramat-Gan, Israel; 3 Institut Pasteur, Bioinformatics and Biostatistics Hub, Department of Computational Biology, USR 3756 IP CNRS, Paris, France; 4 Institut Pasteur, Biomics, Paris, France; Institut Pasteur, UTechS MSBio, Paris, France; 5 Institut Pasteur, Proteomics Platform Mass Spectrometry for Biology UTechS, C2RT, USR2000 CNRS, Paris, France; 6 Department of Parasitology, Faculty of Science, Charles University, Prague, Czech Republic; 7 Université Paris-Saclay, CEA, Centre National de Recherche en Génomique Humaine, Evry, France; National Institute of Health, UNITED STATES

## Abstract

The protozoan parasite *Leishmania donovani* causes fatal human visceral leishmaniasis in absence of treatment. Genome instability has been recognized as a driver in *Leishmania* fitness gain in response to environmental change or chemotherapy. How genome instability generates beneficial phenotypes despite potential deleterious gene dosage effects is unknown. Here we address this important open question applying experimental evolution and integrative systems approaches on parasites adapting to *in vitro* culture. Phenotypic analyses of parasites from early and late stages of culture adaptation revealed an important fitness tradeoff, with selection for accelerated growth in promastigote culture (fitness gain) impairing infectivity (fitness costs). Comparative genomics, transcriptomics and proteomics analyses revealed a complex regulatory network associated with parasite fitness gain, with genome instability causing highly reproducible, gene dosage-independent and -dependent changes. Reduction of flagellar transcripts and increase in coding and non-coding RNAs implicated in ribosomal biogenesis and protein translation were not correlated to dosage changes of the corresponding genes, revealing a gene dosage-independent, post-transcriptional mechanism of regulation. In contrast, abundance of gene products implicated in post-transcriptional regulation itself correlated to corresponding gene dosage changes. Thus, RNA abundance during parasite adaptation is controled by direct and indirect gene dosage changes. We correlated differential expression of small nucleolar RNAs (snoRNAs) with changes in rRNA modification, providing first evidence that *Leishmania* fitness gain in culture may be controlled by post-transcriptional and epitranscriptomic regulation. Our findings propose a novel model for *Leishmania* fitness gain in culture, where differential regulation of mRNA stability and the generation of modified ribosomes may potentially filter deleterious from beneficial gene dosage effects and provide proteomic robustness to genetically heterogenous, adapting parasite populations. This model challenges the current, genome-centric approach to *Leishmania* epidemiology and identifies the *Leishmania* transcriptome and non-coding small RNome as potential novel sources for the discovery of biomarkers that may be associated with parasite phenotypic adaptation in clinical settings.

## Author summary

Genome instability plays a central yet poorly understood role in human disease. Gene amplifications and deletions drive cancer development, microbial infection and therapeutic failure. The molecular mechanisms that harness the deleterious effects of genome instability to generate beneficial phenotypes in pathogenic systems are unknown. Here we study this important open question in the protozoan parasite *Leishmania* that causes devastating human diseases termed leishmaniases. *Leishmania* parasites lack transcriptional control and instead exploit genome instability to adapt to their host environment. Analyzing *in vitro* adaptation of hamster-derived parasites *via* gene copy number (genomic level) and gene expression changes (transcriptomic and proteomic levels), we show that these parasites likely exploit small nucleolar RNAs (snoRNAs) to mitigate toxic effects of genome instability by post-transcriptional regulation and the establishment of modified ribosomes. Our findings propose non-coding RNAs as potential novel biomarkers with diagnostic and prognostic value that may be linked to changes in parasite tissue tropism or drug susceptibility. This novel insight into *Leishmania* adaptation will be likely applicable to other fast evolving eukaryotic systems with unstable genomes, such as fungi or cancer cells.

## Introduction

Parasitic protozoa of the genus *Leishmania* are the etiologic agents of a spectrum of severe diseases known as leishmaniases that cause substantial human morbidity and are among the five most serious parasitic diseases worldwide. Today, almost 1 billion people are at risk of *Leishmania* infection in close to 100 endemic countries throughout tropical and subtropical regions, with over 12 million people diagnosed with the infection [1]. Leishmaniasis represents a global public health challenge: recurrent epidemics are observed in South America, the Maghreb, Middle East, East Africa and India, and *Leishmania* infection has been declared an emerging disease in the EU and South East Asia [1,2]. In absence of treatment, visceral leishmaniasis (VL—also known as Kala Azar) is the most severe and fatal form of the disease, caused either by *Leishmania* (*L*.) *donovani* or *L*. *infantum*.

Most *Leishmania* species show a digenetic life cycle comprising two major developmental stages that infect two distinct hosts. The motile, extracellular promastigote form of *Leishmania* proliferates inside the digestive tract of the sand fly insect vector. After migration towards the stomodeal valve, they eventually differentiate into the infectious metacyclic form, which is transmitted to the mammalian host during the blood meal. Once phagocytosed by host macrophages, metacyclic promastigotes differentiate in the non-motile, intracellular amastigote form that proliferates inside fully acidified, macrophage phagolysosomes of mammalian hosts. Aside stage differentiation, the success of *Leishmania* as a pathogenic microbe relies on its capacity to adapt to a variety of environmental fluctuations encountered in their hosts via an evolutionary process. Evolutionary adaptation relies on the classical Darwinian paradigm, where spontaneous mutations and stochastic changes in gene expression generate genetically and phenotypically heterogenous populations that compete for reproductive success in a given environment, thus driving natural selection of the fittest individuals [3]. While this process is well understood in viral and bacterial infections, only little information is available on evolutionary adaptation of eukaryotic pathogens, notably protozoan parasites. This is especially relevant to trypanosomatids, which—in contrast to classical eukaryotes—do not regulate expression of protein coding genes by transcriptional control. Transcription of protein coding genes in these early-branching eukaryotes is constitutive, with genes being arranged in long, polycistronic transcription units, and mature mRNAs being generated from precursors via a *trans*-splicing process unique to kinetoplastidae [4,5]. In the absence of classical transcriptional regulation [5], *Leishmania* has evolved and emphasized other forms of gene expression control, notably regulation of RNA abundance by post-transcriptional regulation and gene dosage variations [6–10].

A hallmark of *Leishmania* biology is the intrinsic plasticity of its genome, with frequent copy number variations (CNVs) of individual genes or chromosomes linked to drug resistance or changes in tissue tropism [7,11–16]. Combining experimental evolution and comparative genomics approaches, we recently linked both forms of genome instability to fitness gain *in vitro*. DNA read depth analysis of the genomes of *L*. *donovani* parasites adapting to culture identified amplification of a series of chromosomes as highly reproducible drivers of fitness gain [10]. Long-term adaptation in contrast was linked to the positive selection of gene copy number variants, which were amplified as part of functionally related, epistatic networks that allowed the emergence of phenotypes linked to ribosomal biogenesis, translation and proliferation [17]. *Leishmania* genomic adaptation thus occurs through a two-stage process reminiscent to other fast-growing eukaryotic cells (e.g. fungi and cancer cells [18,19]), involving short-term adaptation by karyotypic changes and long-term adaptation through slower gene CNVs [10,17,20].

Together these reports draw a complex picture of *Leishmania* fitness gain in culture and raise a series of important new questions on (i) the nature of the genes that drive positive selection of the observed karyotypic changes during fitness gain *in vitro*, (ii) the potential fitness costs in infectivity associated with karyotypic adaptation, and (iii) the mechanisms evolved by the parasite to harness genome instability for fitness gain in culture and to compensate for deleterious gene dosage effects. Here we combined experimental evolution and integrative systems approaches to address these questions and gain novel insight into regulatory mechanisms underlying *Leishmania* fitness gain during adaptation to culture. Our analyses suggest mechanisms at gene, transcript and protein levels that can harness genome instability for fitness gain *in vitro* through gene dosage-dependent changes that affect post-transcriptional regulation and gene dosage-independent changes in epitranscriptomic control and ribosomal biogenesis.

## Material and Methods

### Ethics statement

Work on animals was performed in compliance with French and European regulations on care and protection of laboratory animals (EC Directive 2010/63, French Law 2013–118, February 6th, 2013). All animal experiments were approved by the Ethics Committee and the Animal welfare body of Institut Pasteur (dha190013 and 180091) and by the Ministère de l’Enseignement Supérieur, de la Recherche et de l’Innovation (project n°#19683).

### Animals

Six to eight-week-old, female mice (*Mus musculus*, C57BL/6JRj) and 5 female Golden Syrian hamsters (*Mesocricetus auratus* RjHan:AURA, weighting between 60–70 g) were purchased from Janvier Laboratories. All animals were handled under specific, pathogen-free conditions in biohazard level 3 animal facilities (A3) accredited by the French Ministry of Agriculture for performing experiments on live rodents (agreement A75-15-01).

### Parasites and culture

*Leishmania donovani* strain 1S2D (MHOM/SD/62/1S-CL2D) was obtained from Henry Murray, Weill Cornell Medical College, New York, USA and maintained by serial passages in hamsters. Amastigotes were recovered from infected hamster spleen and differentiated into promastigotes in M199 complete medium (M199, 10% FBS, 20 mM HEPES; 100 μM adenine, 2 mM L-glutamine, 10 μg/ml folic acid, 13.7 μM hemin, 4.2 mM NaHCO_3_, 1xRPMI1640 vitamins, 8 μM 6-biopterin, 100 units penicillin and 100 μg/ml streptomycin, pH 7.4) at 26°C. Promastigotes, derived from splenic amastigotes, were serially passaged once stationary phase was reached for less than 5 passages (EP, early passage) or 20 passages (LP, late passage) corresponding to ~ 20 and 190 generations, respectively. Luciferase transgenic *Leishmania donovani* strain 1S (EP.luc, kindly provided by T. Lang; [21]) were serially passaged as described above.

### Experimental design

Strains issued from independent experimental evolution assays are identified by number (i.e. EP.1 and LP.1 are the strains resulting from experiment 1, see S9 Fig for details). For comparative analyses DNA, RNA or proteins were extracted from the different EP and LP logarithmic, stationary or metacyclic-enriched parasites as presented in S9 and S10 Figs. Phenotypic characterization was performed on three different cultures prepared from EP.1, LP.1, EP.luc and LP.luc using frozen aliquots as starting material (see S10 Fig).

### Parasite growth and determination of the generation time

Promastigotes in exponential growth phase were seeded at 2x10^6^ (EP) or 1x10^5^ (LP) parasites per ml in M199 complete medium. The different seeding densities allowed to compensate for the difference of EP and LP parasites in growth and to guarantee that they both reach stationary growth phase at the same time, which is essential for the comparative analyses of stationary-phase and metacyclic parasites. Parasites were counted every 24 hours and the generation time was calculated during logarithmic growth phase according to the formula $doubling\;time=\frac{t\times \log(2)}{\log\;C(t)-\log\;C(i)}$. Experiments were performed in triplicates and statistical significance was assessed by t-test.

### Ficoll gradient centrifugation for metacyclic promastigote enrichment

EP and LP promastigote cultures were prepared as described above and maintained at stationary phase culture for 3 days when cell density, acidification and nutrition depletion trigger the differentiation from procyclic to metacyclic promastigotes. Parasites were collected and adjusted to 3x10^8^ cells/ml. Ficoll PM400 (GE Healthcare) was used to prepare a 20% stock solution in PBS and diluted for preparation of 10% and 5% Ficoll solutions. Four ml of 10% Ficoll were overlaid by 4 ml of 5% Ficoll and 4 ml of parasite suspension were layered on top of the Ficoll cushion. Tubes were centrifuged at 1,300 x *g* for 15 min at room temperature without brake. The metacyclic-enriched fractions were recovered at the interface between the 10% and 5% Ficoll layers. Parasites were washed with PBS and adjusted to the final concentration required for a given experiment.

### DNA extraction and sequencing

The different growth kinetics between EP and LP parasites were considered as described above and DNA was prepared from parasites in exponential culture phase. EP.1/LP.1, EP.7/LP.7, and LP.6 promastigotes were centrifuged at 1,600 x *g* for 10 min at room temperature. Approximately 1x10^8^ promastigotes from logarithmic growth phase were re-suspended in 200 μl PBS and genomic DNA was purified using DNeasy Blood and Tissue kit from Qiagen and RNase A according to the manufacturer’s instructions. DNA concentrations were measured in duplicate by fluorescence using a Molecular Device fluorescence plate reader (Quant-IT kits, Thermo Fischer Scientific). The quality of the DNAs was controlled determining the DNA Integrity Number (DIN) analyzing 20 ng of DNA on a TapeStation 4200 (Agilent). One μg genomic DNA was used to prepare a library for whole genome sequencing on an automated platform, using the Illumina “TruSeq DNA PCR-Free Library Preparation Kit”, according to the manufacturer’s instructions. After normalization and quality control, qualified libraries were sequenced on a HiSeqX5 platform from Illumina (Illumina Inc., CA, USA) at the Centre National de Recherche en Génétique Humaine (CEA, Evry, France), generating paired-ended, 150-bp reads. Sequence quality parameters were assessed throughout the sequencing run. Standard bioinformatics analysis of sequencing data was based on the Illumina pipeline to generate a FASTQ file for each sample.

### RNA extraction and sequencing

The different growth kinetics between EP and LP parasites were considered as described above. Total RNA was extracted from (i) EP.1, LP.1, EP.8, LP.8, EP.9 and LP.10 promastigotes at logarithmic culture phases (EP log/LP log), (ii) EP.1 and LP.1 parasites at 3 day-stationary culture phases (EP stat/LP stat), and (iii) metacyclic-enriched EP.1 parasites (EP.1 meta). Promastigotes were centrifuged at 3,000 x *g* for 10 min at room temperature and re-suspended in the lysis buffer supplied with the Qiagen RNeasy Plus kit. The samples were stored at -80°C and RNA extractions were performed according to the manufacturer’s instructions, including a DNase treatment. RNA integrity was controlled using the Agilent Bioanalyzer. DNase-treated RNA extracts were used for library preparation using the TruSeq Stranded mRNA sample preparation kit (Illumina, San Diego, California) according to the manufacturer’s instructions. An initial poly (A+) RNA isolation step (included in the Illumina protocol) was performed on total RNA to remove ribosomal RNA. Fragmentation was performed on the enriched fraction by divalent ions at high temperature. The fragmented RNA samples were randomly primed for reverse transcription followed by second-strand synthesis to create double-stranded cDNA fragments. No end repair step was necessary. An adenine was added to the 3’-end and specific Illumina adapters were ligated. Ligation products were submitted to PCR amplification. The obtained oriented libraries were controlled by Bioanalyzer DNA1000 Chips (Agilent, # 5067–1504) and quantified by spectrofluorimetry (Quant-iT High-Sensitivity DNA Assay Kit, #Q33120, Invitrogen). Sequencing was performed on the Illumina Hiseq2500 platform at the Biomics Center (Institut Pasteur, Paris, France) to generate single-ended, 130-bp reads bearing strand specificity.

For transcriptome-wide mapping of pseudouridine sites (Ψ-seq), total RNA from EP.1 and LP.1 parasites was either untreated or treated with N-cyclohexyl-N-β-(4-methylmorpholinium) (CMC) in bicine buffer (0.17 M CMC in 50 mM bicine, pH 8.3, 4 mM EDTA, 7 M urea) at 37°C for 20 min. Excess CMC was removed by ethanol precipitation. To remove CMC groups attached to G and U, the CMC-treated RNA was subjected to alkaline hydrolysis with Na_2_CO_3_ (50 mM, pH 10.4) at 37°C for 4h, as previously described [22–25]. The reacted RNA was recovered by phenol chloroform extraction, and ethanol precipitation. An adaptor was ligated to the 3’ end of the total RNA (upon fragmentation) before and after CMC treatment, and cDNA was prepared using Affinity Script reverse transcriptase (Agilent). The cDNA was then ligated to an adaptor, PCR amplified, and the samples were sequenced in an Illumina Next Seq machine in paired-end mode, 42-bp reads (20 million reads for each sample).

For the preparation of the small RNome, whole cell extracts were prepared from *L*. *donovani* EP and LP parasites (5x10^9^), that were washed with PBS and resuspended in 20 mM Tris-HCl (pH 7.7), 25 mM KCl, and 10 mM MgCl_2,_ were equilibrated in a nitrogen cavitation bomb (Parr Instruments Co.) with 750 psi N2 for 1h at 4°C, and disrupted by release from the bomb. After nitrogen cavitation, the ribonucleoproteins (RNPs) were extracted with 0.4 M KCl. Ribosomes were removed by centrifugation for 3h at 35,000 rpm in a Beckman 70.1Ti rotor (150,000 x *g*) and the supernatant was defined as post-ribosomal supernatant (PRS). RNA was extracted after treatment with 100 μg/ml of Proteinase K, 1% SDS in the presence of 100 μg/ml DNaseI and was used for library preparation as described previously [23]. The samples were sequenced in an Illumina Next Seq machine in paired- end mode, 42-bp reads (40 million reads for each sample).

### Protein extraction, digestion and LC-MS/MS acquisition

Exponentially growing EP.2, EP.3, EP.4, EP.5 and LP.2, LP.3, LP.4 and LP.5 promastigotes were centrifuged at 1,600 x *g* for 10 min at 4°C and washed three times with cold PBS. Parasite lysates were prepared in 8 M urea, 50 mM Tris, supplemented with a protease inhibitor cocktail (complete from Roche) and a phosphatase inhibitor cocktail (Phos Stop from Roche), 1 ml of lysis buffer per 1.5x10^9^ promastigotes. After 10 min incubation at 4°C followed by sonication for 5 min (sequence of 10s pulse and 20s pause) the lysates were centrifuged 15 min at 14,000 x *g*, 4°C and the supernatant was collected and stored at -80°C until use. Proteins were quantified by RC DC protein assay (Bio-Rad) and a control of the protein pattern of all the extracts was performed by SDS-PAGE and silver staining. All the biological samples were further processed for MS-based proteomics approach, data acquisition, and statistical analyses.

Biological samples were adjusted to 1.3 μg.μl^-1^ in lysis buffer. Disulfide bridges were reduced in 5 mM DTT (Sigma—43815) for 30 min and alkylated in 20 mM iodoacetamide (Sigma—I1149) for 30 min at room temperature in the dark. Protein samples were diluted 10-fold in 50 mM Tris-HCl and digested with Sequencing Grade Modified Trypsin (Promega—V5111) at a Protein:Trypsin ratio 50:1 overnight. Then a second digestion was performed to complete this step. Proteolysis was stopped by adding formic acid (FA, Fluka—94318) at a 1% final concentration. Resulting peptides were desalted using Sep-Pak SPE cartridge (Waters) according to the manufacturer’s instructions. Peptides were concentrated to almost dryness and were resuspended in 2% Acetonitrile (ACN) / 0.1% FA just before LC-MS/MS injection.

All analyses were performed on a Q Exactive Plus Mass Spectrometer (Thermo Fisher Scientific) coupled with a Proxeon EASY-nLC 1000 (Thermo Fisher Scientific). One μg of peptides was injected into a home-made 50 cm C18 column (1.9 μm particles, 100 Å pore size, ReproSil-Pur Basic C18, Dr. Maisch GmbH, Ammerbuch-Entringen, Germany). Column equilibration and peptide loading were performed at 900 bars in buffer A (0.1% FA). Peptides were separated with a multi-step gradient of 2 to 5% buffer B (80% ACN, 0.1% FA) for 5 min, 5 to 22% buffer B for 150 min, 22 to 45% buffer B for 60 min, 45 to 80% buffer B for 10 min at a flow rate of 250 nL/min over 240 min. Column temperature was set to 60°C. MS data were acquired using Xcalibur software using a data-dependent method. MS scans were acquired at a resolution of 70,000 and MS/MS scans (fixed first mass 100 m/z) at a resolution of 17,500. The AGC target and maximum injection time for the survey scans and the MS/MS scans were set to 3E^6^, 20 ms and 1E^6^, 60ms respectively. An automatic selection of the 10 most intense precursor ions was activated (Top 10) with a 45s dynamic exclusion. The isolation window was set to 1.6 m/z and normalized collision energy fixed to 28 for HCD fragmentation. We used an underfill ratio of 1% corresponding to an intensity threshold of 1.7E^5^. Unassigned precursor ion charge states as well as 1, 7, 8 and >8 charged states were rejected and peptide match was disabled.

### Data analyses

*WGS analysis*: Genomic DNA reads were aligned to the *L*. *donovani* Ld1S reference genome (https://www.ncbi.nlm.nih.gov/bioproject/PRJNA396645, GCA_002243465.1) with BWA mem (version 0.7.12) with the flag -M to mark shorter split hits as secondary. Samtools fixmate, sort, and index (version 1.3) were used to process the alignment files and turn them into bam format [26]. Realigner Target Creator and Indel Realigner from the GATK suite were run to homogenize indels [27]. Eventually, PCR and optical duplicates were labeled with Picard Mark Duplicates (version 1.94 (1484)) (https://broadinstitute.github.io/picard/) using the option “VALIDATION_STRINGENCY = LENIENT”. For each read alignment file, Samtools view (version 1.3) and BEDTools genomecov (version 2.25.0) were used to measure the sequencing depth of each nucleotide [28]. Samtools was run with options “-q 50 -F 1028” to discard reads with a low map quality score or potential duplicates, while BEDTools genomecov was run with options “-d -split” to compute the coverage of each nucleotide. The coverage of each nucleotide was divided by the median genomic coverage. This normalization is done to account for library size differences. The chromosome sequencing coverage was used to evaluate aneuploidy between EP.1 and LP.1 samples. Then for each sample and for each chromosome, the median sequencing coverage was computed for contiguous windows of 2,500 bases. As previously published [10], the stably disomic chromosome 36 was used to normalize chromosome read depth and to estimate chromosome polysomy levels in each sample. Gene counts were produced using feature Counts (version 1.4.6-p3 [29]) with these parameters: -s 0 -t gene -g gene_id and were normalized according to the median-ratio method.

*Genome binning*: The reference genome was divided into contiguous windows of a fixed length,and the sequencing coverage of each window was evaluated and compared across different samples. A window length of 300 bases was used for the shown scatter plot assessing genome-wide CNVs. Both the mean sequencing coverage normalized by the median chromosome coverage and the mean read MAPQ value were computed [20].

*RNAseq analysis*: For total RNAseq data, the bioinformatics analysis was performed using the RNA-seq pipeline from Sequana [30]. Reads were cleaned of adapter sequences and low-quality sequences using cutadapt (version 1.11) [31]. Only sequences of at least 25 nucleotides in length were considered for further analysis. STAR (version 2.5.0a) [32], with default parameters, was used for alignment on the reference genome (GCA_002243465.1). Genes were counted using feature Counts (version 1.4.6-p3) [29] from Subreads package (parameters: -t gene -g gene_id -s 1). Count data were analyzed using R version 3.6.1 [33] and the Bioconductor package DESeq2 (version 1.24.0) [34]. The normalization and dispersion estimation were performed with DESeq2 using the default parameters and statistical tests for differential expression were performed applying the independent filtering algorithm. For each pairwise comparison, raw p-values were adjusted for multiple testing according to the Benjamini and Hochberg (BH) procedure [35] and genes with an adjusted p-value lower than 0.01 were considered differentially expressed. The RNAseq data have been deposited in NCBI’s Gene Expression Omnibus [36] and are accessible through GEO Series accession number GSE165615 (https://www.ncbi.nlm.nih.gov/geo/query/acc.cgi?acc=GSE165615).

For small RNome analysis, Ψ-seq and detection of pseudouridylated sites, the 42 bp sequence reads obtained from the Illumina Genome Analyzer were first trimmed of Illumina adapters using the FASTX toolkit (http://hannonlab.cshl.edu/fastx_toolkit), and reads of 15 nucleotides or less were discarded from subsequent analysis. The remaining reads were mapped to the reference genome (GCA_002243465.1) using SMALT (version 0.7.5) (https://www.sanger.ac.uk/tool/smalt-0/) with the default parameters. Only properly paired partners were retained. Each read pair was “virtually” extended to cover the area from the beginning of the first read to the end of its partner. For each base, the number of reads initializing at that location as well as the number of reads covering the position were calculated. A combination of BEDTools (version 2.26.0) Suite (http://bedtools.readthedocs.io/en/latest/) and in-house Perl scripts was used to calculate the Ψ-ratio and Ψ-fc (fold change), as previously described [23,24].

*Proteomics analysis*: Raw data were analyzed using Max Quant software (version 1.5.3.8) [37] using the Andromeda search engine [38]. The MS/MS spectra were searched against the Ld1S database (https://www.ncbi.nlm.nih.gov/bioproject/PRJNA396645, GCA_002243465.1). The settings for the search included (i) trypsin digestion with a maximum of two missed cleavages, (ii) variable modifications for methionine oxidation and N-terminal acetylation, and (iii) fixed modification for cysteine carbamidomethylation. The minimum peptide length was set to 7 amino acids and the false discovery rate (FDR) for peptide and protein identification was set to 0.01. The main search peptide tolerance was set to 4.5 ppm and to 20 ppm for the MS/MS match tolerance. The setting ‘second peptides’ was enabled to identify co-fragmentation events. Quantification was performed using the XIC-based Label-free quantification (LFQ) algorithm with the Fast LFQ mode as previously described [39]. Unique and razor peptides, including modified peptides, with at least two ratio counts were accepted for quantification. The mass spectrometry proteomics data were deposited to the Proteome X change Consortium via the PRIDE partner repository with the dataset identifier PXD020236 [40].

For the differential analyses, proteins categorized as ‘reverse’, ‘contaminant’ and ‘only identified by site’ were discarded from the list of identified proteins. After log2 transformation, LFQ values were normalized by median centering within conditions (*normalizeD* function of the R package *DAPAR* [41]). Remaining proteins without any LFQ value in one of the conditions (either EP or LP) and at least two values in the other condition were considered as exclusively expressed proteins. Missing values across the four biological replicates were imputed using the imp.norm function of the R package norm (norm: Analysis of multivariate normal datasets with missing values. 2013 R package (version 1.0–9.5)). A limma t test was applied to determine proteins with a significant difference in abundance while imposing a minimal fold change of 2 between the conditions to conclude that they are differentially abundant [42,43]. An adaptive Benjamini-Hochberg procedure was applied on the resulting p-values using the function *adjust*.*p* of R package *cp4p* [44] and the robust method described in Pounds et al. [45] to estimate the proportion of true null hypotheses among the set of statistical tests. The proteins associated to an adjusted p-value inferior to a False Discovery Rate (FDR) of 0.01 have been considered as significant and differentially abundant proteins.

*Gene Ontology (GO)-enrichment analyses and gene category assignment*: The Biological Networks Gene Ontology tool (BiNGO) plugin of the Cytoscape software package (version 3.8.2) was used. A Benjamini & Hochberg false discovery rate with a significance level of 0.05 was applied. The lists of *L*. *donovani* GO terms were built in house (see S1 Code). In order to assign the Gene Ontology Identifiers (GO IDs) we combined the GO-derived identifiers with the ones available from the corresponding orthologs in target species: LdBPK, *L*. *infantum*, *L*. *major*, *L*. *mexicana*, *Typanosoma brucei brucei 927* (Tbru) and *Typanosoma cruzi* (Tcru). For each target species we retrieved both the “curated” and “computed” GO IDs from TriTrypDB on the 11/09/2019. OrthoFinder with the DIAMOND search program was applied to establish orthology between the genes in Ld1S and in target species. In “one-to-many” orthology relations we concatenated all the non-redundant GO IDs from all the homologs. The GO IDs were then assigned based on the hierarchy: LdBPK curated > LdBPK GO > *L*. *infantum* curated > *L*. *major* curated > *L*. *mexicana* curated > Tbru curated > Tcru curated > LdBPK computed > *L*. *infantum* computed > *L*. *major* computed > *L*. *mexicana* computed > Tbru computed > Tcru computed. The GO IDs were assigned if not present in any higher rank GO ID data set. The GO IDs of snoRNAs, UsnRNA, SLRNA and 7SL classes defined by homology with *L*. *major* Friedlin genes were manually attributed. Overall, we assigned biological process (BP), molecular function (MF) and cellular component (CC) GO IDs to 5,246, 4,521 and 7,236 Ld1S genes [17] (see S1 Code).

Total frequency represents the percentage of genes associated with a given GO term in the genome compared to the total number of annotated genes. Cluster efficiency represents the percentage of genes for a given GO term in a data set compared to all genes that are annotated with any GO term in the same data set. Enrichment score corresponds to the number of genes for a given GO term in a data set compared to the total number of genes sharing the same GO identifier in the genome. Cluster efficiencies, total frequencies and enrichment scores are shown in S2–S6 Tables in the GO analyses sections.

Genes and proteins were assigned to categories by combining GO analysis and manual inspection for annotations. Genes or proteins annotated for a GO term, a known function or product were considered to estimate the percentage of genes in each category. Gene or protein categories are presented in S2–S6 Tables.

### Northern blot analyses

Total RNA extracted from EP and LP cells (10 μg) were separated on 10% acrylamide denaturing gels, transferred to nitrocellulose membranes and analyzed by autoradiography. RNA probes were prepared by *in vitro* transcription using α-^32^P-UTP [23]. Three independent northern blots were performed.

### Bone marrow-derived macrophages and infection

Bone marrow exudate cells were recovered from tibias and femurs of C57BL/6JRj female mice (Janvier Labs) and macrophages differentiated in DMEM complete medium (DMEM, 15% FBS, 10 mM HEPES, 50 μM 2-mercaptoethanol, 50 units of penicillin and 50 μg/ml of streptomycin) supplemented with 75 ng/ml of recombinant mouse colony stimulating factor-1 (rmCSF-1, ImmunoTools) [46]. A total of 1.5x10^5^ bone marrow-derived macrophages (BMDMs) were plated on glass coverslips in 24-wells plates and incubated overnight at 37°C, 5% CO_2_ prior to *Leishmania* infection.

Promastigotes from stationary culture phase or metacyclic-enriched parasite fractions were pelleted by centrifugation at 3,000 x *g* for 10 min at room temperature and re-suspended in PBS. The concentration was adjusted to 6x10^7^ parasites per ml and 50 μl were added to the BMDM cultures at a multiplicity of infection (MOI) of 20 parasites per 1 macrophage. Plates were centrifuged at 300 x *g* for 5 min at room temperature to allow for a faster sedimentation of the parasites onto the macrophage monolayer. After 2h of contact, coverslips were washed by successive baths in pre-warmed PBS to remove extracellular parasites and transferred into new 24-wells plates containing fresh pre-warmed DMEM culture medium supplemented with 30 ng/μl of rmCSF-1. At 4, 24, 48 and 168h post-infection, cells were fixed in 4% paraformaldehyde (Electron Microscopy Science) and macrophage and parasite nuclei were stained with Hoechst 33342. Images were acquired using a Zeiss Apotome microscope at 40x magnification connected to an Axiocam camera. All the infections were performed in triplicates and at least 100 macrophages were counted per coverslip. The total numbers of infected and non-infected macrophages were recorded and the percentage of infection, the number of parasites per 100 cells and the number of parasites per infected macrophages was calculated and normalized to the values obtained at the initial 4-hour time point. The replication rate in macrophages was calculated between day 1 and day 6 after infection. All the experiments were performed three times in triplicates (see experimental overview in S10 Fig for details) using independent preparations of primary macrophages for each infection.

### Morphological analyses

Parasites were seeded on poly L-lysine treated coverslips and fixed in 2.5% glutaraldehyde. Coverslips were mounted on glass slides using Mowiol 4–88 (Sigma-Aldrich). Images were acquired using an Axiophot microscope at 63x magnification and an Andor camera. Length and width of the parasite cell body, and flagellum length were measured for at least 200 promastigotes using the Image J Fidji software package (https://imagej.net/). The ratios flagellum over body length and body length over body width were determined for the 200 parasites and the Kruskal-Wallis test was used for statistical analysis. The experiment was performed in duplicate.

### Sand fly infection

The colony of *Phlebotomus orientalis* (originating from Ethiopia), the natural vector of *L*. *donovani*, was maintained in the insectary of the Department of Parasitology, Charles University in Prague, under standard conditions (26°C on 50% sucrose and 14h light/10h dark photoperiod) as described previously [47,48].

Promastigotes from logarithmic-phase cultures (day 3–4 in culture) were washed twice in saline solution and re-suspended in heat-inactivated rabbit blood at a concentration of 1x10^6^ promastigotes/ml. Sand fly females (5–9 days old) were infected by feeding through a chick-skin membrane (BIOPHARM) on a promastigote-containing suspension. Engorged sand flies were separated and maintained under the same conditions as the colony. On day 8 post-blood meal (PBM), 150 sand fly females were dissected. The thoracic parts and abdominal parts of infected guts were collected separately and pooled together into two samples: thoracic parts of gut (TP) and abdominal parts of gut (AP). The exact numbers of all parasite stages were calculated using a Burker apparatus and the proportion of metacyclic forms was identified on a Giemsa-stained smears separately for TP and AP. *Leishmania* with flagellar length < 2 times body length were scored as procyclic forms and those with flagellar length ≥2 times body length as metacyclic forms [49].

## Results

### *L*. *donovani* long-term culture adaptation causes a fitness tradeoff between *in vitro* proliferation and infectivity

**Fig 1:**
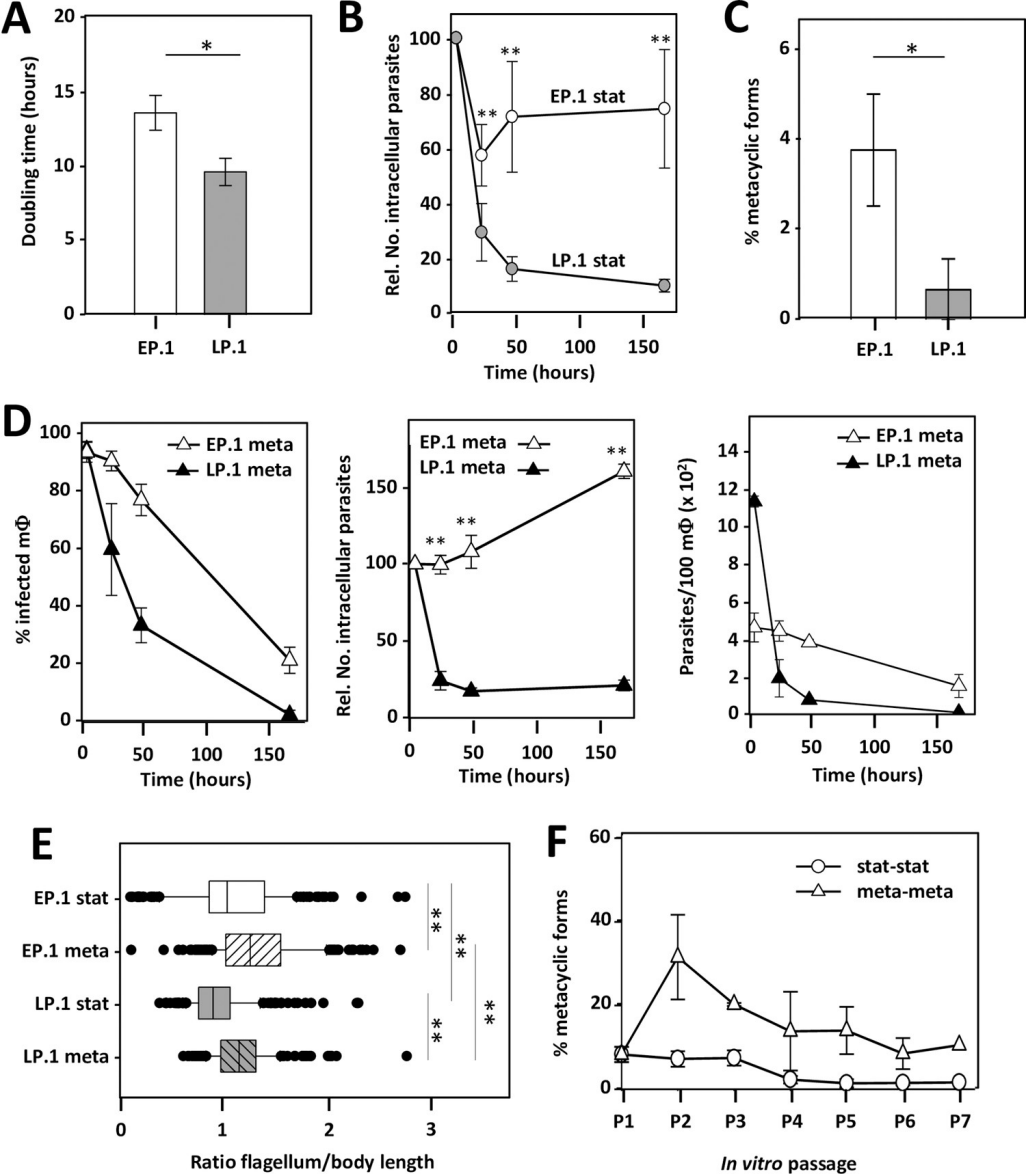
Phenotypic analysis of EP.1 and LP.1 parasites reveals fitness tradeoff between *in vitro* proliferation and macrophage infectivity. (A) Histogram plot representing the generation time of EP.1 and LP.1 promastigotes in culture calculated based on parasite density during logarithmic growth phase. The mean value of three independent experiments +/- SD is represented. *p-value ≤ 0.05. (B) Macrophage infection assay. The mean relative number of intracellular EP.1 (open circles) and LP.1 (grey circles) parasites +/- SD of three independent triplicate experiments using promastigotes from day-3 stationary culture is represented. **p-value ≤ 0.01. (C) Histogram plot representing the percentage of EP.1 and LP.1 metacyclic forms that were enriched by Ficoll density gradient centrifugation from cultures at stationary growth phase. Each bar represents the mean +/- SD of four independent experiments. *p-value ≤ 0.05. (D) Macrophage infection assay using Ficoll-enriched metacyclic parasites. Percentage of infected macrophages (left panel), mean relative number of intracellular EP.1 and LP.1 parasites (middle panel) and mean number of parasites per 100 macrophages (right panel) are shown. Open triangles, EP.1 meta; close triangles, LP.1 meta. The mean values +/- SD of one triplicate experiment are shown. **p-value ≤ 0.01. (E) Morphological characterization of EP.1 and LP.1 Ficoll-enriched metacyclic parasites. Body width, flagellum and body length were measured on 200 promastigotes using the Image J software package. The ratio flagellum-to-body length was computed from two biological replicate experiments and the median values +/- SD are represented by the box plot with the upper and lower quartiles indicated. **p-value ≤ 0.01. (F) Percentage of metacyclic-like parasites recovered by Ficoll gradient centrifugation from cultures seeded successively for 6 *in vitro* passages with either EP.1 from stationary growth phase (stat-stat) or EP.1 metacyclic-enriched parasites (meta-meta). Mean values of two independent experiments are shown with +/-SD denoted by the bars.

In microbial culture, fitness gain (defined as reproductive capacity) largely equals the level of cell proliferation. Adaptation to *in vitro* growth thus represents a simple experimental system to assess mechanisms underlying fitness gain. Here we applied such an experimental evolution approach on *L*. *donovani* amastigotes isolated from infected hamster spleen. Derived promastigotes at early-passage (EP.1) and late-passage (LP.1) were monitored for growth and infectivity with the aim to assess regulatory mechanisms underlying fitness gain and fitness cost observed during culture adaptation. Analyzing cell growth during promastigote culture adaptation revealed robust fitness gain as judged by the reduction in generation time from 13.76 +/- 1.18 hours for EP.1 to 9.76 +/- 0.93 hours for LP.1 promastigotes (Fig 1A). We next evaluated fitness of these parasites in intracellular macrophage infection, where reproductive success depends on parasite resistance to host cell cytolytic activities, amastigote differentiation and proliferation. BMDMs were incubated with EP.1 and LP.1 promastigotes from day-3 stationary culture (referred to as EP.1 stat and LP.1 stat) and intracellular growth was monitored microscopically for 7 days as previously described [50]. Even though the number of EP.1 stat and LP.1 stat intracellular parasites decreased over the first 24h post-infection, only EP.1 parasites recovered and established persistent infection, while the number of LP.1 parasites steadily declined during the subsequent 6 days (Figs 1B and S1A). The same results were obtained in an independent evolutionary experiment conducted with transgenic parasites expressing luciferase, EP.luc and LP.luc (S1C Fig). Together these data firmly establish the highly reproducible nature of the fitness tradeoff between *in vitro* proliferation and infectivity in macrophages as a result from long-term *L*. *donovani* culture adaptation and confirm our previous reports [10,50].

We then tested if the fitness cost of LP.1 stat in infectivity was due to a differentiation defect of infectious metacyclic promastigotes. Considering that stationary phase cultures are composed of different forms of promastigotes, a Ficoll gradient centrifugation method was used to enrich and quantify metacyclic parasites. This method, based on separation of the different parasite forms according to their density [51], allowed to reveal a 5.5-fold reduction in the number of metacyclic parasites from 3.8% in EP.1 stat to 0.69% in LP.1 stat cultures (Fig 1C), the latter one in addition being compromised to establish macrophage infection (Figs 1D and S1B). These results document that the fitness cost in LP.1 meta not only affects the quantity but also the quality of differentiating metacyclic parasites. This was further confirmed by their atypical morphology that was different to *bona fide*, sand fly-isolated metacyclic promastigotes (S2A Fig), corresponding to leptomonad-like forms as judged by flagellum/body-length ratio and body shape [52,53] (Figs 1E and S2A–S2C). Surprisingly, unlike observed when passaging EP.1 stat parasites, metacyclogenesis was maintained in cultures that were passaged using metacyclic-enriched parasites (EP.1 meta) (Fig 1F).

### Transcriptome profiling informs on mechanisms underlying fitness tradeoff

**Fig 2:**
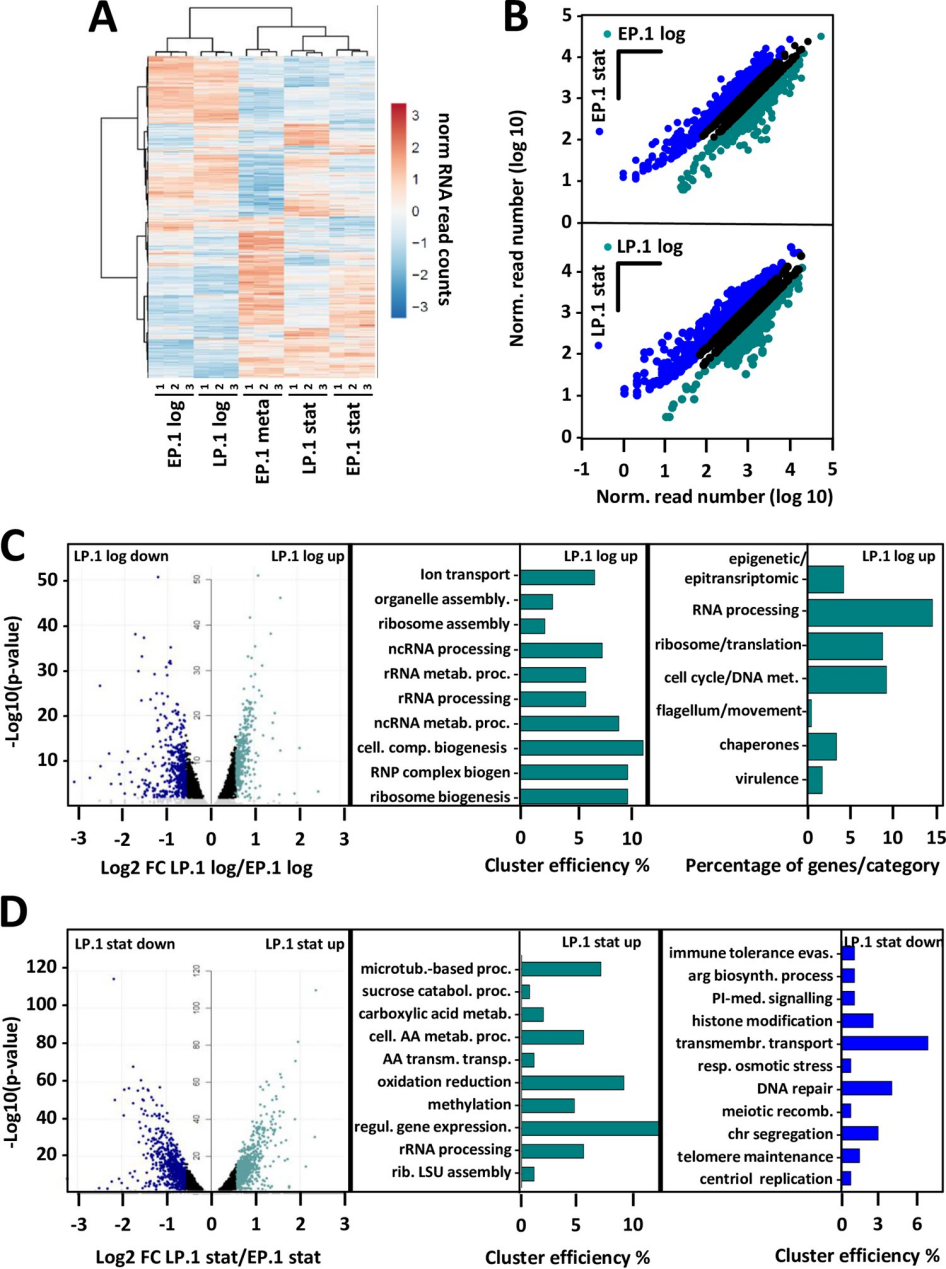
RNA-seq analyses of EP.1 and LP.1 promastigotes reveal stage-specific changes in RNA abundance and RNA signatures linked to fitness gain in culture and fitness cost in infectivity. (A)Cluster analysis of differentially expressed genes observed in triplicate RNAseq analyses of EP.1 log and LP.1 log, EP.1 stat and LP.1 stat, and EP.1 meta parasites. (B) Ratio plots of normalized RNAseq reads for EP.1 log compared to EP.1 stat (upper panel) and LP.1 log compared to LP.1 stat (lower panel). Blue and dark cyan dots represent gene expression changes with FC > 1.5 and adjusted p-value ≤ 0.01; black dots correspond to gene expression changes with adjusted p-value > 0.01. Only genes with at least 10 reads in one of the two conditions were considered. Top panel, 1,499 and 1,501 transcripts more abundant in EP.1 log (dark cyan) and EP.1 stat (blue), respectively. Lower panel, 1,129 and 1,384 transcripts more abundant in LP.1 log (dark cyan) and LP.1 stat (blue), respectively (see Sheets a and d in S2 Table). (C) Differential expression profiling of LP.1 log and EP.1 log parasites. Transcripts more abundant in EP.1 log correspond to transcripts less abundant in LP.1 log. Volcano plot representing the changes in transcript abundances of LP.1 log and EP.1 log parasites with 344 transcripts more abundant in LP.1 log (LP.1 log up) versus 433 transcripts less abundant in LP.1 log (LP.1 log down) (left panel) (see Sheets g and h in S2 Table for the list of regulated genes). Transcripts with significant increased abundance FC > 1.5 and adjusted p-value ≤ 0.01 in LP.1 log up and LP.1 log down are indicated respectively in cyan and blue and were used to perform the GO analysis for the category ‘biological process’. The histogram plot (middle panel) shows ‘cluster efficiency’, which represent the percentage of genes associated with a given GO term compared to the total number of genes with any GO annotation in the considered set of genes. Only functional enrichments associated with adj. p-value < 0.05 were considered. For transcripts more abundant in LP.1 log (LP.1 log up), only 134 out of 344 genes are associated with a GO ID (see Sheet o in S2 Table for details). Transcripts showing increased abundance and adj. p-value <0.01 in LP.1 log were categorized in functional groups (right panel). The histogram plot shows the percentage of genes which represent the number of genes for the indicated gene families compared to the total number of genes with a known function or product (see Sheet I in S2 Table for details). (D) Differential expression profiling of LP.1 stat and EP.1 stat parasites. Transcripts more abundant in EP.1 stat correspond to transcripts less abundant in LP.1 stat. Volcano plot representing the changes in transcript abundances of LP.1 stat and EP.1 stat parasites with 662 transcripts more abundant in LP.1 stat (LP.1 stat up) versus 710 transcripts less abundant in LP.1 stat (LP.1 stat down) (left panel) (see Sheets k and l in S2 Table for the list of up regulated genes). Transcripts with significant increased abundance FC > 1.5 and adjusted p-value ≤ 0.01 in LP.1 stat up and LP.1 stat down are indicated respectively in cyan and blue and were used to perform the GO analysis. Results of GO analyses for the category ‘biological process’ performed on transcripts showing statistically significant increased (middle panel) and decreased (right panel) abundance in LP.1 stat are shown (see Sheet o in S2 Table). Cluster efficiencies were calculated based on 258 and 274 genes with GO IDs in LP.1 stat up and LP.1 stat down set of genes, respectively. Only the functional enrichments associated with adj. p-value < 0.05 were considered.

We performed RNA-seq analyses using poly (A+)-enriched mRNA obtained from three replicates of EP.1 and LP.1 log, stat, and EP.1 meta parasites. The low yield in LP.1 meta parasites precluded their analysis by RNA-seq. Principal component and hierarchical clustering analyses demonstrated that transcript profiles of EP.1 and LP.1 parasites grouped according to stage, indicating that stage-specific expression changes in log, stat and meta parasites dominate over those associated with the EP.1/LP.1 promastigote fitness tradeoff (Sheet a-f in S2 Table and Figs 2A and S3A). Significant stage-specific changes were observed in EP.1 and LP.1 parasites during the log-stat transition for respectively 54.2% and 49.3% of the transcripts and ca. 35% of the promastigote transcriptome was modulated between EP.1 stat and EP.1 meta (Figs 2B and S3B and Sheet a-f in S2 Table). As expected from the increased motility described for metacyclic parasites, we indeed observed increased abundance in EP.1 meta compared to EP.1 log and EP.1 stat for respectively 48 and 51 genes linked to motility and flagellar biogenesis (Sheet p in S2 Table).

We next assessed changes in transcript abundance observed at logarithmic growth phase in LP.1 compared to EP.1 promastigotes to gain first insight into pathways associated with *in vitro* fitness gain (i.e. accelerated growth). We identified 344 transcripts with significantly increased abundance in LP.1 log (Fig 2C, left panel and Sheet g in S2 Table) and revealed functional enrichment in this dataset for the GO terms ‘ribosome biogenesis’, ‘ribosome assembly’, and ‘rRNA processing’ (Fig 2C, middle panel, S3F Fig and Sheet o in S2 Table). Combining GO analysis and manual inspection of gene annotation, 56 genes fell in the categories RNA processing and ribosome/translation, representing 24% of the quantified genes that are annotated for a known function or product (Fig 2C, right panel and Sheet i in S2 Table). LP.1 log fitness gain in culture thus likely reflects an increase in translation efficiency, which may allow for accelerated growth observed in these cells. Further analysis revealed increased abundance of other transcripts implicated in various regulatory processes linked to proliferation (Fig 2C, right panel and Sheet I in S2 Table), including epigenetic/epitranscriptomic regulation (10 genes, e.g. Ld1S_110036500 encoding for a Pseudouridylate synthase 10, Ld1S_260334600 encoding for a RNA pseudouridylate synthase and Ld1S_330597500 encoding for a Histone methyltransferase DOT1) and cell cycle/DNA metabolism (22 genes, e.g. Ld1S_050817000 encoding for CYC2-like cyclin, or Ld1S_330603400 encoding for the cell division control protein CDC45) (see Sheet i in S2 Table for more examples). To assess the reproducibility of these results, we performed RNA-seq analysis on independently evolved LP and EP log parasites (EP.8, EP.9 and LP.8, LP.10) (see PCA in S3D and S9 Figs for details on the origin of these independent biological replicates). Just like in the EP.1/LP.1 comparison, enrichment was observed for various categories linked to ribosomal biology, thus confirming the link between *in vitro* fitness gain and protein translation (S3E Fig).

In contrast, no GO enrichment was observed for the 433 transcripts showing significant reduced abundance in LP.1 log (Sheet h in S2 Table). Manual inspection of gene annotations identified various pathways implicated in metabolism and energy production (e.g. genes encoding for respiratory chain proteins, amino acid and sugar metabolism, fatty acid biosynthesis), signaling (numerous kinases and phosphatases) and flagellum/motility (including four genes encoding for paraflagellar rod components) (S3C Fig and Sheet j in S2 Table). An even stronger reduction of transcripts associated with motility was found in our second transcriptomic analysis of independently evolved LP and EP log parasites (EP.8, EP.9 and LP.8, LP.10, see S9 Fig for details) (S3D and S3E Figs and Sheets b and c in S3 Table). These pathways suggest a potential retooling of the LP.1 log energy metabolism in the nutrition-rich culture environment, and selection against motility, which is not essential in culture and may liberate the energy required for faster growth. Surprisingly, one of the most significant decreases in transcript abundance in these cells was observed for a gene encoding for a 5S ribosomal RNA, along four other genes encoding for ribosomal components (Sheet h in S2 Table), even though other ribosomal components were upregulated in LP.1 log. This result provided a first indication that LP.1 log fitness gain in culture not only depends on the quantity, but likely also the quality or type of ribosomes, e.g. their ribonucleoprotein composition, which may control the fitness-adapted expression profile at the translational level, for example by changing the ribosome translation specificity or efficiency.

Finally, we assessed changes in transcript abundance observed at stationary growth phase in LP.1 compared to EP.1 promastigotes to gain further insight into mechanisms of fitness loss (i.e. attenuated infectivity). We identified 662 transcripts with significantly increased abundance in LP.1 stat (Fig 2D, left panel and Sheet k in S2 Table). Enrichment was observed for the GO terms ‘ribosomal large subunit assembly’, ‘rRNA processing’ and ‘regulation of gene expression’ (Fig 2D, middle panel, S4A Fig and Sheet o in S2 Table). In contrast, LP.1 stat promastigote showed reduced abundance for 710 transcripts, including transcripts linked to the GO terms ‘histone modification’, ‘DNA repair’, ‘transmembrane transport’ (Fig 2D, right panel and Sheet o in S2 Table) and fifteen transcripts manually associated to cell cycle (Sheet n in S2 Table). Likewise, decreased abundance was observed for transcripts associated with the GO term ‘evasion or tolerance of immune response of other organism involved in symbiotic interaction’ and ‘virulence’. Manual inspection allowed us to enrich this last term from originally three to 29 genes (Fig 2D, right panel and S4B Fig and Sheets o and n in S2 Table). Indeed, almost 14% of the transcripts with reduced abundance correspond to genes previously associated with parasites infectivity, including GP63 as well as 31 amastin surface glycoproteins and amastin-like proteins (S4B Fig and Sheet n in S2 Table). Hence, the reduced expression in LP.1 stat parasites of these genes could be associated with attenuated infectivity we observed in LP.1 stat and meta parasites (see Fig 1B and 1D) [54,55].

In conclusion, our data link increased fitness in *in vitro* growth of LP.1 log to a gain-of-function phenotype associated with proliferation, ribosomal biogenesis, and translation. Conversely, the reduced fitness in infectivity of LP.1 stat was associated with a loss-of-function phenotype linked to decreased expression of virulence genes.

### Post-transcriptional adaptation during promastigote fitness gain

**Fig 3:**
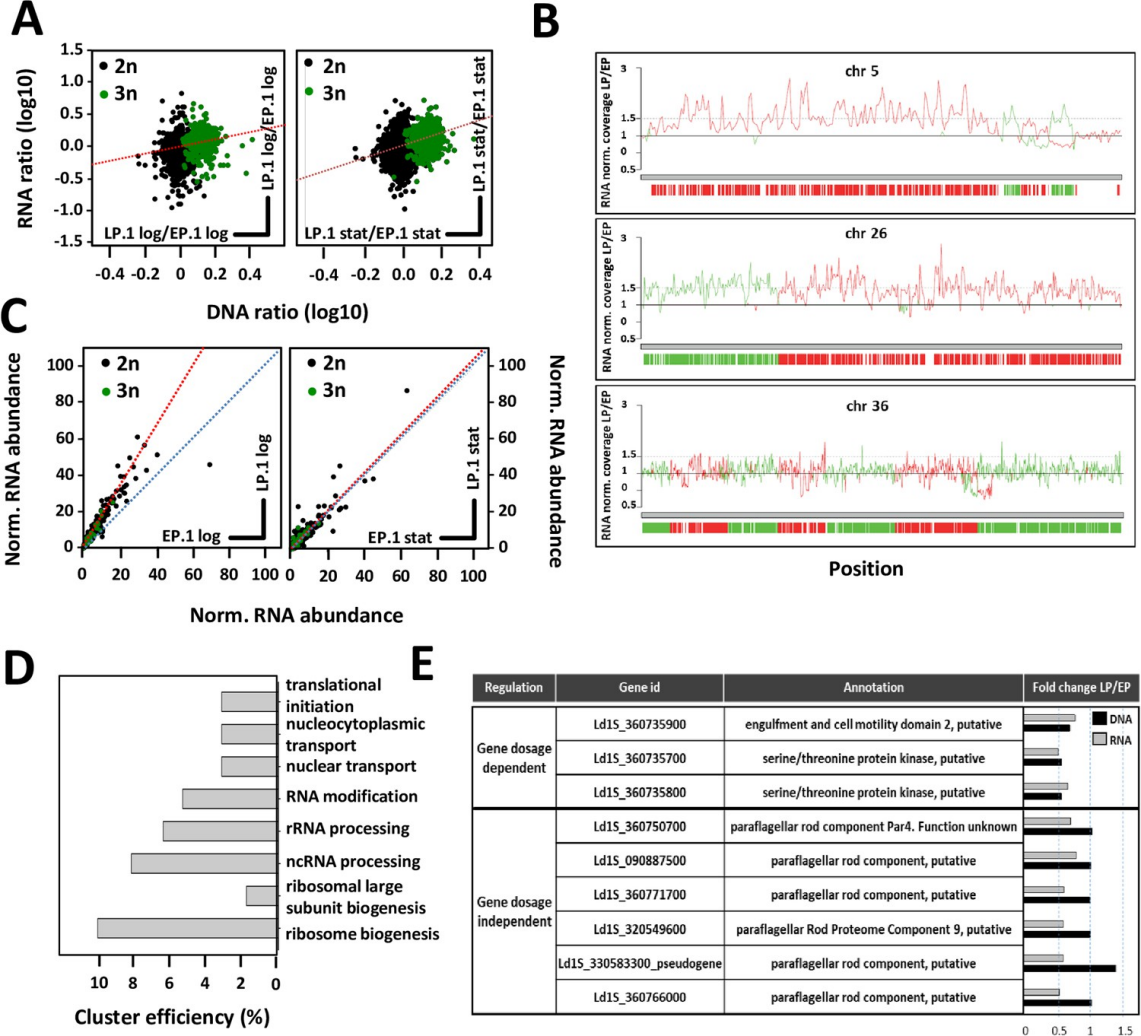
RNA abundance during fitness gain in culture is regulated by gene dosage and post-transcriptional mechanisms. (A) Ratios of DNA and RNA normalized read counts for all genes were plotted for LP.1 log compared to EP.1 log (left panel) and for LP.1 stat compared to EP.1 stat (right panel). Green dots correspond to genes encoded on trisomic chromosomes in LP.1 parasites. The regression line is represented by the dotted red line. Pearson correlation coefficients and p-values were estimated for both ratio plots using SigmaPlot software. For LP.1 log compared to EP.1 log: ρ = 0.341 and p-value < 10^−10^. For LP.1 stat compared to EP.1 stat: ρ = 0.333 and p-value < 10^−10^. (B) Normalized coverage based on the ratio of DNA read counts in LP.1 versus EP.1 for the trisomic chromosomes 5 (upper panel) and 26 (middle panel), and the disomic chromosome 36 (lower panel). The coverage ratio is indicated by the lines, while ORFs are indicated by the vertical bars. The color code reflects the DNA strand on which the ORFs are encoded (see Sheets f and h in S5 Table). (C) Post-transcriptional regulation of transcript abundance. RNA read counts were normalized by DNA read counts and plotted for all genes in LP.1 log compared to EP.1 log (left panel) and EP.1 stat compared to LP.1 stat (right panel). Green dots correspond to genes encoded on trisomic chromosomes in LP.1 (see Sheets a and c in S5 Table). The calculated (red) and expected (blue) regression lines are represented. (D) Cluster efficiency computed from GO term-enrichment analysis for the ‘biological process’ category for 659 gene dosage-independent genes. Transcripts with adj. p-value < 0.01 were considered to determine the ratio of ‘normalized RNA abundance in LP.1/RNA normalized abundance in EP.1’ (see Sheet I in S5 Table for details). Cluster efficiency was calculated based on 274 genes with GO IDs out of the 659 genes that showed at least a 1.2-fold increase in LP.1 normalized RNA abundance compared to EP.1. Only the functional enrichments associated with adj. p-value < 0.05 were considered. (E) Table listing selected gene dosage-dependent and -independent expression changes (from Sheets d and e in S5 Table). The fold change values computed from RNA (grey bars) and DNA (black bars) normalized read counts for LP.1 versus EP.1 log parasites are shown.

The observed changes in transcript abundance during *in vitro* fitness gain may be caused by increased gene dosage due to chromosomal amplification [8,10]. Indeed, comparative genomic analysis of EP.1 and LP.1 parasite revealed aneuploidy for 9 chromosomes during culture adaptation, including trisomies for chromosomes (chr) 5, 23, 26, and 33, which were observed in other *in vitro* evolution experiments [8,10] (S5A Fig and Sheets a and b in S4 Table). We previously observed that tissue amastigotes (in infected hamster spleens) represent a mosaic karyotype, with monosomies and trisomies observed for the analyzed chromosomes, including chr 5 [10]. Based on this result, the reproducible emergence of chr 5 and chr 26 trisomies in different culture adaptation experiments represents a passive, convergent process that relies on the positive selection of pre-existing sub-populations, rather than an active, regulatory process driving karyotypic adaptation. An increased somy score was observed for these chromosomes already in EP.1, indicating a mosaic of disomic and trisomic sub-populations, the latter one showing full penetrance in LP.1. In contrast, the tetrasomy of chr 31 is stable and has been observed in all *Leishmania* species [7] and in *ex vivo L*. *donovani* amastigotes [8,10]. Given the stability of this tetrasomy, regulation of expression via karyotype-dependent gene-dosage effects seems not to apply to chr 31. Thus, the expression changes between EP.1 and LP.1 observed for 144 genes are likely regulated at post-transcriptional levels (Sheet b in S5 Table). Plotting normalized genomic versus transcriptomic read depth ratios for EP.1 and LP.1 log and stat parasites correlated 75% of the up-regulated genes in LP.1 log and 42% in the LP.1 stat promastigotes with amplified chromosomes (Fig 3A, Sheets b and c in S5 Table) affecting various biological processes associated with the LP.1 fitness tradeoff (S6 Fig). Nevertheless, interrogating more specifically the read-depth ratios for trisomic chr 5 and 26 uncovered surprisingly high, gene dosage-independent fluctuations of RNA abundance in EP.1 and LP.1 promastigotes (Fig 3B and Sheets f and g in S5 Table). While a significant fraction of transcripts on the trisomic chromosomes showed the expected 1.5-fold increase in abundance, numerous transcripts either exceeded this increase or on the contrary were expressed at lower-than-expected abundance. Such fluctuations were also observed for the LP.1/EP.1 ratios of disomic chromosomes (see chr 36, Fig 3B as an example and Sheet h in S5 Table). In contrast to the dynamic changes in karyotype, no significant fluctuations in LP.1/EP.1 read depth ratio was observed across the genome mapping the reads to 300 bp genomic bins, thus ruling out episomal or intra-chromosomal amplifications as drivers of culture adaptation, at least during the first 20 passages (i.e. in LP parasites) (S5A Fig, right panel).

We next assessed gene-dosage independent expression changes at genome-wide level by normalizing the RNA-seq read counts to the corresponding DNA-seq reads. Direct comparison of the normalized transcript output in EP.1 versus LP.1 revealed a gene dosage-independent increase in transcript abundance for a large number of genes in LP.1 log (Fig 3C, left panel). No difference was observed for EP.1 and LP.1 stat (Fig 3C, right panel). Genome-independent, post-transcriptional increase in mRNA abundance was observed in LP.1 log parasites for genes annotated for the biological processes ‘rRNA processing’, ‘ribosome biogenesis’, ‘translational initiation’, and ‘nuclear transport’ (Fig 3D and Sheet I in S5 Table). In contrast, manual inspection revealed post-transcriptional decrease in abundance of mRNAs involved in flagellar biogenesis or EP.1-specific, ribosomal components (Fig 3E and Sheet j in S5 Table). Significantly, reduction of both DNA and RNA read depth was observed for two NIMA-related protein kinases on chr 36 that we previously associated with *in vitro* fitness gain [17] (see Fig 3E and Sheet d in S5 Table), firmly linking their depletion to accelerated growth.

In conclusion, the global analysis of the EP.1 and LP.1 transcriptomes uncovers post-transcriptional regulation as an important process that may affect *Leishmania* fitness gain in culture, which can likely buffer against deleterious effects of genome instability and adapt mRNA abundance in a gene dosage-independent manner to a given environment.

### The fitness-adapted proteome is highly robust and enriched for GO terms associated with ribosomal biogenesis and post-transcriptional regulation

**Fig 4:**
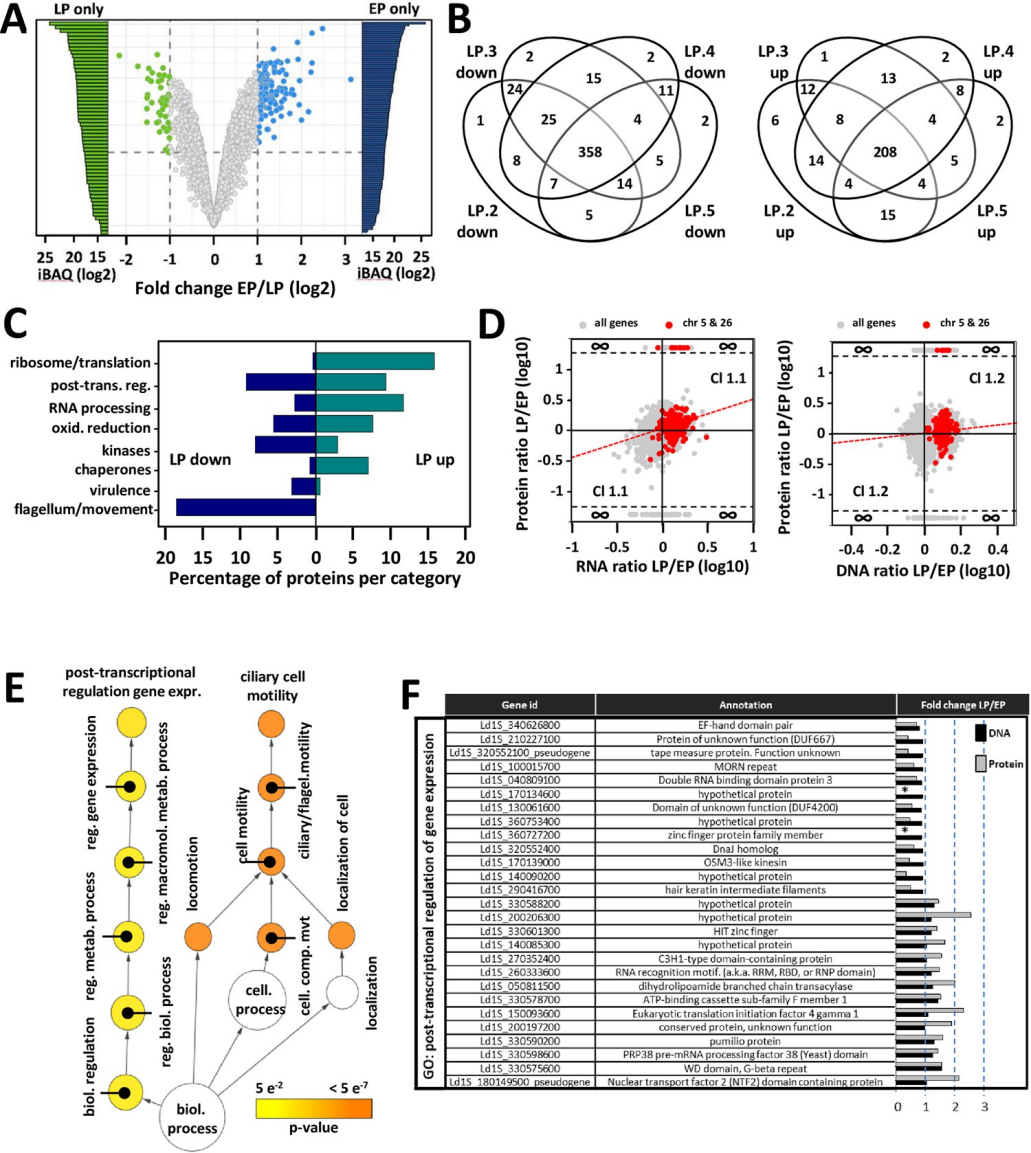
Quantitative analysis of the fitness-adapted proteome. (A) Volcano plot representing changes in protein abundance in EP log (blue dots, mean values of EP.2, EP.3, EP.4 and EP.5 are shown) compared to LP log (green dots, mean values of LP.2, LP.3, LP.4 and LP.5 are shown). Proteins identified by at least two peptides in at least three out of four biological replicates were considered. Colored dots indicate values with FDR < 0.01 and fold changes ≥ 2 (see Sheets b and f in S6 Table). The grey dots indicate non-significant expression changes. The bars indicate unique protein identifications in LP (LP only, green) and EP (EP only, blue) samples, with relative abundance indicated by the iBAQ value. (B) Venn diagram showing the number of proteins quantified and associated to a p-value < 0.01 with increased (left panel) or decreased (right panel) abundance in all four LP log biological replicates (see Sheets c and e in S6 Table). (C) Manual Gene ontology analysis of the proteins shared in all four LP log biological replicates expressed as the percentage of proteins quantified with associated p-value < 0.01 for the indicated gene categories (see Sheets h and I in S6 Table). (D) Double ratio plots comparing the fold changes computed for each gene between LP and EP log parasites for RNA (x-axis) versus protein (y-axis) (left panel) and DNA (x-axis) versus protein (y-axis) (right panel). All proteins with LFQ values were considered to determine the protein ratio LP/EP (see Methods). Grey dots represent all proteins and red dots those encoded on trisomic chromosomes 5 and 26 (see Sheets a and h in S7 Table). Cluster 1.1 and 1.2 (Cl 1.1 and Cl 1.2) includes proteins whose change in abundance shows the same tendency compared to RNA abundance or gene dosage, respectively. The regression line is represented by the dotted red line. The Pearson correlation coefficients and the p-values were estimated for both ratio plots using Sigma Plot software. For protein versus RNA ratio plot: ρ = 0.349 and p-value < 10^−10^. For protein versus DNA ratio plot: ρ = 0.145 and p-value < 10^−10^. (E) Graphical representation of the GO term-enrichment analysis for the category ‘biological process’ for the 452 proteins from cluster 1 (common proteins between clusters 1.1 and 1.2), which includes 201 proteins with a GO annotation (cluster 1, see right panel D and Sheet c in S7 Table). The size of the circle is indicative of the number of genes falling in each category and the color ranging from yellow to orange indicates the p-values associated as indicated in the legend. Only proteins quantified in all four biological replicates for each condition and associated with a p-value < 0.01 were considered for the GO analysis (see Sheet b in S7 Table). (F) Table listing selected genes associated with the GO term ‘post-transcriptional regulation of gene expression’ from the GO enrichment analysis presented in panel E (see Sheet c in S7 Table for details). Their respective fold change values computed from Protein LFQ intensities (grey bars) and DNA normalized read counts (black bars) for LP versus EP log parasites are represented. *proteins exclusive to EP log parasites.

We applied a label-free, quantitative proteomics approach to assess how genomic and post-transcriptional adaptation during *in vitro* fitness gain impact protein abundance. Analyzing four independent, biological replicates of EP and LP strains (termed EP.2-5 and LP.2-5, S7 Fig) identified a total of 6,050 proteins considering all samples, including 59 and 110 proteins that were exclusively detected in LP and EP parasites, respectively (Fig 4A and Sheets b and f in S6 Table). Considering all proteins that showed a statistically different abundance (Fig 4B, Sheets c and e in S6 Table), the majority of differentially expressed genes were shared in all four independent LP strains (566 of 788 total, 71%). These data reveal a surprising convergence of the fitness-adapted proteomes despite possible karyotypic variations between strains (S5B Fig), and inform on common pathways that are under convergent selection in LP strains during *in vitro* fitness gain. Just as observed on RNA levels, flagellar biogenesis is clearly under negative selection during culture adaptation, with reduced protein abundance observed for 46 proteins linked to flagellum and motility encoded on 24 chromosomes (Fig 4C and Sheet I in S6 Table). Another key process associated with adaptation was translation: 27 proteins encoded on 13 different chromosomes were under positive selection in LP strains (e.g. various ribosomal proteins of the 39S, 40S, 60S, L22e, and S25 families, or the ribosomal assembly protein RRB1), while only two RNA binding proteins encoded on two chromosomes were under negative selection in the same parasites (Fig 4C and Sheets h and i in S6 Table).

We next assessed the level of correlation between protein abundance, gene dosage variation and transcript abundance to gain further insight into regulatory mechanisms underlying *Leishmania* fitness gain in culture. Even though the proteomics data set was obtained with four independent biological replicates (EP.2-5 and LP.2-5), the highly reproducible nature of the chr 5 and chr 26 trisomies observed in all our previous experimental evolution experiments (S5B Fig) provided a useful benchmark to assess correlations between the different data sets for at least these chromosomes. Our systems comparison suggests the presence of three different regulatory clusters for chr 5 and 26: one cluster (common proteins from clusters 1.1 and 1.2, see Sheets j and l in S7 Table) includes 34 proteins whose change in abundance correlates to gene dosage and RNA abundance (Fig 4D left and right panels, upper right and lower left quadrants), including three DNA J proteins, the chaperonin 10, a HSP70 like protein and BiP, suggesting that increased stress resistance could be a potential driving force for the selection of these aneuploidies (Sheets j and l in S7 Table). Possible post-transcriptional regulation is observed for the surface antigen-like protein (Ld1_ 050818900), whose level only correlates with mRNA abundance but not gene dosage. Finally, cluster 3 represents 5 proteins whose levels do not correlate with mRNA abundance, which are either regulated at translational levels or by protein turn-over (Sheet k in S7 Table). Thus, the increase in protein abundance is the combined result of gene dosage and mRNA abundance for the vast majority of proteins (83%) encoded on trisomic chr 5 and 26.

Gene ontology analysis of the 452 proteins that fall into regulatory cluster 1.2 (as defined by the upper right and lower left quadrants of Fig 4D, right panel) revealed a strong enrichment for the term ‘post-transcriptional regulation of gene expression’ supported by 27 proteins (Fig 4E and Sheets b and c in S7 Table), which corresponds to 15% of all proteins that show increased abundance in LP parasites (S7D Fig and Sheets d and e in S7 Table). This enrichment is driven by the coordinated increase in expression of various proteins with known functions in RNA turnover (e.g. pumilio-domain protein encoded by Ld1S_330590200) and a series of proteins previously linked to post-transcriptional regulation in *T*. *brucei* such as EIF4G1 or PRP38 pre-mRNA processing factor (Sheet d in S7 Table) [56–58]. In addition, the enrichment for the GO term ‘ciliary cell motility’ is driven by the under-representation of this process in the LP proteome, supported by 20 proteins (or 18%) of all proteins showing less abundance in LP (S7E Fig and Sheets b, c, f and g in S6 Table).

In conclusion, the *Leishmania* proteome undergoes reproducible, gene dosage-dependent and -independent changes during fitness gain *in vitro*. The robustness of proteomic adaptation indicates the presence of regulatory mechanisms that compensates for the genetic and transcriptomic variability between independent LP strains. At least under our experimental conditions, gene dosage-dependent changes modulate post-transcriptional regulation, which results in stabilization of various transcripts implicated in rRNA processing and ribosomal biogenesis. Thus, just as observed on transcript levels (Figs 2 and 3), the proteomic results too suggest the formation of fitness-adapted ribosomes, which in turn may control the robustness of the proteome. The role of ncRNAs in ribosomal biogenesis [59] primed us in the following to carry out a dedicated small RNome analysis in EP and LP parasites to further assess the generation of specialized ribosomes.

### Mapping the *Leishmania* non-coding transcriptome correlates snoRNA expression and rRNA modification to *Leishmania* fitness gain *in vitro*

**Fig 5:**
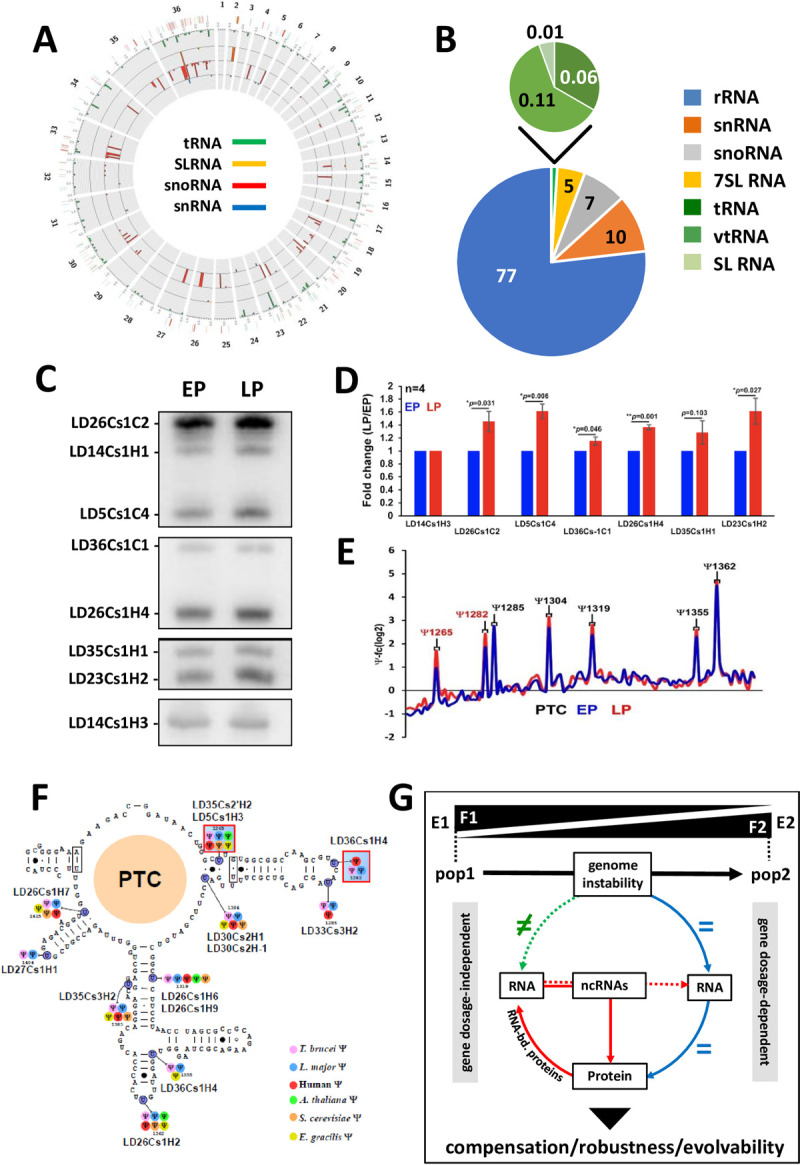
The fitness tradeoff in LP promastigotes correlates with snoRNA expression changes and increased rRNA pseudouridinylation levels. (A) Genomic map of *L*. *donovani* Ld1S ncRNA genes. (B) Composition of the small RNome identified in EP parasites. (C) Northern blot analysis of selected snoRNAs, Ld14Cs1H3 was used as loading control. Two representative northern blots out of four are presented. The analyses were performed with two independent cultures derived from the same frozen stock (see S10 Fig for details). (D) Histogram plot representing the fold changes between LP (red bars) and EP (blue bars) log parasites corresponding to densitometric analysis of the signals shown in (C). Data are presented as mean ± S.E.M. Student’s t-test was performed to determine the p-value. * p-value < 0.05; ** p-value < 0.005. (E) Line graph of the fold change in rRNA pseudouridinylation level (Ψ-fc, log2) in EP (blue line) and LP parasites (red line). The shown result is representative of three independent cultures derived from the same frozen stock (see S10 Fig for details). Positions where the Ψ level is increased in all three replicates are indicated in red. (F) The location of Ψ sites in the rRNA is depicted on the secondary structure. Hypermodified sites are highlighted in red squares. The snoRNAs guiding each Ψ are indicated. The color code for each Ψ site is indicative of the organism where it was already reported. (G) Model of *Leishmania* evolutionary adaptation. Different environments (E1, E2) select for different fitness traits (F1, F2), which modify the parasite population structure (pop 1, pop 2). In the absence of transcriptional regulation, *Leishmania* exploits genome instability to generate changes in gene dosage via chromosome and gene copy number variations. These changes are either correlated (blue arrows) or not (green arrow) to changes in transcript and protein abundance. The gene dosage-regulated transcriptome and proteome (right panel) is highly enriched for the GO term ‘post-transcriptional regulation of gene expression’ and thus likely regulates gene dosage-independent changes in RNA abundance (red arrow, left panel). The enrichment of these transcripts in ncRNAs in turn can control RNA stability and translatability by guiding modifications of mRNA or rRNAs. This allows for (i) compensation of deleterious gene dosage effects, (ii) phenotypic robustness despite genetic heterogeneity, and (iii) maintenance of evolvability despite selection pressure.

Non-coding (nc) RNAs such as small nuclear (sn), small nucleolar (sno), ribosomal (r) or transfer (t) RNAs play essential roles in post-transcriptional regulation and translational control [24,60]. While our data suggested an important role of these regulatory processes in genome-independent fitness gain in culture (see above), they did not inform on underlying ncRNAs as our RNAseq analyses used poly (A+)-enriched mRNA. We therefore performed a dedicated analysis of the small RNome in EP.1 and LP.1 *L*. *donovani* parasites. We first annotated our PacBio LD1S reference genome for ncRNAs using bioinformatics approaches (ortholog mapping, *de novo* annotation) as well as unmapped RNAseq reads of post-ribosomal supernatants that are enriched in ncRNAs (S8 Fig). These efforts established a very first repertoire of ncRNAs in any *Leishmania* species and identified 1,504 genes encoding for snoRNA organized in 42 clusters on 24 chromosomes, 83 tRNA genes, 12 snRNA genes and 140 SL RNA genes (S1 Table and Fig 5A and 5B). Considering the trisomic chromosomes, we found 269 snoRNA genes on chr 5, 22 on chr 23, 160 on chr 26, and 193 on chr 33. We investigated more specifically the role of snoRNAs in LP.1 fitness gain in culture given the enrichment of the fitness-adapted transcriptome in the GO terms ‘ribosomal biogenesis’ and ‘rRNA processing’ (see Fig 2C). snoRNAs guide specific modifications of rRNA, such as methylation and pseudouridylation, which in turn change the specificity of the ribosome towards certain mRNAs and thus control translation [61]. We prepared whole cell lysates from both EP.1 log and LP.1 log parasites, removed the ribosomes by ultracentrifugation, and prepared libraries from the post-ribosomal supernatant. From 174 detected snoRNAs, 93 showed a more than 2-fold change in LP.1 compared to EP.1, revealing a global increase of snoRNA abundance during culture adaptation (S8 Table). Increased abundance was confirmed for 7 out of the 8 snoRNAs probed by Northern blot analysis of the PRS (Fig 5C and 5D).

Next, we examined if the increase in snoRNA abundance affected the level of rRNA pseudouridylation (Ψ) by applying a modified RNAseq protocol (termed Ψ-seq) using total RNA from EP.1 and LP.1. We detected two hyper-modified rRNA sites in all three biological replicates at positions Ψ1265 and Ψ1282 inside the peptidyl transferase center (PTC) (Fig 5E), which correlated with the increased abundance of the corresponding snoRNAs that guide these modifications (Fig 5F and S8 Table) [62]. Our data thus provide a first link of snoRNA expression and rRNA modification to *Leishmania* fitness gain in *in vitro* culture, which further supports the possibility of fitness-adapted ribosomes and suggests translational control–in addition to genomic and post-transcriptional adaptation–as yet another, gene dosage-independent mechanism likely linked to *Leishmania* evolutionary adaptation.

## Discussion

A common hallmark of all microbial pathogens is their capacity to adapt to unpredictable fluctuations in their host environments through an evolutionary process, where genetically heterogenous individuals constantly compete for survival [63]. Here we combined experimental evolution and integrative systems approaches to uncover mechanisms of fitness gain in the human pathogen *Leishmania donovani*. Our study provides new evidence that these parasites combine regulatory processes at genomic, post-transcriptomic and translational levels to establish highly robust fitness phenotypes while maintaining genetic heterogeneity thereby avoiding genetic death.

In the absence of classical, promoter-driven control of gene expression, *Leishmania* relies on alternative mechanisms to regulate transcript and protein abundance, including regulated mRNA turn over and translational control [5,64]. These parasites further use a highly unusual, genomic form of gene expression regulation, where changes in chromosome and gene copy number control transcript abundance via gene dosage [7–10]. Previous studies allowed us to link these forms of genome instability to fitness gain *in vitro* as judged by the highly reproducible karyotypic changes observed during culture adaptation in independent clinical and animal-derived *L*. *donovani* isolates [8,10,20]. Positive selection of chromosome amplification is further sustained by the independent evolutionary experiments conducted in this study, which once more revealed amplification of chromosomes 5 and 26 as key drivers of *in vitro* fitness gain. Such karyotypic changes are not exclusive to culture adaptation but have been documented in *L*. *donovani* tissue amastigotes [10], and in drug resistant clinical *Leishmania* isolates [13]. Similar to stress-adaptation in fungi [65], karyotypic changes thus may provide the genetic diversity required for *Leishmania* to evolve beneficial phenotypes in response to environmental change. However, such structural mutations simultaneously affect the expression level of hundreds of genes, raising questions on the nature of the coding sequences that drive karyotypic selection during parasite adaptation, and on the mechanisms that suppress deleterious gene dosage effects while preserving beneficial ones. Applying an integrative systems approach on promastigote parasites during culture adaptation (early passage, EP and late passage, LP) allowed us to address these important open questions.

Comparative genomic, transcriptomic and proteomic analyses of EP and LP promastigote parasites revealed a gene dosage-dependent increase in mRNA and protein abundance for genes implicated in RNA turnover, including RNA-binding proteins known to regulate mRNA stability [66], pumillo domain proteins known to regulate ncRNA abundance [67], and a series of proteins that were associated with trypanosomatid mRNA-binding and post-translational regulation in recent, genome-wide functional screens [56–58]. Positive selection of chromosome amplifications during *L*. *donovani* culture adaptation thus is likely driven by genes that establish an adaptive, post-transcriptional interface. This interface may regulate differential mRNA stability during fitness gain in culture, which can compensate for deleterious gene dosage effects by selective mRNA degradation, while at the same time boosting the expression of beneficial genes conferring stability to selected mRNAs. Assessment of gene-dosage-independent expression changes indeed correlated both increased and decreased mRNA abundance to the observed fitness phenotype. In the absence of transcriptional control in *Leishmania*, these gene dosage-independent changes in mRNA abundance must be regulated by differential RNA stability. A number of transcripts implicated in flagellar biogenesis showed reduced stability during culture adaptation, which correlated with reduced mobility (see [17]). This coordinated process likely involves shared *cis*-regulatory sequence elements in the transcripts’ 3’UTR that are recognized by the RNA-binding proteins [5]. Loss of flagellar function associated to *L*. *donovani in vitro* fitness gain has been observed in other independent evolutionary experiments [17] and thus represents a reproducible, convergent phenomenon that may liberate ATP for energetically demanding processes that are under positive selection during culture adaptation. Indeed, a large number of transcripts implicated in highly energy-demanding ribosomal biogenesis and translation were stabilized in LP parasites. The differential regulation of mRNA abundance observed in our experimental evolution system thus establishes a first link of post-transcriptional regulation to *Leishmania* fitness gain in our *in vitro* setting.

In culture, fitness (defined as reproductive success of a given population) is largely synonymous to proliferation, which depends on the number of ribosomes and the cell’s translational potential [68]. While the fitness-adapted transcriptome is indeed characterized by increased expression in ribosomal components thus fueling the need for more ribosomes, the differential expression of various 40S and 60S ribosomal protein isoforms in LP compared to EP parasites further suggests that adaptation is linked to a qualitative, ribosomal changes (see S2 and S6 Tables). Such dynamic regulation of ribosomal biogenesis may give rise to specialized ribosomes, which not only may increase translation efficiency in these fast-growing LP parasites, but could also control translation of unwanted mRNAs, thus providing an additional filter (next to differential RNA stability) to eliminate toxic gene dosage effects. The existence of such structurally distinct, specialized ribosomes has been observed in *Plasmodium* spp., where stage-specific expression of certain rRNA isoforms allows for the establishment of A-type (asexual stage specific) and S-type (sporozoite specific) ribosomes [69,70]. Likewise, stage-specific modification of rRNA has been linked to the transition of *T*. *brucei* from the procyclic insect to the mammalian bloodstream forms [23]. Finally, changes in expression and modification of different rRNA genes, ribosomal proteins, and translation factors indeed can control preferential translation of different subsets of mRNAs in other organisms [71], including *Dictyostelium discoideum* [72,73], zebrafish development [74], or cancer [75].

Conducting a dedicated analysis of the *L*. *donovani* non-coding (nc) RNome, we have provided further support to the existence of such fitness-adapted ribosomes in *Leishmania*. First, we observed post-transcriptional upregulation of a large number of snoRNAs and five (out of a total of 9) pseudouridylate synthases in LP compared to EP promastigotes (see S5 Table). Second, these snoRNA expression changes correlated to changes in the pseudouridinylation (Ψ) profile of the rRNA peptidyl transferase center (PTC) that catalyzes peptide bond formation and peptide release [76]. Similar Ψ hyper-modification of rRNA was previously observed in bloodstream form trypanosomes and likely contributes to stage-specific adaptation [23]. Given the high coding density of chr 5 and chr 26 for snoRNAs and the functional enrichment of these chromosomes for GO term ‘rRNA processing’ (see S5C Fig), it is interesting to speculate that their convergent amplification in all our evolution experiments may be driven by their ncRNA content and their requirement for fitness-adapted translation. Indeed, defects in rRNA pseudouridylation affect ribosomal ligand binding and translational fidelity in eukaryotic cells [77], and changes in PTC modification were shown to affect both the ribosome structure and activity in yeast [78]. The combination of (i) different rRNA isoforms, (ii) hundreds of snoRNAs and differentially modified rRNA sites, (iii) diverse 40S and 60S ribosomal proteins, and (iv) the formation of different translation complexes [79–81] defines a vast ribosomal landscape in *Leishmania*. Translational control via fitness-adapted ribosomes likely fine-tunes expression and provides proteomic and phenotypic robustness to adapting parasite populations, which thus can maintain genetic diversity and evolvability despite constant natural selection [10].

In conclusion, our data uncover *Leishmania* evolutionary adaptation as an emergent property of a highly complex process that integrates variations in gene dosage with correlating changes in transcript abundance for genes implicated in post-transcriptional regulation and ribosomal biogenesis, which may compensate for toxic gene dosage effects via differential RNA turn-over and translational regulation, respectively (see Fig 5G). Even though our results are largely correlative in nature, our model is supported by the convergence of the genomic, transcriptomic and proteomic signals we observed between independent populations, which thus are the result of natural selection rather than random genetic drift. Our findings challenge the current genome-centric approach to *Leishmania* epidemiology and suggest the *Leishmania* non-coding RNome as well as regulatory circuits at transcriptional and translational levels as potential novel sources for biomarker discovery in clinical settings. Finally, our model may be of relevance to other pathogenic systems that gain fitness through genome instability, including fungal infection and cancer.

## Supporting information

S1 Fig Parasite growth and macrophage infection studies. (A) Comparison of EP.1 stat (open circle) and LP.1 stat (grey circles) infectivity. Mean number of parasites per 100 macrophages +/-SD (left panel) and the percentage of infection (right panel) from three biological replicates are shown. (B) Comparison of EP.1 stat (open circle) and EP.1 meta (open triangle) infectivity. Mean number +/-SD of parasites per 100 macrophages (left panel), the percentage of infection (middle panel) and the relative number of intracellular parasites (right panel) from a representative experiment out of three replicates are shown. * indicates p-value ≤ 0.05. (C) Histogram plots representing the generation time of EP and LP parasites originated from an independent evolutionary experiment with parasites expressing luciferase (EP.luc and LP.luc) (left panel). Replication rate in infected macrophages for the EP.luc and LP.luc parasites calculated between day 1 and day 6 after infection (right panel).
(TIF)

S2 Fig Morphological analysis of EP and LP metacyclic-enriched parasite fractions. (A) Micrographs of representative EP.1 metacyclic isolated from the sand fly thoracic part (upper left image), Ficoll-enriched EP.1 (upper middle image) and LP.1 metacyclic-like parasites (upper right image) from stationary culture. Broad field images of EP.1 and LP.1 metacyclic enriched parasites are presented in the lower right and left images. (B) Quantitative morphological analysis of stationary-phase and metacyclic-enriched parasite populations. The box plots show the median values and the upper and lower quartiles for body length (left panel), body width (middle panel) and flagellum length (right panel). (C) Distribution of the indicated promastigote forms in EP.1 and LP.1 Ficoll-enriched metacyclic fractions.
(TIF)

S3 Fig Transcriptomic analysis of EP.1 and LP.1 parasites. (A) Principal Component Analysis of EP.1 and LP.1 parasites from logarithmic (log) and stationary (stat) phase cultures, and after metacyclic enrichment (meta). (B) Ratio plots of normalized RNA abundance for EP.1 stat compared to EP.1 meta. Dark blue and dark cyan dots represent respectively gene expression changes with FC > 1.5 and adjusted p-value ≤ 0.01; black dots correspond to gene expression changes with adjusted p-value > 0.01. (C) Histogram plot representing the number of genes showing decreased transcript abundance in LP.1 log for the indicated gene categories (see Sheet j in S2 Table). (D) Principal Component Analysis of RNAseq for two independent EP and LP biological replicates from logarithmic phase culture. Samples are identified according to S9 Fig. (E) Histogram plot showing the number of genes with decreased and increased transcript abundance in LP log parasites from two independent transcriptomic analyses. Cyan and blue histogram bars represent the evolutionary experiment presented in Fig 2 (EP.1 and LP.1), grey bars correspond to the second RNAseq data set corresponding to EP.8, EP.9, LP.9 and LP.10 samples (see S9 Fig and Sheets c and e in S3 Table for detail).
(TIF)

S4 Fig Functional enrichment analysis of EP.1 and LP.1 parasites. (A, B) Graphical representations generated with the BiNGO plugin of the Cytoscape software package for the GO term-enrichment analysis performed with the transcripts showing statistically significant increased abundance in LP.1 log (A) and LP.1 stat (B) (see Sheet o in S2 Table). The size of the circle is indicative of the number of genes falling in each category and the color ranging from yellow to orange indicates the p-values associated as indicated in the legend. (C) Histogram plot representing the number of genes showing decreased transcript abundance in LP.1 stat for the indicated gene categories (see Sheet n in S2 Table).
(TIF)

S5 Fig Comparative genome analysis of EP and LP parasites from independent evolutionary experiments. (A) Chromosome somy levels of EP.1 and LP.1 promastigotes. Chromosome read-depth distributions are shown in boxplots depicting the median and the upper and lower quartiles (left panel). Genome-wide coverage ratios (y axes) between LP.1 and EP.1 (right panel). Genome-wide coverage ratio (x- axis) between EP.1 and LP.1. The y axis reports the position of the genomic windows along the chromosomes. Dots represent genomic windows of 300 bases. (B) Violin plot computed from three independent evolutionary experiments representing the somy score distribution for each chromosome. In red are highlighted chr 5 and 26 that are trisomic in all three experiments (LP.1, LP.6 and LP.7). (C) Enrichment analysis of the aneuploid chromosomes for the GO categories ‘molecular function’ (chr 1) and ’biological process’ (5, 12, 23, 26, 31 and 33). The bars correspond to the cluster efficiency computed from GO term-enrichment analyses (see Sheet d in S4 Table).
(TIF)

S6 Fig GO analysis of gene dosage-dependent and -independent changes in RNA abundance. (A, B) Enrichment analysis for the GO category ’biological process’. RNA read counts were first normalized by DNA read counts to estimate the ratio of normalized RNA abundance between LP.1 and EP.1 (see Sheet i in S5 Table). (A) Histogram showing the cluster efficiency for 1,104 genes that show dosage dependent changes in mRNA abundance (ratio from 0.8 to 1.2), including 463 genes that are annotated with a GO term. (B) Histogram showing the cluster efficiency for 1,192 genes that show dosage in-dependent changes in mRNA abundance (ratio < 0.8), including 510 genes that are annotated with a GO term and show a decrease in RNA read counts after normalization to DNA read counts in LP.1 log parasites.
(TIF)

S7 Fig Quantitative proteomics analysis. (A) Box plots representing the median ratio and the upper and lower quartiles of the LFQ intensity values for all LP biological replicates (LP.2, LP.3, LP.4 and LP.5) compared to the median of all EP replicates (EP.2, EP.3, EP.4 and EP.5). (B) Cluster analysis of all EP and LP samples (Ward method). (C) Ratio plot representing the mean LFQ intensity value between EP and LP for each individual, quantified protein. The experimental and the expected regression lines are shown in red and blue respectively. (D, E) Cluster efficiency for the GO category ‘biological process’ for proteins from cluster 1 whose abundance correlates with increased (D) or decreased normalized DNA read counts (E) in LP log parasites. Only proteins quantified with a p-value < 0.01 were considered for the GO term enrichment analysis (see Sheets e and g in S7 Table). (F) Table listing selected genes associated with the GO term ‘ciliary cell motility’ from the GO enrichment analysis presented in Fig 4E. Their respective fold change values computed from Protein LFQ intensities (grey bars) and DNA normalized read counts (black bars) for LP versus EP log parasites are represented.
(TIF)

S8 Fig Enrichment of small RNAs obtained from post-ribosomal supernatants of EP promastigotes. 2x10^9^ cells were disrupted by nitrogen cavitation under low salt concentration (150 mM KCl in the presence of high MgCl_2_ (10 mM) followed by ribosome extraction using high KCl (300 mM). The ribosomes were removed by centrifugation at 35,000 rpm for 2h. Two μg of RNA from total lysate (Total RNA) and PRS sample were separated on a 10% polyacrylamide gel and stained with ethidium bromide.
(TIF)

S9 Fig Overview chart of strains used in this study. Each hamster infected with *L*. *donovani* parasites was identified by the cage number and is the source of amastigotes (AMA) for conversion to promastigotes. Early passage promastigotes (EP) and late passage promastigotes (LP) used for the genomic (DNA), transcriptomic (RNA), small RNome and transcriptome-wide mapping of pseudouridine sites (RNome & Ψ-seq), proteomic (Protein) and phenotypic analyses are identified. The parasites marked by an asterisk (*) were frozen at passage 2 and passage 20.
(TIF)

S10 Fig Experimental flow chart. Strains issued from independent experimental evolution assays are identified by number (i.e. EP.1 and LP.1 are the strains resulting from experiment 1). Frozen stocks of EP.1, LP.1, EP.luc and LP.luc were prepared. The stage-specific expression analysis was therefore performed starting from three frozen aliquots prepared at passage 2 (EP.1) and passage 20 (LP.1). Each of the frozen parasites was used to prepare RNA extracts from log and stationary growth culture and from enriched metacyclic forms. Likewise, phenotypic analyses performed with EP.1, LP.1, EP.luc and LP.luc started from frozen aliquots for each replicate.
(TIF)

S1 Table Transcriptomic read counts of EP and LP RNAseq analyses. Index. Detailed information on samples and analyses presented in the table. a Sheet in S1 Table. RNAseq complete data set.
(XLSX)

S2 Table EP.1 versus LP.1 comparative transcript profiling. Index. Detailed information on samples and analyses presented in the table. a Sheet in S2 Table. EP log vs EP stat. RNAseq data analysis for genes with adjusted p-values < 0.01. b Sheet in S2 Table. EP log vs EP meta. RNAseq data analysis for genes with adjusted p-values < 0.01. c Sheet in S2 Table. EP stat vs EP meta. RNAseq data analysis for genes with adjusted p-values < 0.01. d Sheet in S2 Table. LP log vs LP stat. RNAseq data analysis for genes with adjusted p-values < 0.01. e Sheet in S2 Table. EP log vs LP log. RNAseq data analysis for genes with adjusted p-values < 0.01. f Sheet in S2 Table. EP stat vs LP stat. RNAseq data analysis for genes with adjusted p-values < 0.01. g Sheet in S2 Table. LP log up. Transcripts more abundant in LP.1 log compared to EP.1 log. h Sheet in S2 Table. LP log down. Transcripts more abundant in EP.1 log compared to LP.1 log. i Sheet in S2 Table. LP log up Category. List of genes more abundant in LP.1 log manually assigned to a category. j Sheet in S2 Table. LP log down Category. List of genes more abundant in EP.1 log manually assigned to a category. k Sheet in S2 Table. LP stat up. Transcripts more abundant in LP.1 stat compared to EP.1 stat. l Sheet in S2 Table. LP stat down. Transcripts more abundant in EP.1 stat compared to LP.1 stat. m Sheet in S2 Table. LP stat up Category. List of genes more abundant in LP.1 stat manually assigned to a category. n Sheet in S2 Table. LP stat down Category. List of genes more abundant in EP.1 stat manually assigned to a category. o Sheet in S2 Table. GO biological process. GO term enrichment analysis for the category ’biological process’ for genes with FC LP.1 log/EP.1 log > 1.5, genes with FC LP.1 stat/EP.1 stat > 1.5 and genes with FC EP.1 stat/LP.1 stat > 1.5. p Sheet in S2 Table. Flagellum-Mvt EPlog_stat_meta. List of genes manually assigned to the category flagellum and movement during the transition from EP log to stat and EP stat to meta.
(XLSX)

S3 Table EP versus LP comparative transcript profiling (EP.8, EP.9, LP.8 and LP.10). Index. Detailed information on samples and analyses presented in the table. a Sheet in S3 Table. EP log vs LP log. RNAseq data analysis for all genes. b Sheet in S3 Table. LP log down. Transcripts more abundant in EP log compared to LP log. c Sheet in S3 Table. LP log down Category. Genes overexpressed in EP log organized by gene categories. d Sheet in S3 Table. LP log up. Transcripts more abundant in LP log compared to EP log. e Sheet in S3 Table. LP log up Category. Genes overexpressed in LP log organized by gene categories.
(XLSX)

S4 Table Genomic analyses of EP and LP parasites. Index. Detailed information on samples and analyses presented in the table. a Sheet in S4 Table. DNA EPlog-LPlog. Read counts and normalized read counts per gene for both EP.1 and LP.1 parasites collected during exponential growth phase. b Sheet in S4 Table. ChrMedianCov_EP.1-LP.1. Median somy score for all chromosomes for the evolutionary experiment 1. c Sheet in S4 Table. Chr Median Cov_Evol Exp. Median somy score for all chromosomes computed from LP.1, LP.6 and LP.7 evolutionary experiments. d Sheet in S4 Table. GOanalysis_trisomicChr. GO term enrichment in ’molecular function’ or ’biological process’ for the aneuploid chromosomes (1, 5, 12, 23, 26, 33 and 31).
(XLSX)

S5 Table Analysis of gene copy number-independent changes in RNA abundance. Index. Detailed information on samples and analyses presented in the table. a Sheet in S5 Table. All RNAnorm LP ogvs EPlog. RNA normalized abundance determined for EP.1 and LP.1 log parasites for all genes. b Sheet in S5 Table. RNAsig_RNAnorm_LPlogvs EPlog. RNA normalized abundance determined for EP.1 and LP.1 log parasites for genes whose RNA read counts were associated with adj p-value < 0.01). c Sheet in S5 Table. All_RNA&DNA_LPstatvs EPstat. RNA normalized abundance determined for EP.1 and LP.1 stat parasites for all genes. d Sheet in S5 Table. Gene dosage dependent. List of genes for which the ratio of normalized RNA read counts / normalized DNA read counts # 1. e Sheet in S5 Table. Gene dosage independent. List of genes for which the ratio of normalized RNA read counts / normalized DNA read counts is <0.8 or >1.2. f Sheet in S5 Table. RNAnormCoverChr5_LPlog-EPlog. RNA normalized coverage determined between EP.1 log and LP.1 log for the chromosome 5. g Sheet in S5 Table. RNAnormCoverChr26_LPlog-EPlog. RNA normalized coverage determined between EP.1 log and LP.1 log for the chromosome 26. h Sheet in S5 Table. RNAnormCoverChr36_LPlog-EPlog. RNA normalized coverage determined between EP.1 log and LP.1 log for the chromosome 36. i Sheet in S5 Table. GO_RNA norm Abundance_BP. GO term enrichment analyses for the category biological process. j Sheet in S5 Table. Categories. List of gene dosage independent genes manually assigned to a category.
(XLSX)

S6 Table Proteomic analysis of EP and LP log parasites. Index. Detailed information on samples and analyses presented in the table. a Sheet in S6 Table. Protein_EPvsLP_allquantified. Proteomic data set for all the proteins quantified. b Sheet in S6 Table. LPup FC≥2. Proteins more abundant in LP in at least three out of four biological replicates c Sheet in S6 Table. LP up allFC_allRep_p-val<0.01. Proteins more abundant in all four LP log samples. d Sheet in S6 Table. LP up FC≥2-allRep. Proteins up in LP quantified in all four biological replicates. e Sheet in S6 Table. LPdown_allFC_allRep-pval<0.01. Proteins less abundant in LP in all four biological replicates. f Sheet in S6 Table. LPdown FC≥2. Proteins less abundant in LP quantified in at least three out of four biological replicates. g Sheet in S6 Table. LPdown FC≥2_allRep. Proteins down in LP quantified in all four biological replicates. h Sheet in S6 Table. LP up Category. List of proteins more abundant in all LP biological replicates manually assigned to a category. i Sheet in S6 Table. LP down Category. List of proteins less abundant in LP quantified in all biological replicates manually assigned to a category.
(XLSX)

S7 Table Correlation between protein abundance, gene dosage variation and transcript abundance. Index. Detailed information on samples and analyses presented in the table. a Sheet in S7 Table. DNAxProteinAllRep. Correlation table between DNA and Protein abundance. b Sheet in S7 Table. DNAxProtAllRepSig_cluster1.2. Correlation table between DNA and Protein abundance for cluster 1.2. c Sheet in S7 Table. GO_DNAxProtein_cluster1.2. GO term enrichment analysis for the category ’biological process’ for the proteins from cluster 1.2. d Sheet in S7 Table. DNAxProt_cluster1.2_LPup. Correlation table between DNA and Protein abundance for proteins more abundant in LP and included in cluster 1.2. e Sheet in S7 Table. GO_DNAxProt_cluster1.2_LPup. GO term enrichment analyses for the category ’biological process’ for the proteins from cluster 1.2 more abundant in LP. f Sheet in S7 Table. DNAxProt_cluster1.2_LPdown. Correlation table between DNA and Protein abundance for proteins less abundant in LP and included in cluster 1.2. g Sheet in S7 Table. GO_DNAxProt_cluster1.2_LPdown. GO term enrichment analyses for the category ’biological process’ for the proteins from cluster 1.2 less abundant in LP. h Sheet in S7 Table. RNAxProtAllRepSig. Correlation table between DNA, RNA and Protein abundance. i Sheet in S7 Table. DNAxRNAxProt_chr5&26. Correlation table between DNA, RNA and Protein abundance for proteins from chromosomes 5 and 26. j Sheet in S7 Table. DNAxRNAxProt_chr5&26_cluster1. Correlation table between DNA, RNA and Protein abundance for proteins from chromosomes 5 and 26 and included in cluster 1. k Sheet in S7 Table. DNAxRNAxProt_chr5&26_cluster3. Correlation table between DNA, RNA and Protein abundance for proteins from chromosomes 5 and 26 and included in cluster 3. l Sheet in S7 Table. Category_chr5&26_cluster1. List of proteins present in cluster 1 manually assigned to a category.
(XLSX)

S8 Table snoRNA expression levels in EP.1 and LP.1 parasites. (DOCX)

S1 Code The file S1 Code represents the script designed to establish the list of *L*. *donovani* GO terms using available GO-identifiers from the corresponding orthologs in *L*. *donovani* strain LdBPK, *L*. *infantum*, *L*. *major*, *L*. *mexicana*, *Typanosoma brucei brucei 927* and *Typanosoma cruzi*.
(ZIP)

## References

[1] WHO. Leishmaniasis in high-burden countries: an epidemiological update based on data reported in 2014. 2016 Jun 3. Report No.: 0049–8114 (Print) 0049–8114 (Linking) Contract No.: 22. 27263128

[2] Lindgren E, Andersson Y, Suk JE, Sudre B, Semenza JC. Public health. Monitoring EU emerging infectious disease risk due to climate change. Science. 2012;336(6080):418–9. doi: 10.1126/science.1215735 22539705

[3] Koonin EV, Wolf YI. Evolution of microbes and viruses: a paradigm shift in evolutionary biology? Front Cell Infect Microbiol. 2012;2:119. doi: 10.3389/fcimb.2012.00119 22993722 PMC3440604

[4] Michaeli S. Trans-splicing in trypanosomes: machinery and its impact on the parasite transcriptome. Future Microbiol. 2011;6(4):459–74. doi: 10.2217/fmb.11.20 21526946

[5] Clayton CE. Gene expression in Kinetoplastids. Curr Opin Microbiol. 2016;32:46–51. doi: 10.1016/j.mib.2016.04.018 27177350

[6] Sterkers Y, Walton EL. The Leishmania chromosome lottery. Microbes Infect. 2014;16(1):2–5. doi: 10.1016/j.micinf.2013.11.008 24286926

[7] Rogers MB, Hilley JD, Dickens NJ, Wilkes J, Bates PA, Depledge DP, et al. Chromosome and gene copy number variation allow major structural change between species and strains of Leishmania. Genome Res. 2011;21(12):2129–42. doi: 10.1101/gr.122945.111 22038252 PMC3227102

[8] Dumetz F, Imamura H, Sanders M, Seblova V, Myskova J, Pescher P, et al. Modulation of Aneuploidy in Leishmania donovani during Adaptation to Different In Vitro and In Vivo Environments and Its Impact on Gene Expression. mBio. 2017;8(3). doi: 10.1128/mBio.00599-17 28536289 PMC5442457

[9] Iantorno SA, Durrant C, Khan A, Sanders MJ, Beverley SM, Warren WC, et al. Gene Expression in Leishmania Is Regulated Predominantly by Gene Dosage. mBio. 2017;8(5). doi: 10.1128/mBio.01393-17 28900023 PMC5596349

[10] Prieto Barja P, Pescher P, Bussotti G, Dumetz F, Imamura H, Kedra D, et al. Haplotype selection as an adaptive mechanism in the protozoan pathogen Leishmania donovani. Nat Ecol Evol. 2017;1(12):1961–9. doi: 10.1038/s41559-017-0361-x 29109466

[11] Brotherton M-C, Bourassa S, Leprohon P, Légaré D, Poirier GG, Droit A, et al. Proteomic and Genomic Analyses of Antimony Resistant Leishmania infantum Mutant. PLOS ONE. 2013;8(11):e81899. doi: 10.1371/journal.pone.0081899 24312377 PMC3842243

[12] Downing T, Imamura H, Decuypere S, Clark TG, Coombs GH, Cotton JA, et al. Whole genome sequencing of multiple Leishmania donovani clinical isolates provides insights into population structure and mechanisms of drug resistance. Genome Res. 2011;21(12):2143–56. doi: 10.1101/gr.123430.111 22038251 PMC3227103

[13] Laffitte MN, Leprohon P, Papadopoulou B, Ouellette M. Plasticity of the Leishmania genome leading to gene copy number variations and drug resistance. F1000Res. 2016;5:2350. doi: 10.12688/f1000research.9218.1 27703673 PMC5031125

[14] Leprohon P, Legare D, Raymond F, Madore E, Hardiman G, Corbeil J, et al. Gene expression modulation is associated with gene amplification, supernumerary chromosomes and chromosome loss in antimony-resistant Leishmania infantum. Nucleic Acids Res. 2009;37(5):1387–99. doi: 10.1093/nar/gkn1069 19129236 PMC2655676

[15] Ubeda JM, Raymond F, Mukherjee A, Plourde M, Gingras H, Roy G, et al. Genome-wide stochastic adaptive DNA amplification at direct and inverted DNA repeats in the parasite Leishmania. PLoS Biol. 2014;12(5):e1001868. doi: 10.1371/journal.pbio.1001868 24844805 PMC4028189

[16] Zhang WW, Ramasamy G, McCall LI, Haydock A, Ranasinghe S, Abeygunasekara P, et al. Genetic analysis of Leishmania donovani tropism using a naturally attenuated cutaneous strain. PLoS Pathog. 2014;10(7):e1004244. doi: 10.1371/journal.ppat.1004244 24992200 PMC4081786

[17] Bussotti G, Piel L., Pescher P., Domagalska M., Rajan K. S., Doniger T., Hiregange D., Myler P. J., Unger R., Michaeli S., and Spath G. F. Genome instability drives epistatic adaptation in the human pathogen Leishmania. bioRxiv, PNAS accepted for publication. 2021. doi: 10.1073/pnas.2113744118 34903666 PMC8713814

[18] Yona AH, Manor YS, Herbst RH, Romano GH, Mitchell A, Kupiec M, et al. Chromosomal duplication is a transient evolutionary solution to stress. Proceedings of the National Academy of Sciences of the United States of America. 2012;109(51):21010–5. doi: 10.1073/pnas.1211150109 23197825 PMC3529009

[19] Negrini S, Gorgoulis VG, Halazonetis TD. Genomic instability—an evolving hallmark of cancer. Nat Rev Mol Cell Biol. 2010;11(3):220–8. doi: 10.1038/nrm2858 20177397

[20] Bussotti G, Gouzelou E, Cortes Boite M, Kherachi I, Harrat Z, Eddaikra N, et al. Leishmania Genome Dynamics during Environmental Adaptation Reveal Strain-Specific Differences in Gene Copy Number Variation, Karyotype Instability, and Telomeric Amplification. mBio. 2018;9(6). 10.1128/mBio.01399-18 PMC6222132 30401775

[21] Melo GD, Goyard S, Lecoeur H, Rouault E, Pescher P, Fiette L, et al. New insights into experimental visceral leishmaniasis: Real-time in vivo imaging of Leishmania donovani virulence. PLoS Negl Trop Dis. 2017;11(9):e0005924. doi: 10.1371/journal.pntd.0005924 28945751 PMC5629011

[22] Bakin A, Ofengand J. Mapping of the 13 pseudouridine residues in Saccharomyces cerevisiae small subunit ribosomal RNA to nucleotide resolution. Nucleic Acids Res. 1995;23(16):3290–4. doi: 10.1093/nar/23.16.3290 7545286 PMC307190

[23] Chikne V, Doniger T, Rajan KS, Bartok O, Eliaz D, Cohen-Chalamish S, et al. A pseudouridylation switch in rRNA is implicated in ribosome function during the life cycle of Trypanosoma brucei. Sci Rep. 2016;6:25296. doi: 10.1038/srep25296 27142987 PMC4855143

[24] Rajan KS, Doniger T, Cohen-Chalamish S, Chen D, Semo O, Aryal S, et al. Pseudouridines on Trypanosoma brucei spliceosomal small nuclear RNAs and their implication for RNA and protein interactions. Nucleic Acids Res. 2019;47(14):7633–47. doi: 10.1093/nar/gkz477 31147702 PMC6698659

[25] Liang XH, Xu YX, Michaeli S. The spliced leader-associated RNA is a trypanosome-specific sn(o) RNA that has the potential to guide pseudouridine formation on the SL RNA. RNA. 2002;8(2):237–46. doi: 10.1017/s1355838202018290 11911368 PMC1370245

[26] Li H, Handsaker B, Wysoker A, Fennell T, Ruan J, Homer N, et al. The Sequence Alignment/Map format and SAMtools. Bioinformatics. 2009;25(16):2078–9. doi: 10.1093/bioinformatics/btp352 19505943 PMC2723002

[27] DePristo MA, Banks E, Poplin R, Garimella KV, Maguire JR, Hartl C, et al. A framework for variation discovery and genotyping using next-generation DNA sequencing data. Nat Genet. 2011;43(5):491–8. doi: 10.1038/ng.806 21478889 PMC3083463

[28] Quinlan AR, Hall IM. BEDTools: a flexible suite of utilities for comparing genomic features. Bioinformatics. 2010;26(6):841–2. doi: 10.1093/bioinformatics/btq033 20110278 PMC2832824

[29] Liao Y, Smyth GK, Shi W. featureCounts: an efficient general purpose program for assigning sequence reads to genomic features. Bioinformatics. 2014;30(7):923–30. doi: 10.1093/bioinformatics/btt656 24227677

[30] Cokelaer T, Desvillecharbrol D, Legendre R, Cardon M. Sequana’: a Set of Snakemake NGS pipelines. Journal of Open Source Software. 2017;2(16):352.

[31] Martin M. Cutadapt removes adapter sequences from high-throughput sequencing reads. 2011. 2011;17(1):3.

[32] Dobin A, Davis CA, Schlesinger F, Drenkow J, Zaleski C, Jha S, et al. STAR: ultrafast universal RNA-seq aligner. Bioinformatics. 2012;29(1):15–21. doi: 10.1093/bioinformatics/bts635 23104886 PMC3530905

[33] Team RC. R: A Language and Environment for Statistical Computing. R Foundation for Statistical Computing 2016.

[34] Love MI, Huber W, Anders S. Moderated estimation of fold change and dispersion for RNA-seq data with DESeq2. Genome Biol. 2014;15(12):550. doi: 10.1186/s13059-014-0550-8 25516281 PMC4302049

[35] Benjamini Y, Hochberg Y. Controlling the False Discovery Rate: A Practical and Powerful Approach to Multiple Testing. Journal of the Royal Statistical Society Series B (Methodological). 1995;57(1):289–300.

[36] Edgar R, Domrachev M, Lash AE. Gene Expression Omnibus: NCBI gene expression and hybridization array data repository. Nucleic Acids Res. 2002;30(1):207–10. doi: 10.1093/nar/30.1.207 11752295 PMC99122

[37] Cox J, Mann M. MaxQuant enables high peptide identification rates, individualized p.p.b.-range mass accuracies and proteome-wide protein quantification. Nat Biotechnol. 2008;26(12):1367–72. doi: 10.1038/nbt.1511 19029910

[38] Cox J, Neuhauser N, Michalski A, Scheltema RA, Olsen JV, Mann M. Andromeda: a peptide search engine integrated into the MaxQuant environment. J Proteome Res. 2011;10(4):1794–805. doi: 10.1021/pr101065j 21254760

[39] Cox J, Hein MY, Luber CA, Paron I, Nagaraj N, Mann M. Accurate proteome-wide label-free quantification by delayed normalization and maximal peptide ratio extraction, termed MaxLFQ. Mol Cell Proteomics. 2014;13(9):2513–26. doi: 10.1074/mcp.M113.031591 24942700 PMC4159666

[40] Perez-Riverol Y, Csordas A, Bai J, Bernal-Llinares M, Hewapathirana S, Kundu DJ, et al. The PRIDE database and related tools and resources in 2019: improving support for quantification data. Nucleic Acids Res. 2019;47(D1):D442–D50. doi: 10.1093/nar/gky1106 30395289 PMC6323896

[41] Wieczorek S, Combes F, Lazar C, Giai Gianetto Q, Gatto L, Dorffer A, et al. DAPAR & ProStaR: software to perform statistical analyses in quantitative discovery proteomics. Bioinformatics. 2017;33(1):135–6. doi: 10.1093/bioinformatics/btw580 27605098 PMC5408771

[42] Smyth GK. limma: Linear Models for Microarray Data. In: Gentleman R, Carey VJ, Huber W, Irizarry RA, Dudoit S, editors. Bioinformatics and Computational Biology Solutions Using R and Bioconductor. New York, NY: Springer New York; 2005. p. 397–420.

[43] Ritchie ME, Phipson B, Wu D, Hu Y, Law CW, Shi W, et al. limma powers differential expression analyses for RNA-sequencing and microarray studies. Nucleic Acids Res. 2015;43(7):e47. doi: 10.1093/nar/gkv007 25605792 PMC4402510

[44] Giai Gianetto Q, Combes F, Ramus C, Bruley C, Coute Y, Burger T. Calibration plot for proteomics: A graphical tool to visually check the assumptions underlying FDR control in quantitative experiments. Proteomics. 2016;16(1):29–32. doi: 10.1002/pmic.201500189 26572953

[45] Pounds S, Cheng C. Robust estimation of the false discovery rate. Bioinformatics. 2006;22(16):1979–87. doi: 10.1093/bioinformatics/btl328 16777905

[46] Aulner N, Danckaert A, Rouault-Hardoin E, Desrivot J, Helynck O, Commere PH, et al. High content analysis of primary macrophages hosting proliferating Leishmania amastigotes: application to anti-leishmanial drug discovery. PLoS Negl Trop Dis. 2013;7(4):e2154. doi: 10.1371/journal.pntd.0002154 23593521 PMC3617141

[47] Volf P, Volfova V. Establishment and maintenance of sand fly colonies. J Vector Ecol. 2011;36 Suppl 1:S1–9. doi: 10.1111/j.1948-7134.2011.00106.x 21366760

[48] Seblova V, Volfova V, Dvorak V, Pruzinova K, Votypka J, Kassahun A, et al. Phlebotomus orientalis sand flies from two geographically distant Ethiopian localities: biology, genetic analyses and susceptibility to Leishmania donovani. PLoS Negl Trop Dis. 2013;7(4):e2187. doi: 10.1371/journal.pntd.0002187 23638207 PMC3636102

[49] Sadlova J, Price HP, Smith BA, Votypka J, Volf P, Smith DF. The stage-regulated HASPB and SHERP proteins are essential for differentiation of the protozoan parasite Leishmania major in its sand fly vector, Phlebotomus papatasi. Cell Microbiol. 2010;12(12):1765–79. doi: 10.1111/j.1462-5822.2010.01507.x 20636473 PMC3015063

[50] Pescher P, Blisnick T, Bastin P, Spath GF. Quantitative proteome profiling informs on phenotypic traits that adapt Leishmania donovani for axenic and intracellular proliferation. Cell Microbiol. 2011;13(7):978–91. doi: 10.1111/j.1462-5822.2011.01593.x 21501362

[51] Spath GF, Beverley SM. A lipophosphoglycan-independent method for isolation of infective Leishmania metacyclic promastigotes by density gradient centrifugation. Exp Parasitol. 2001;99(2):97–103. doi: 10.1006/expr.2001.4656 11748963

[52] Rogers ME, Chance ML, Bates PA. The role of promastigote secretory gel in the origin and transmission of the infective stage of Leishmania mexicana by the sandfly Lutzomyia longipalpis. Parasitology. 2002;124(Pt 5):495–507. doi: 10.1017/s0031182002001439 12049412

[53] Lei SM, Romine NM, Beetham JK. Population changes in Leishmania chagasi promastigote developmental stages due to serial passage. J Parasitol. 2010;96(6):1134–8. doi: 10.1645/GE-2566.1 21158623 PMC3627396

[54] McGwire B, Chang KP. Genetic rescue of surface metalloproteinase (gp63)-deficiency in Leishmania amazonensis variants increases their infection of macrophages at the early phase. Mol Biochem Parasitol. 1994;66(2):345–7. doi: 10.1016/0166-6851(94)90160-0 7808483

[55] Spath GF, Epstein L, Leader B, Singer SM, Avila HA, Turco SJ, et al. Lipophosphoglycan is a virulence factor distinct from related glycoconjugates in the protozoan parasite Leishmania major. Proceedings of the National Academy of Sciences of the United States of America. 2000;97(16):9258–63. doi: 10.1073/pnas.160257897 10908670 PMC16855

[56] Erben ED, Fadda A, Lueong S, Hoheisel JD, Clayton C. A genome-wide tethering screen reveals novel potential post-transcriptional regulators in Trypanosoma brucei. PLoS Pathog. 2014;10(6):e1004178. doi: 10.1371/journal.ppat.1004178 24945722 PMC4055773

[57] Lueong S, Merce C, Fischer B, Hoheisel JD, Erben ED. Gene expression regulatory networks in Trypanosoma brucei: insights into the role of the mRNA-binding proteome. Mol Microbiol. 2016;100(3):457–71. doi: 10.1111/mmi.13328 26784394

[58] de Pablos LM, Ferreira TR, Dowle AA, Forrester S, Parry E, Newling K, et al. The mRNA-bound Proteome of Leishmania mexicana: Novel Genetic Insight into an Ancient Parasite. Mol Cell Proteomics. 2019;18(7):1271–84. doi: 10.1074/mcp.RA118.001307 30948621 PMC6601212

[59] Rajan KS, Chikne V, Decker K, Waldman Ben-Asher H, Michaeli S. Unique Aspects of rRNA Biogenesis in Trypanosomatids. Trends Parasitol. 2019;35(10):778–94. doi: 10.1016/j.pt.2019.07.012 31473096

[60] Kamina AD, Williams N. Ribosome Assembly in Trypanosomatids: A Novel Therapeutic Target. Trends Parasitol. 2017;33(4):256–7. doi: 10.1016/j.pt.2016.12.003 27988096 PMC5376504

[61] Watkins NJ, Bohnsack MT. The box C/D and H/ACA snoRNPs: key players in the modification, processing and the dynamic folding of ribosomal RNA. Wiley Interdiscip Rev RNA. 2012;3(3):397–414. doi: 10.1002/wrna.117 22065625

[62] Eliaz D, Doniger T, Tkacz ID, Biswas VK, Gupta SK, Kolev NG, et al. Genome-wide analysis of small nucleolar RNAs of Leishmania major reveals a rich repertoire of RNAs involved in modification and processing of rRNA. RNA Biol. 2015;12(11):1222–55. doi: 10.1080/15476286.2015.1038019 25970223 PMC4829279

[63] Lenski RE. What is adaptation by natural selection? Perspectives of an experimental microbiologist. PLoS Genet. 2017;13(4):e1006668. doi: 10.1371/journal.pgen.1006668 28426692 PMC5398481

[64] Clayton C, Shapira M. Post-transcriptional regulation of gene expression in trypanosomes and leishmanias. Mol Biochem Parasitol. 2007;156(2):93–101. doi: 10.1016/j.molbiopara.2007.07.007 17765983

[65] Tsai HJ, Nelliat A. A Double-Edged Sword: Aneuploidy is a Prevalent Strategy in Fungal Adaptation. Genes (Basel). 2019;10(10). doi: 10.3390/genes10100787 31658789 PMC6826469

[66] Haile S, Papadopoulou B. Developmental regulation of gene expression in trypanosomatid parasitic protozoa. Curr Opin Microbiol. 2007;10(6):569–77. doi: 10.1016/j.mib.2007.10.001 18177626

[67] Goldstrohm AC, Hall TMT, McKenney KM. Post-transcriptional Regulatory Functions of Mammalian Pumilio Proteins. Trends Genet. 2018;34(12):972–90. doi: 10.1016/j.tig.2018.09.006 30316580 PMC6251728

[68] Thomas G. An encore for ribosome biogenesis in the control of cell proliferation. Nat Cell Biol. 2000;2(5):E71–2. doi: 10.1038/35010581 10806485

[69] McCutchan TF, de la Cruz VF, Good MF, Wellems TE. Antigenic diversity in Plasmodium falciparum. Prog Allergy. 1988;41:173–92. doi: 10.1159/000415223 2457215

[70] Li J, Gutell RR, Damberger SH, Wirtz RA, Kissinger JC, Rogers MJ, et al. Regulation and trafficking of three distinct 18 S ribosomal RNAs during development of the malaria parasite. J Mol Biol. 1997;269(2):203–13. doi: 10.1006/jmbi.1997.1038 9191065

[71] Guo H. Specialized ribosomes and the control of translation. Biochem Soc Trans. 2018;46(4):855–69. doi: 10.1042/BST20160426 29986937

[72] Ramagopal S, Ennis HL. Regulation of synthesis of cell-specific ribosomal proteins during differentiation of Dictyostelium discoideum. Proceedings of the National Academy of Sciences of the United States of America. 1981;78(5):3083–7. doi: 10.1073/pnas.78.5.3083 16593020 PMC319504

[73] Ramagopal S. Induction of cell-specific ribosomal proteins in aggregation-competent nonmorphogenetic Dictyostelium discoideum. Biochem Cell Biol. 1990;68(11):1281–7. doi: 10.1139/o90-190 2275804

[74] Locati MD, Pagano JFB, Girard G, Ensink WA, van Olst M, van Leeuwen S, et al. Expression of distinct maternal and somatic 5.8S, 18S, and 28S rRNA types during zebrafish development. RNA. 2017;23(8):1188–99. doi: 10.1261/rna.061515.117 28500251 PMC5513064

[75] Belin S, Beghin A, Solano-Gonzalez E, Bezin L, Brunet-Manquat S, Textoris J, et al. Dysregulation of ribosome biogenesis and translational capacity is associated with tumor progression of human breast cancer cells. PLoS One. 2009;4(9):e7147. doi: 10.1371/journal.pone.0007147 19779612 PMC2744998

[76] Polacek N, Mankin AS. The ribosomal peptidyl transferase center: structure, function, evolution, inhibition. Crit Rev Biochem Mol Biol. 2005;40(5):285–311. doi: 10.1080/10409230500326334 16257828

[77] Jack K, Bellodi C, Landry DM, Niederer RO, Meskauskas A, Musalgaonkar S, et al. rRNA pseudouridylation defects affect ribosomal ligand binding and translational fidelity from yeast to human cells. Mol Cell. 2011;44(4):660–6. doi: 10.1016/j.molcel.2011.09.017 22099312 PMC3222873

[78] King TH, Liu B, McCully RR, Fournier MJ. Ribosome structure and activity are altered in cells lacking snoRNPs that form pseudouridines in the peptidyl transferase center. Mol Cell. 2003;11(2):425–35. doi: 10.1016/s1097-2765(03)00040-6 12620230

[79] Meleppattu S, Kamus-Elimeleh D, Zinoviev A, Cohen-Mor S, Orr I, Shapira M. The eIF3 complex of Leishmania-subunit composition and mode of recruitment to different cap-binding complexes. Nucleic Acids Res. 2015;43(13):6222–35. doi: 10.1093/nar/gkv564 26092695 PMC4513851

[80] Yoffe Y, Leger M, Zinoviev A, Zuberek J, Darzynkiewicz E, Wagner G, et al. Evolutionary changes in the Leishmania eIF4F complex involve variations in the eIF4E-eIF4G interactions. Nucleic Acids Res. 2009;37(10):3243–53. doi: 10.1093/nar/gkp190 19321500 PMC2691829

[81] Yoffe Y, Zuberek J, Lerer A, Lewdorowicz M, Stepinski J, Altmann M, et al. Binding specificities and potential roles of isoforms of eukaryotic initiation factor 4E in Leishmania. Eukaryot Cell. 2006;5(12):1969–79. doi: 10.1128/EC.00230-06 17041189 PMC1694823

