# Supplementary figures and images for: Experimental evolution links post-transcriptional regulation to Leishmania fitness gain
Source: PLoS Pathog. 2022 Mar 16;18(3):e1010375. doi: 10.1371/journal.ppat.1010375 (PMC8959184; doi:10.1371/journal.ppat.1010375)

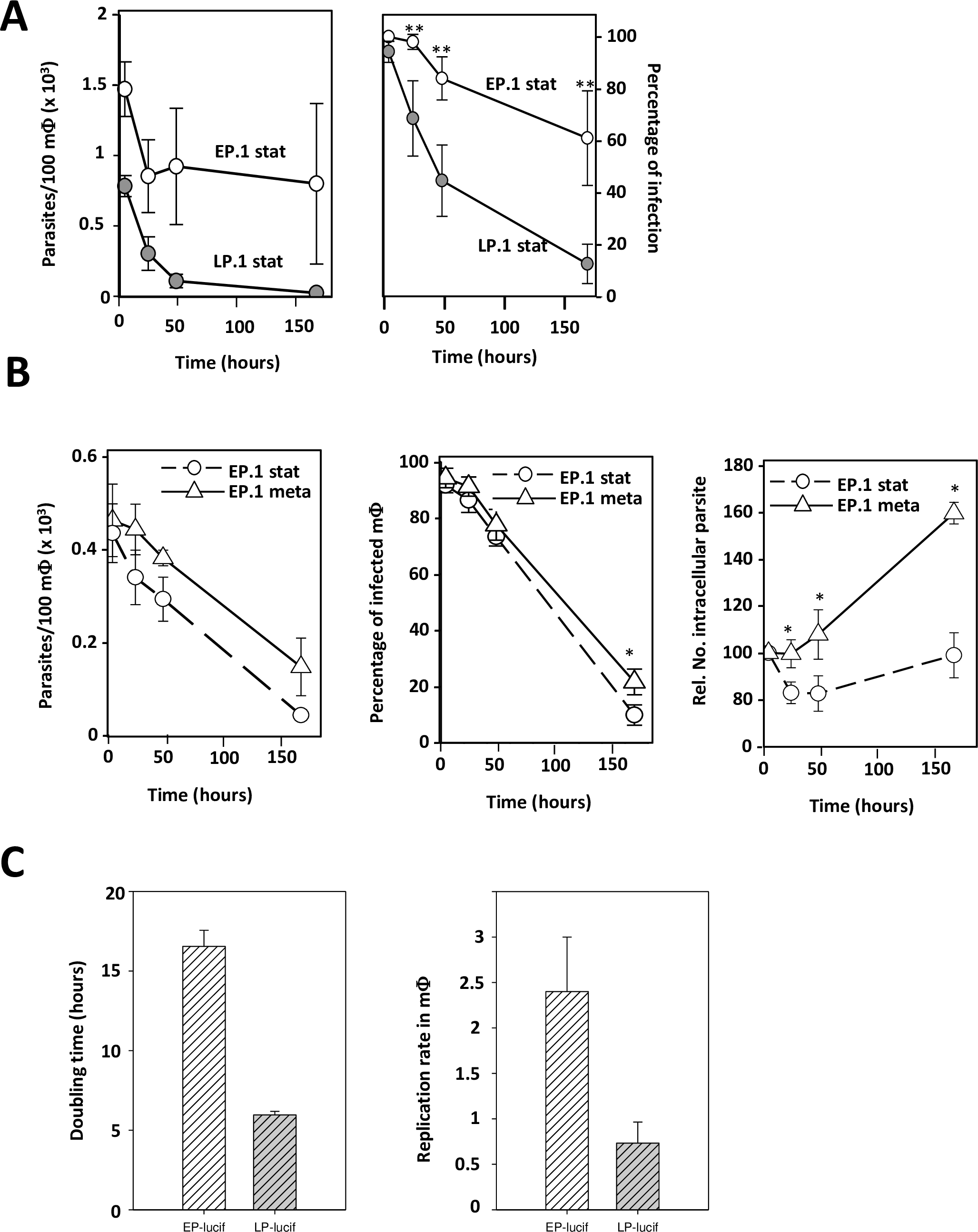

Supplement: S1 Fig — (A) Comparison of EP.1 stat (open circle) and LP.1 stat (grey circles) infectivity. Mean number of parasites per 100 macrophages +/-SD (left panel) and the percentage of infection (right panel) from three biological replicates are shown. (B) Comparison of EP.1 stat (open circle) and EP.1 meta (open triangle) infectivity. Mean number +/-SD of parasites per 100 macrophages (left panel), the percentage of infection (middle panel) and the relative number of intracellular parasites (right panel) from a representative experiment out of three replicates are shown. * indicates p-value ≤ 0.05. (C) Histogram plots representing the generation time of EP and LP parasites originated from an independent evolutionary experiment with parasites expressing luciferase (EP.luc and LP.luc) (left panel). Replication rate in infected macrophages for the EP.luc and LP.luc parasites calculated between day 1 and day 6 after infection (right panel). (TIF) [file ppat.1010375.s001.tif]

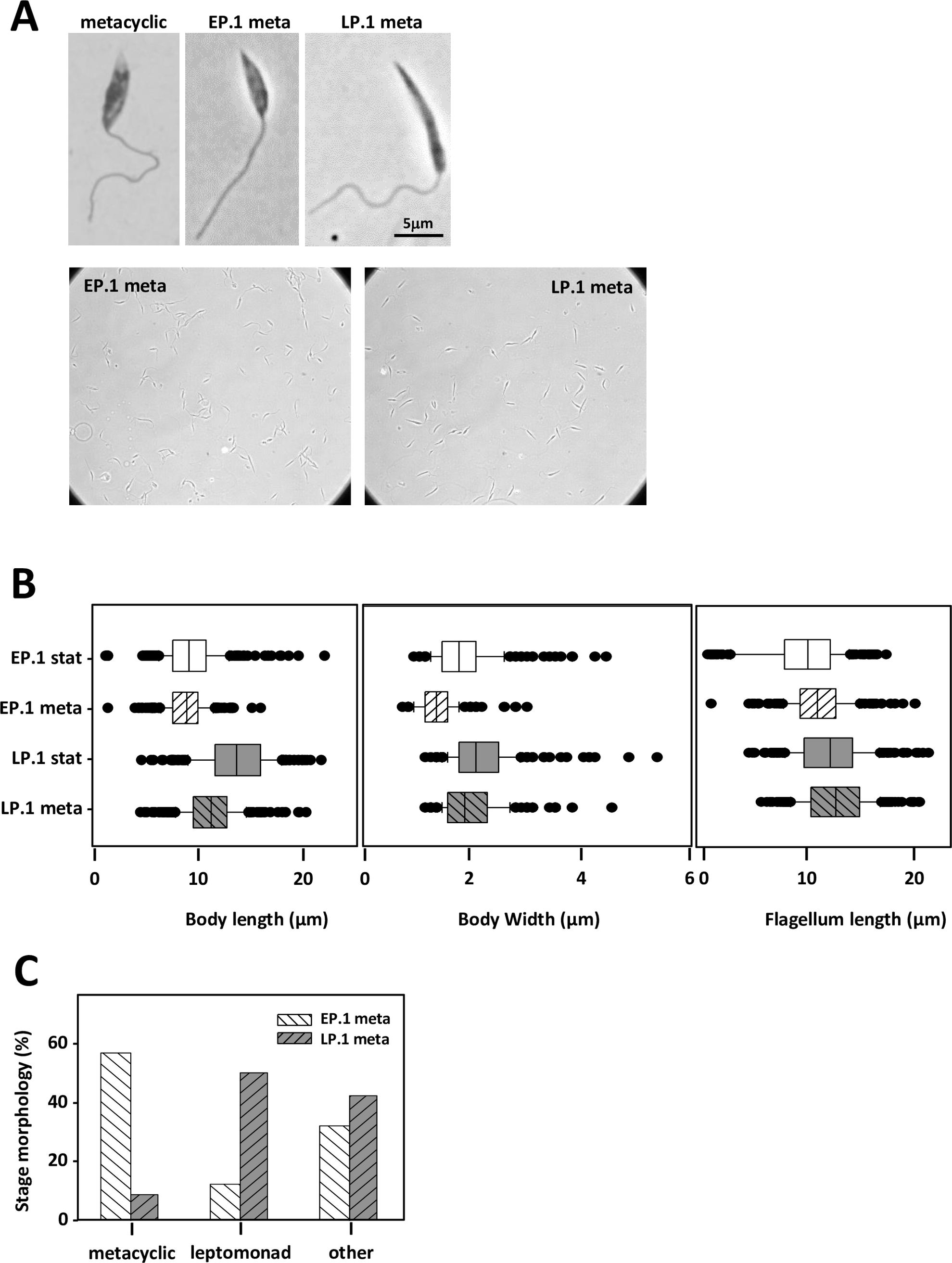

Supplement: S2 Fig — (A) Micrographs of representative EP.1 metacyclic isolated from the sand fly thoracic part (upper left image), Ficoll-enriched EP.1 (upper middle image) and LP.1 metacyclic-like parasites (upper right image) from stationary culture. Broad field images of EP.1 and LP.1 metacyclic enriched parasites are presented in the lower right and left images. (B) Quantitative morphological analysis of stationary-phase and metacyclic-enriched parasite populations. The box plots show the median values and the upper and lower quartiles for body length (left panel), body width (middle panel) and flagellum length (right panel). (C) Distribution of the indicated promastigote forms in EP.1 and LP.1 Ficoll-enriched metacyclic fractions. (TIF) [file ppat.1010375.s002.tif]

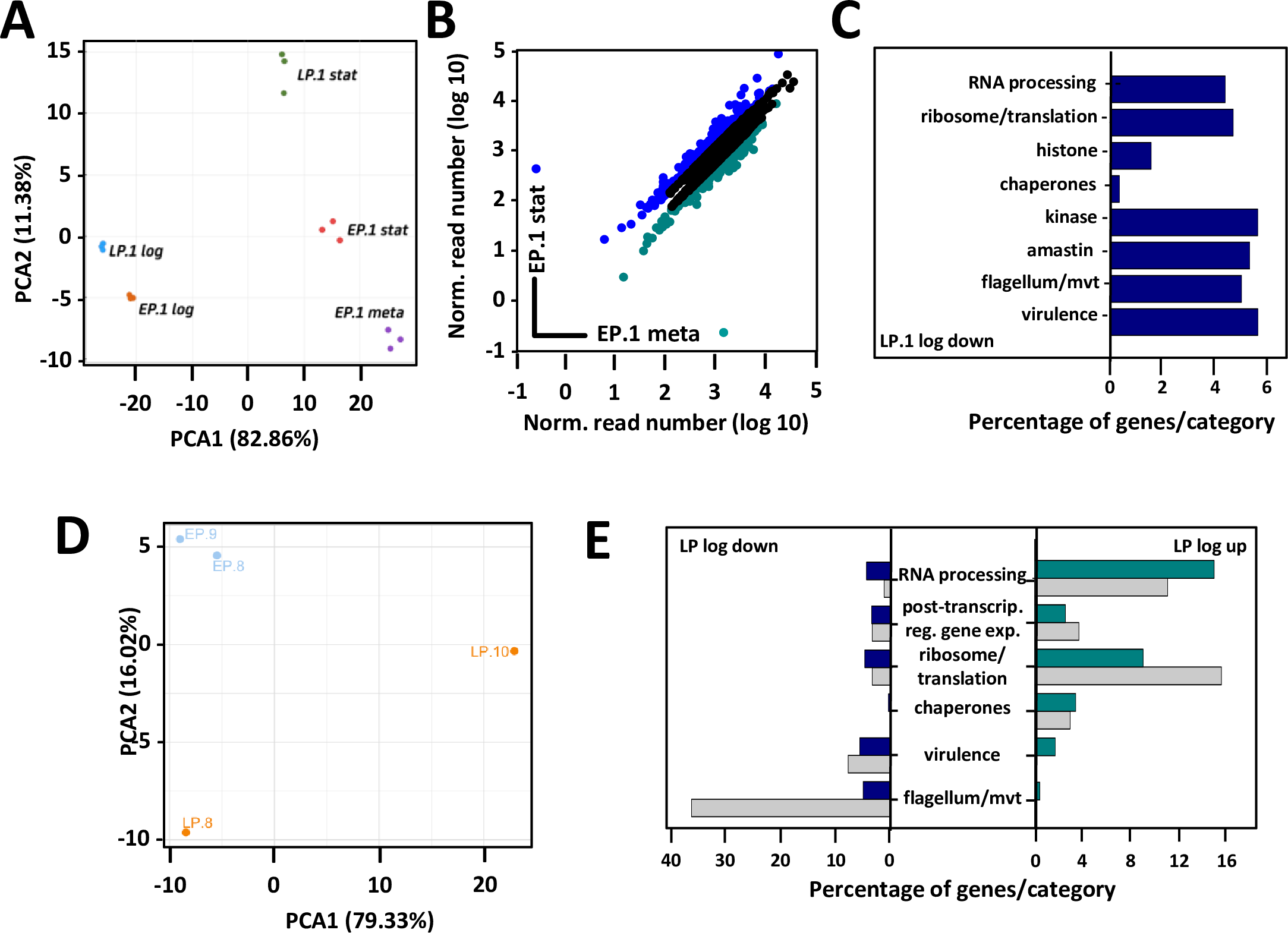

Supplement: S3 Fig — (A) Principal Component Analysis of EP.1 and LP.1 parasites from logarithmic (log) and stationary (stat) phase cultures, and after metacyclic enrichment (meta). (B) Ratio plots of normalized RNA abundance for EP.1 stat compared to EP.1 meta. Dark blue and dark cyan dots represent respectively gene expression changes with FC > 1.5 and adjusted p-value ≤ 0.01; black dots correspond to gene expression changes with adjusted p-value > 0.01. (C) Histogram plot representing the number of genes showing decreased transcript abundance in LP.1 log for the indicated gene categories (see Sheet j in S2 Table). (D) Principal Component Analysis of RNAseq for two independent EP and LP biological replicates from logarithmic phase culture. Samples are identified according to S9 Fig. (E) Histogram plot showing the number of genes with decreased and increased transcript abundance in LP log parasites from two independent transcriptomic analyses. Cyan and blue histogram bars represent the evolutionary experiment presented in Fig 2 (EP.1 and LP.1), grey bars correspond to the second RNAseq data set corresponding to EP.8, EP.9, LP.9 and LP.10 samples (see S9 Fig and Sheets c and e in S3 Table for detail). (TIF) [file ppat.1010375.s003.tif]

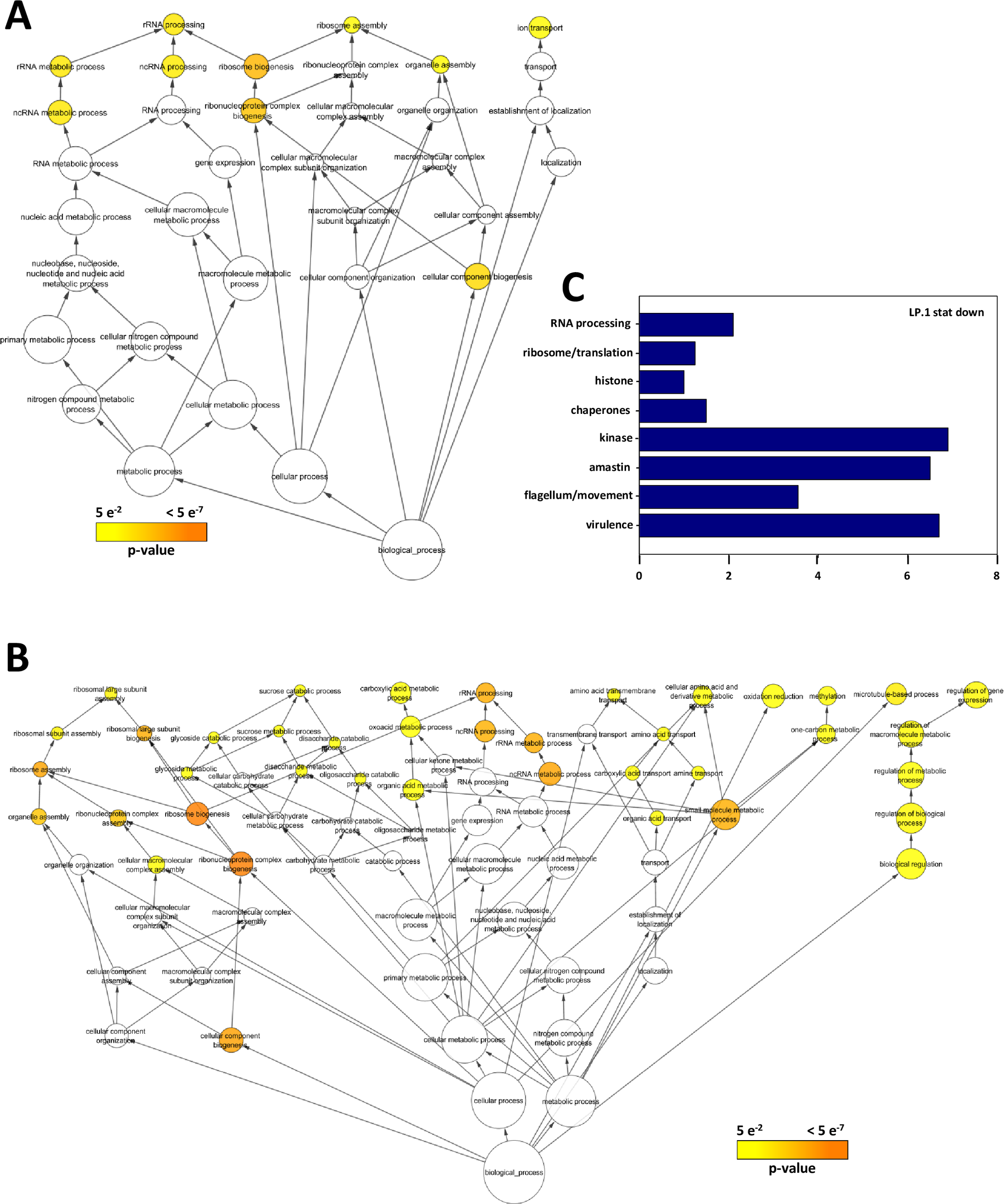

Supplement: S4 Fig — (A, B) Graphical representations generated with the BiNGO plugin of the Cytoscape software package for the GO term-enrichment analysis performed with the transcripts showing statistically significant increased abundance in LP.1 log (A) and LP.1 stat (B) (see Sheet o in S2 Table). The size of the circle is indicative of the number of genes falling in each category and the color ranging from yellow to orange indicates the p-values associated as indicated in the legend. (C) Histogram plot representing the number of genes showing decreased transcript abundance in LP.1 stat for the indicated gene categories (see Sheet n in S2 Table). (TIF) [file ppat.1010375.s004.tif]

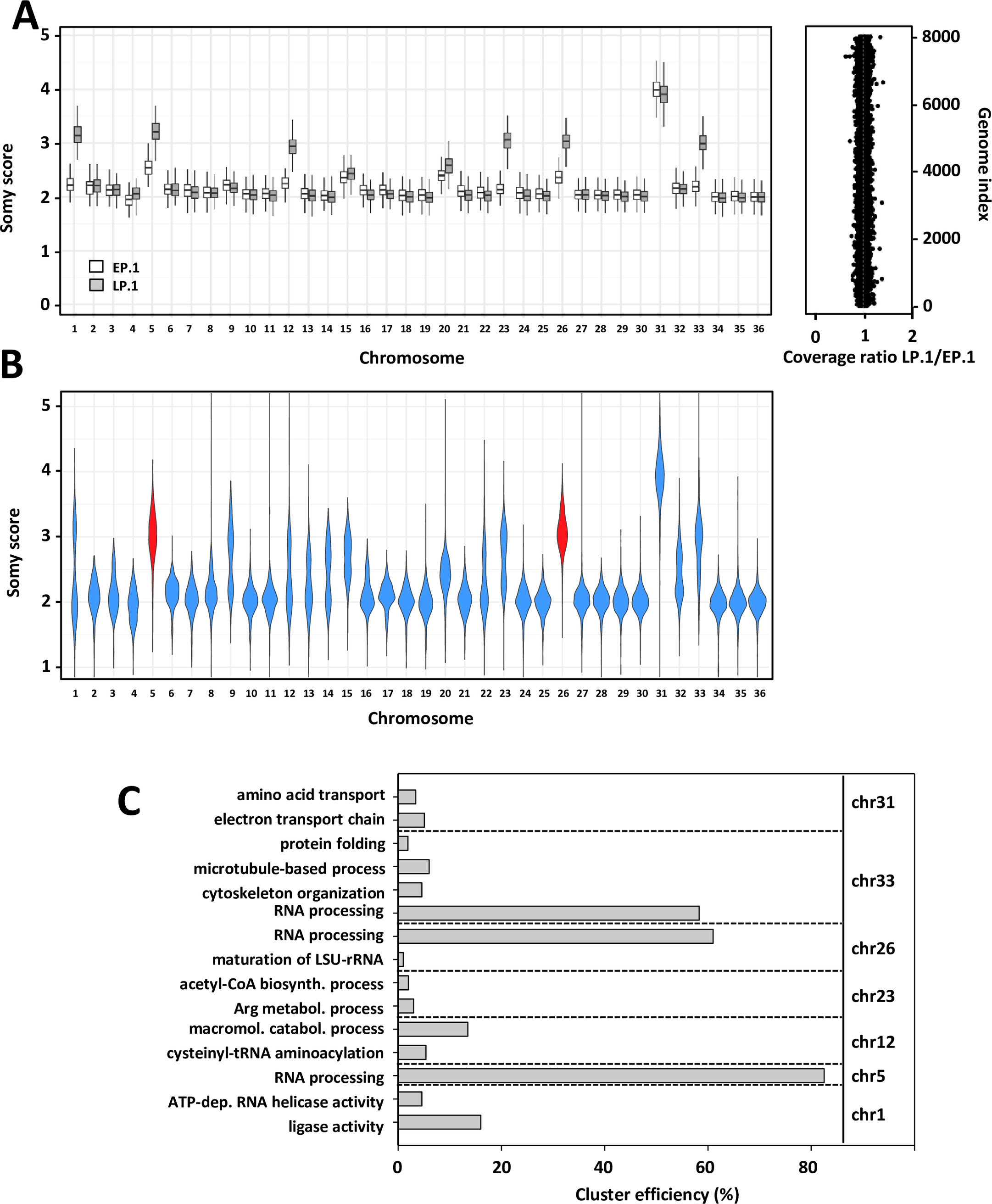

Supplement: S5 Fig — (A) Chromosome somy levels of EP.1 and LP.1 promastigotes. Chromosome read-depth distributions are shown in boxplots depicting the median and the upper and lower quartiles (left panel). Genome-wide coverage ratios (y axes) between LP.1 and EP.1 (right panel). Genome-wide coverage ratio (x- axis) between EP.1 and LP.1. The y axis reports the position of the genomic windows along the chromosomes. Dots represent genomic windows of 300 bases. (B) Violin plot computed from three independent evolutionary experiments representing the somy score distribution for each chromosome. In red are highlighted chr 5 and 26 that are trisomic in all three experiments (LP.1, LP.6 and LP.7). (C) Enrichment analysis of the aneuploid chromosomes for the GO categories ‘molecular function’ (chr 1) and ’biological process’ (5, 12, 23, 26, 31 and 33). The bars correspond to the cluster efficiency computed from GO term-enrichment analyses (see Sheet d in S4 Table). (TIF) [file ppat.1010375.s005.tif]

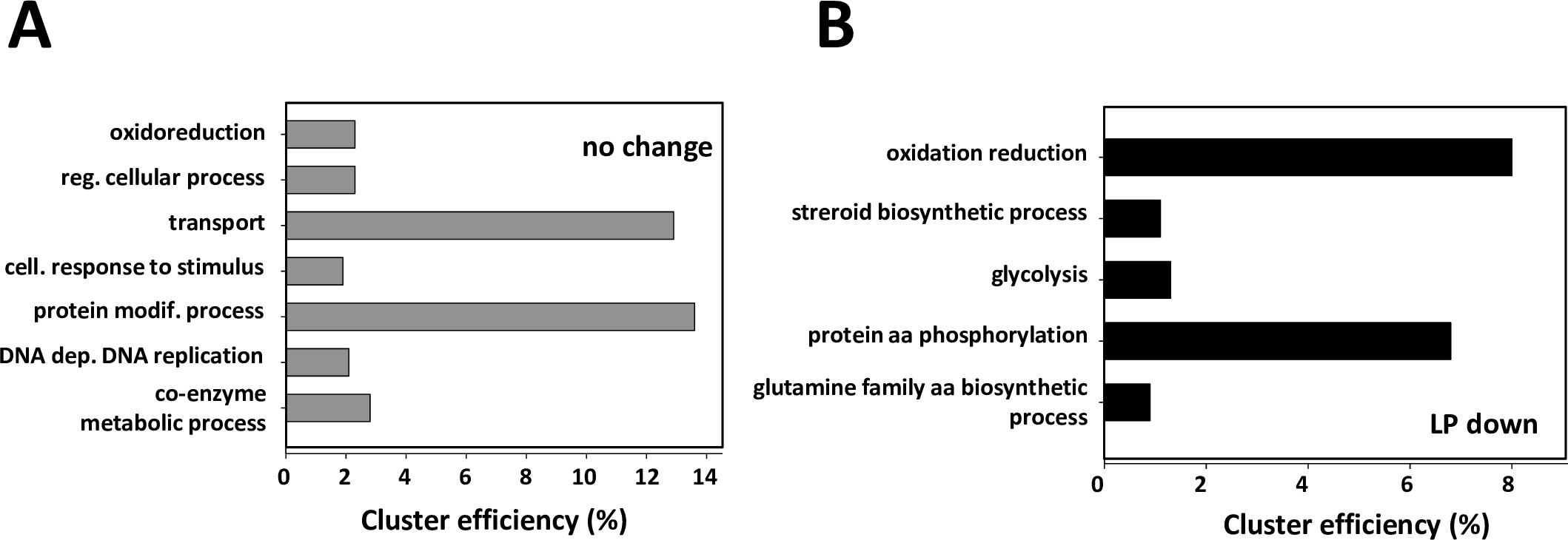

Supplement: S6 Fig — (A, B) Enrichment analysis for the GO category ’biological process’. RNA read counts were first normalized by DNA read counts to estimate the ratio of normalized RNA abundance between LP.1 and EP.1 (see Sheet i in S5 Table). (A) Histogram showing the cluster efficiency for 1,104 genes that show dosage dependent changes in mRNA abundance (ratio from 0.8 to 1.2), including 463 genes that are annotated with a GO term. (B) Histogram showing the cluster efficiency for 1,192 genes that show dosage in-dependent changes in mRNA abundance (ratio < 0.8), including 510 genes that are annotated with a GO term and show a decrease in RNA read counts after normalization to DNA read counts in LP.1 log parasites. (TIF) [file ppat.1010375.s006.tif]

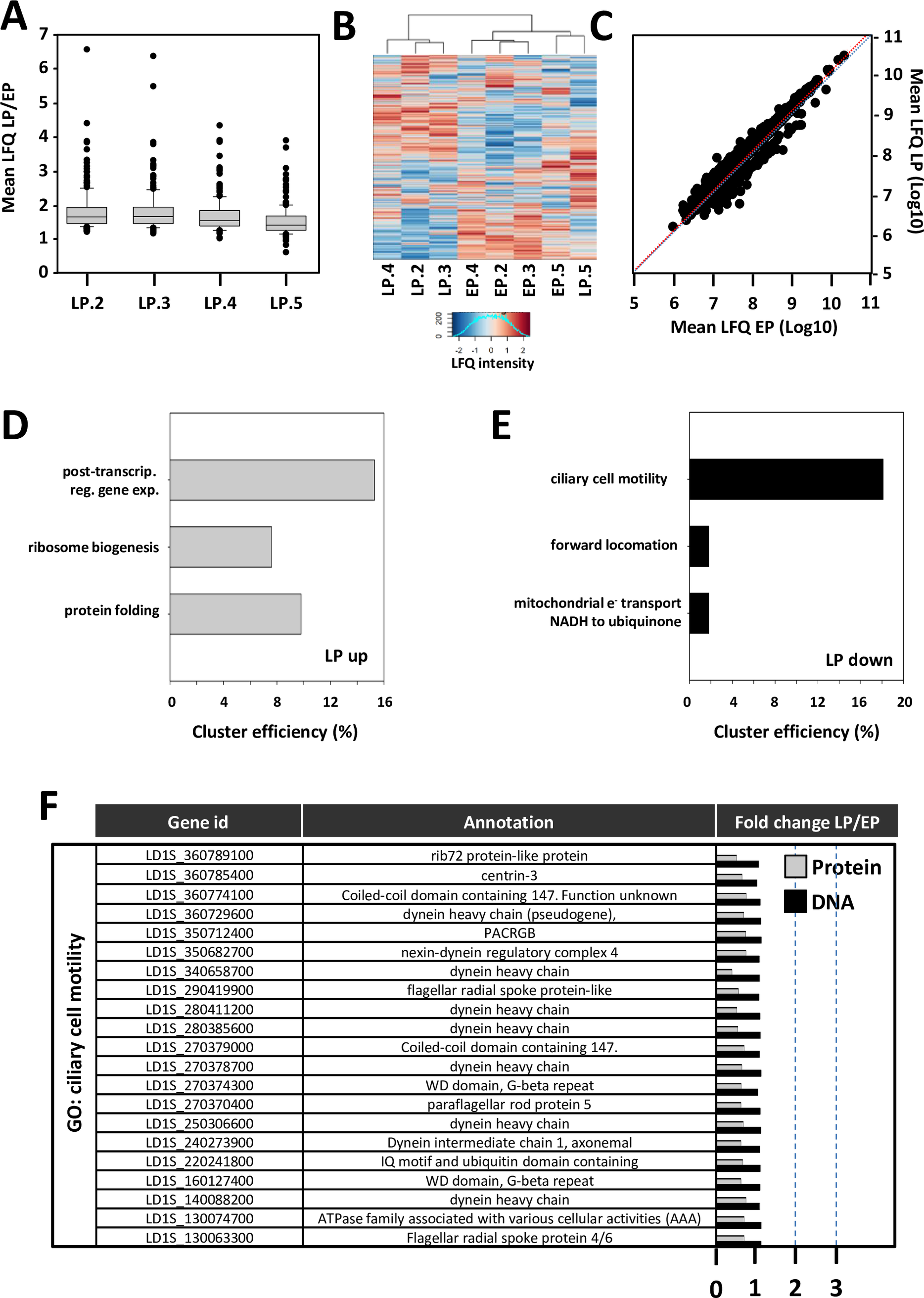

Supplement: S7 Fig — (A) Box plots representing the median ratio and the upper and lower quartiles of the LFQ intensity values for all LP biological replicates (LP.2, LP.3, LP.4 and LP.5) compared to the median of all EP replicates (EP.2, EP.3, EP.4 and EP.5). (B) Cluster analysis of all EP and LP samples (Ward method). (C) Ratio plot representing the mean LFQ intensity value between EP and LP for each individual, quantified protein. The experimental and the expected regression lines are shown in red and blue respectively. (D, E) Cluster efficiency for the GO category ‘biological process’ for proteins from cluster 1 whose abundance correlates with increased (D) or decreased normalized DNA read counts (E) in LP log parasites. Only proteins quantified with a p-value < 0.01 were considered for the GO term enrichment analysis (see Sheets e and g in S7 Table). (F) Table listing selected genes associated with the GO term ‘ciliary cell motility’ from the GO enrichment analysis presented in Fig 4E. Their respective fold change values computed from Protein LFQ intensities (grey bars) and DNA normalized read counts (black bars) for LP versus EP log parasites are represented. (TIF) [file ppat.1010375.s007.tif]

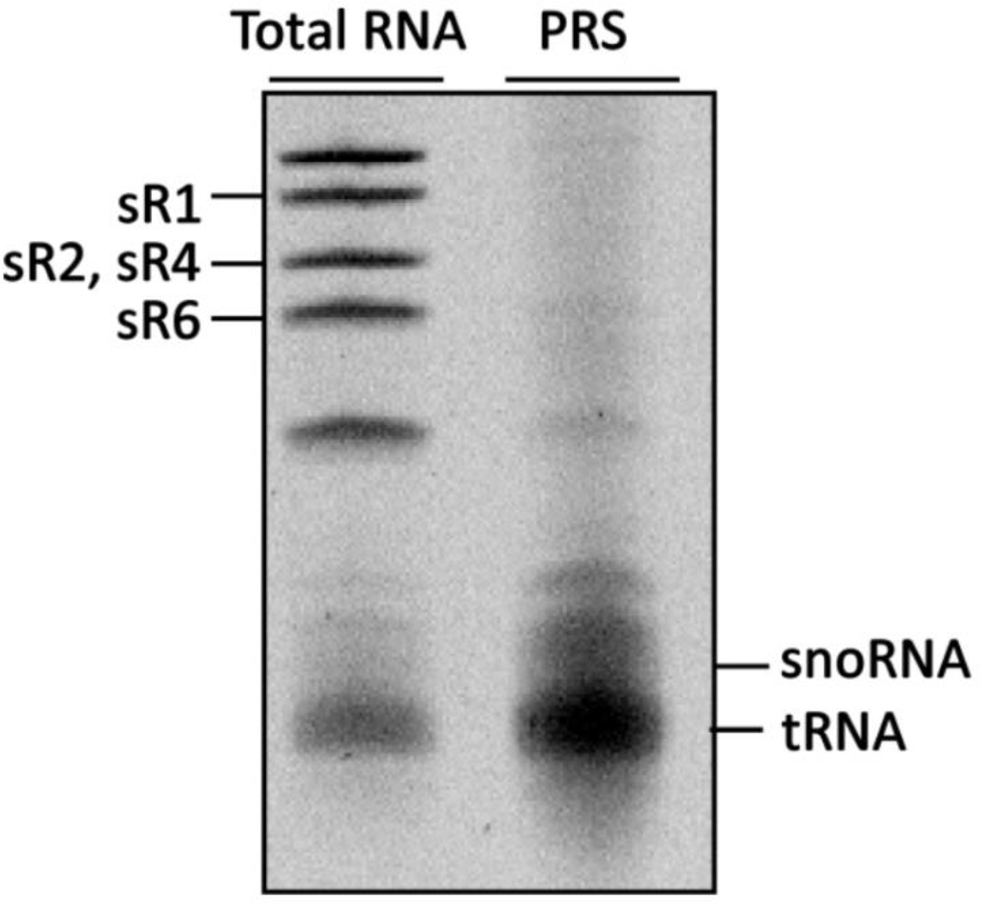

Supplement: S8 Fig — 2x10^9^ cells were disrupted by nitrogen cavitation under low salt concentration (150 mM KCl in the presence of high MgCl2 (10 mM) followed by ribosome extraction using high KCl (300 mM). The ribosomes were removed by centrifugation at 35,000 rpm for 2h. Two μg of RNA from total lysate (Total RNA) and PRS sample were separated on a 10% polyacrylamide gel and stained with ethidium bromide. (TIF) [file ppat.1010375.s008.tif]

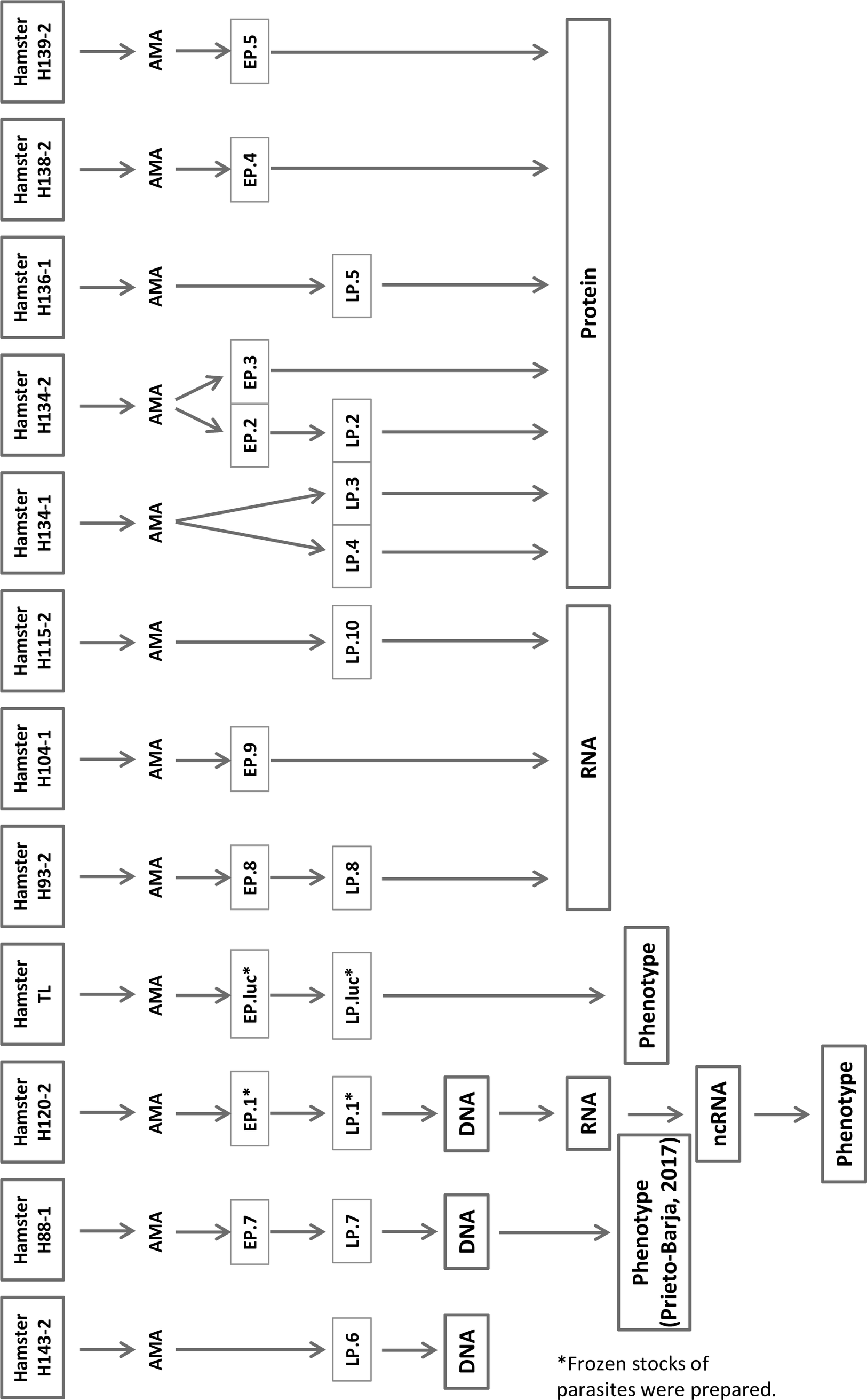

Supplement: S9 Fig — Each hamster infected with L. donovani parasites was identified by the cage number and is the source of amastigotes (AMA) for conversion to promastigotes. Early passage promastigotes (EP) and late passage promastigotes (LP) used for the genomic (DNA), transcriptomic (RNA), small RNome and transcriptome-wide mapping of pseudouridine sites (RNome & Ψ-seq), proteomic (Protein) and phenotypic analyses are identified. The parasites marked by an asterisk (*) were frozen at passage 2 and passage 20. (TIF) [file ppat.1010375.s009.tif]

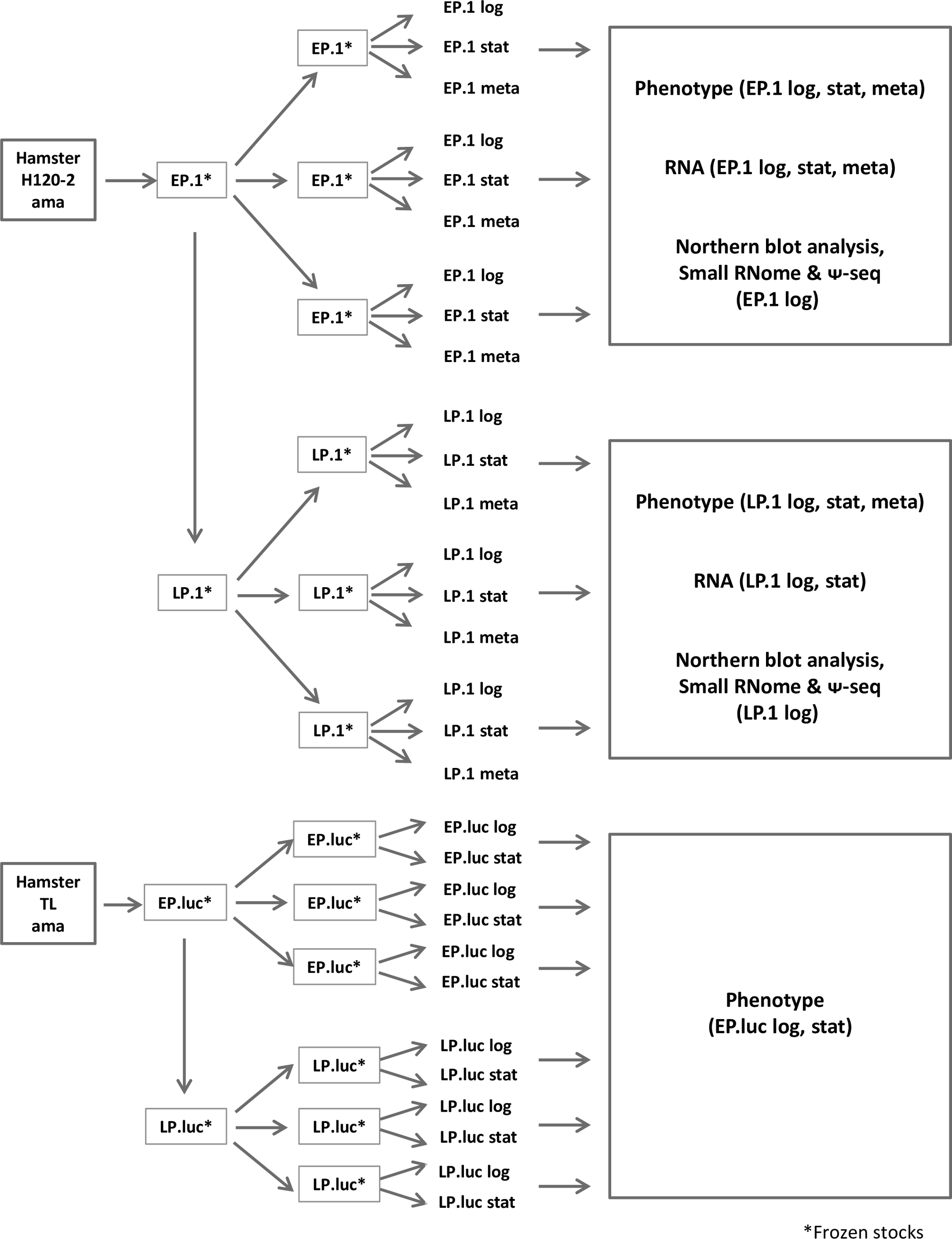

Supplement: S10 Fig — Strains issued from independent experimental evolution assays are identified by number (i.e. EP.1 and LP.1 are the strains resulting from experiment 1). Frozen stocks of EP.1, LP.1, EP.luc and LP.luc were prepared. The stage-specific expression analysis was therefore performed starting from three frozen aliquots prepared at passage 2 (EP.1) and passage 20 (LP.1). Each of the frozen parasites was used to prepare RNA extracts from log and stationary growth culture and from enriched metacyclic forms. Likewise, phenotypic analyses performed with EP.1, LP.1, EP.luc and LP.luc started from frozen aliquots for each replicate. (TIF) [file ppat.1010375.s010.tif]
