# Supplementary material for: Experimental evolution links post-transcriptional regulation to Leishmania fitness gain
Source: PLoS Pathog. 2022 Mar 16;18(3):e1010375. doi: 10.1371/journal.ppat.1010375 (PMC8959184; doi:10.1371/journal.ppat.1010375)
Supplement: Attachment — Submitted filename: full-revision RC-2021-00937.pdf [file ppat.1010375.s020.pdf]

Full Revision

Manuscript number: RC-2021-00937
Corresponding author(s): Gerald, Späth

1. General Statements [optional]

Dear Editors,

We are pleased to submit a fully revised version of our manuscript entitled 'Experimental evolution reveals post-transcriptional regulation as a novel driver of *Leishmania* fitness gain', which has generated considerable enthusiasm by the reviewers as judged by comments such as *"The data on the small RNAs and on the ribosomal modifications are highly interesting and will pave the way towards analysing the presence and function of fitness adapted ribosomes in* Leishmania*"*, or *"The paper is of great importance to researchers working on* Leishmania *and trypanosomes, but could have implications on research on other fields as well"*.

A hallmark of the human parasite *Leishmania* is its intrinsic genome instability. In a recent series of publications, we have demonstrated that copy number variations (CNVs) of individual genes or chromosomes are under strong natural selection in experimental evolution systems (Prieto et al., Nature Ecol Evol 2017, Bussotti et al, bioRxiv and PNAS in press) and in clinical parasite isolates (Bussotti et al., mBio 2018; Bussotti et al., Microbial Genomics 2020, Boité et al., Communications Biology 2021). Our studies raised the essential question on how *Leishmania* harnesses genome instability for fitness gain and can distinguish beneficial from deleterious gene dosage effects.

The current manuscript provides the response to this important open question and generated **unprecedented insight into mechanism of parasite adaptation** by combining experimental evolution with comparative genomics, transcriptomics and proteomics analyses. Our data uncover an important role of gene CNVs in driving increased mRNA and protein abundance during fitness gain for genes implicated in RNA turnover. These proteins in turn establish a **regulatory, post-transcriptional interface** that can eliminate toxic gene dosage effects by selective mRNA degradation. We further established a first link between *Leishmania* fitness gain and the differential expression of small nucleolar RNAs (snoRNAs), which caused significant changes in rRNA modification, thus revealing the **existence of fitness-adapted ribosomes.** These specialized ribosomes provide a remarkable **proteomic robustness** to adapting parasite populations and likely further compensate for deleterious gene dosage effects via **epitranscriptomic control of protein translational**.

Our findings propose a novel model for *Leishmania* fitness gain, where differential regulation of mRNA stability and the generation of fitness-adapted ribosomes may potentially filter deleterious from beneficial gene dosage effects and drive highly robust fitness phenotypes of genetically heterogenous, adapting parasite populations, thus avoiding genetic death despite constant selection.

2. Point-by-point description of the revisions

Reviewer #1 (Evidence, reproducibility and clarity (Required)):

Summary:

Leishmania are human pathogens with massive impact on human health, almost globally. The promastigote life cycle stage can be cultured in animals but also in culture. To understand virulence and infections, it is important to understand differences in gene expression between culture adapted and non-adapted parasites and, on what level these differences are regulated (Leishmania has no transcriptional control). Towards this aim, the authors have analysed changes in gene expression between culture-adapted and non-adapted parasites. They provide an extensive, comprehensive dataset that analyse and compare changes in mRNA levels, genome stability (aneuploidies), protein levels, small RNAs and rRNA pseudouridinylations. The major outcome is that fitness gain of culture-adapted parasites is regulated on several levels, by gene dosage, mRNA stability but also by translation: most interestingly, the authors provide evidence for the presence of fitness-adapated ribosomes. In general, this is a highly valuable dataset for the Leishmania community, and, as far as this reviewer (no big data expert) can judge this, they are of high quality and mostly well presented.

Response: We thank the reviewer for the time spent on evaluating our pre-print and for appreciating our findings.

Major comments:

Go-term analyses could be presented better. The authors should include the total numbers of genes in all their Go-term analysis.

Response: We thank the reviewer for this comment. We explain now in more detail the GO analyses in Material and Methods and figure legends. The total number of genes has been added to the GO tables, which now include the following values:

- **'total frequency', which represent the total number of genes in the genome associated to any of the defined GO terms compared to the total number of annotated genes,**
- **'cluster efficiency', which represents the percentage of genes in a dataset associated with a given GO term compared to the total number of genes with any annotation in genome,**
- **'enrichment score', which represent the percentage of enrichment per GO term compared to the total number of genes sharing the same GO identifier in the genome.**

It would also be good to include the total number of upregulated mRNAs with a function in ribosome assembly, without the overlap that certainly is found between Go-terms like "ribosome biogenesis", "rRNA processing" and many others.

Response: We appreciate the reviewer's comment. The Gene Ontology vocabulary is hierarchical and indeed for the case indicated by the reviewer, all the genes from ribosome assembly, RNP complex and rRNA processing are part of the ribosome biogenesis function as they belong to the same GO hierarchy. We prefer to keep this redundancy as it reflects the GO analysis. We have now added graphical versions of the GO analyses to Figure S3 to better illustrate this hierarchy.

Thus, for example, how many of the 345 upregulated genes are really involved in ribosome synthesis, the majority or a small fraction?

Response: We thank the reviewer for this comment. *Leishmania* is a very divergent eukaryote with ca 50% of genes encoding for hypothetical proteins. Thus, from the 344 upregulated genes, only 134 genes (38.95%) are annotated with a GO term. From these, 13 genes (9.7%) have a GO annotation for ribosome biogenesis. This information is presented in Figure 2 and included in Table 2-o that summarizes the GO enrichment analyses performed from the RNAseq data. In addition, we now provide in the same Table 2-o the enrichment score as described above (e.g. for the GO term 'ribosome biogenesis' the enrichment score is 15.5%). We have modified Figure 2C, right panel, which now presents the percentage of genes per category after manual categorization (see the sheet 'Category' in all tables and the Materiel and Methods section for the details on how we performed the manual categorization).

Likewise, in Figure 2D, it looks as if the LP1 stat down regulated Go-terms mostly reflect one or two upregulated mRNAs? If so, this would be better shown directly (which gene) rather than as Go-term analysis.

Response: We thank the reviewer for this suggestion. Similar to above, only 479 genes (67.5%) of the 710 genes with reduced transcript abundance in LP stat have a GO annotation. The information on the number of genes for each of the GO terms is available in Table S2-o. We have added this information to the legend. In the text, we now point only to GO terms with more than 2.5% enrichment. The GO terms 'meiotic recomb.' and 'centriole replication' - which are associated with only 3 genes - are now presented as part of the category 'cell cycle/DNA metabolism' after the manual categorization (see 'genes/category' in Table S2-n and Figure S3F).

Likewise, in Figure 4E, the data could be presented better and with more detail, again, it would be great to include the total numbers (what percentage of these DNA-only generated genes has a function related to the flagellum)?

Response: We apologize for the convoluted presentation. We now refer to Table S6-c where this information is available and have added the following information: "Gene ontology analysis of the 452 proteins that fall into regulatory cluster 1.2 revealed a strong enrichment for the term 'post-transcriptional regulation of gene expression' supported by 27 proteins (15% of all proteins that show increased abundance in LP parasite) (Figure 4E and S6D, Supplementary Table 6-b, -c and -f)… In addition, the enrichment for the GO term 'ciliary cell motility' is driven by the under-representation of this process in the LP proteome as shown by the reduced abundance of 20 proteins (18% of the proteins less abundant in LP) (Figure S6E and Supplementary Table 6 -b, -c, -g and –h)."

One major conclusion is that the changes in proteomes are surprisingly reproducible between experiments (71% of differentially expressed genes shared between four experiments). How does this compare with the reproducibility of mRNA changes and changes of the genome? I was under the impression, that at least the changes of the genome are also very reproducibly.

Response: Indeed, some adaptive genomic changes are highly reproducible between evolutionary experiments, such as trisomies of chr 5 and 26, which clearly are key driver mutations for fitness gain in culture. Aside these, there are many less reproducible 'passenger' copy number changes both on chromosome (Figure S4B) and gene levels (see Bussotti et al., 2021. doi: https://doi.org/10.1101/2021.06.15.448517). A similar tendency was observed from the second transcriptomic analysis on independent evolutionary experiments that we have now included in this publication (see Figure S3D and response to reviewer 3 below). Nevertheless, it is clear that despite this genetic and transcriptomic heterogeneity, there are mechanisms in place that can confer phenotypic robustness as seen at proteomics level, most likely at the level of translational control.

<u>**Minor comments**</u>

Is it possible to say how much of the increase in translation in LP is caused by an increase in translation efficiency and how much is caused by an increase in the number of ribosomes? Perhaps by measuring translation via Puromycin labelling and correlate these changes with the change in ribosome numbers? Can ribosome numbers be calculated from the increase in ribosomal proteins (proteomic data) or perhaps from the A260 measurement of total RNA preps from equal cell numbers or from a northern blot probed with rRNA?

Response: We thank the reviewer for these excellent suggestions. Indeed, we are initiating a new investigation on translational regulation performing ribosomal profiling with RiboLace, which will be subject of a future manuscript.

Figure 5C: why is LD14Cs1H3 a loading control? How can the authors be sure that this RNA does not change?

Response: This has been chosen as loading control as neither the gene copy number nor the transcript abundance (by RNA-seq) was affected in our experiments.

Introduction: "Trans-splicing is also found for example in Bodonidae, the authors should avoid to call it "parasite-specific".

Response: We thank the reviewer and have removed this statement.

"Surprisingly, this defect in metacyclogenesis was compensated in cultures that were passaged using enriched EP.1 meta rather than EP.1 stat..." It's the late procyclics, that have the defect in metacyclogenesis, not the early? Please clarify.

Response: We appreciate the reviewer's comment and have clarified this section by rewording as follows: "Surprisingly, unlike observed when passaging EP.1 stat parasites, metacyclogenesis was maintained in cultures that were passaged using metacyclic-enriched parasites (EP.1 meta)…."

For the non-Leishmania experts: Life cycle in the introduction only mentions promastigotes, but does not explain metacyclogenesis and how promastigotes and metacyclics differ etc.

Response: We thank the reviewer for this suggestion and have added the following information to the introduction: "After migration towards the stomodeal valve, they eventually differentiate into the infectious metacyclic form which is transmitted to the mammalian host during the blood meal. Once phagocytosed by host macrophages, metacyclic promastigotes differentiate in the non-motile, intracellular amastigote form that proliferates inside fully acidified, macrophage phagolysosomes of mammalian hosts"

Can the authors also provide more details on the the different in vitro differentiation methods (enrichment of metacyclics, stationary phase)?

Response: We apologize and have added a more detailed description to the Methods section. "Promastigotes in exponential growth phase were seeded at $2x10^6$ (EP) or $1x10^5$ (LP) parasites per ml in M199 complete medium. The different seeding densities allowed to compensate for the difference of EP and LP parasites in growth and to guarantee that they both reach stationary growth phase at the same time, which is essential for the comparative analyses of stationary-phase and metacyclic parasites."
And:
"EP and LP promastigote cultures were maintained at stationary phase culture for 3 days when cell density, acidification and nutrition depletion triggers the differentiation from procyclic to metacyclic promastigotes." Parasites were then either used as stationary phase promastigotes or for metacyclic enrichment by Ficoll gradient centrifugation.

Likewise, for the non-Leishmania experts: This reviewer was surprised to see, how reproducible this massive appearance in aneuploidy occurs between 3 independent culture adaptations, and how fast it occurred. Is this really a random process (survival of the fittest) or is this a regulated process? There is some information in the discussion, but it would help to provide this information a little earlier, in introduction or the respective result section.

Response: We thank the reviewer for this comment. We added the following statement to the results: 'We previously observed that tissue amastigotes (in infected hamster spleens) represent a mosaic karyotype, with monosomies and trisomies observed for all analyzed chromosomes, including chr 5 (Prieto et al, Nature Ecol Evol 2017). Based on this result, the reproducible emergence of chr 5 and chr 26 trisomies in different culture adaptation experiments represents a passive, convergent process that relies on the positive selection of pre-existing sub-populations, rather than an active, regulatory process driving karyotypic adaptation.'

Figure 5B: replace ',' with '.'

Response: Replaced.

Many other studies are already available, that look at RNA, protein and translation (ribosomal profiling) in Leishmania. The authors should be careful with phrases like "Our study provides first evidence that these parasites combine regulatory processes at genomic, post-transcriptomic and translational levels to establish highly robust fitness phenotypes while maintaining genetic heterogeneity thereby avoiding genetic death."

Response: We thank the reviewer for this comment. Indeed, while these regulatory processes have been studied in the context of stage differentiation or the response to stress, they were not yet linked to fitness gain in culture. Nevertheless, we toned down our statement throughout the text by deleting 'first evidence' and similar expressions.

Data on flagella appear rather isolated in Figure S2; best to omit or explore further.
Response: We thank the reviewer for this suggestion and have opted to explore this further. We added the following information to Results: "As expected from the increased motility described for metacyclic parasites, we indeed observed increased abundance in EP.1 meta compared to EP.1 log and EP.1 stat for respectively 48 and 51 genes linked to motility and flagellar biogenesis" and provided a table for the details (Supplementary Table 2-p).

<u>Reviewer #1 (Significance (Required)):</u>

Not everything is completely new, it is known (and also expected) that gene regulation is done on several levels. And, not all effects described are "major": often, the changes are rather small. Still, to the best of our knowledge, such a comprehensive dataset (in one go, with the minor

drawback that the proteomics data came from a different experiment) is not yet available, and will be useful for the community. Moreover, the data on the small RNAs and on the ribosomal modifications are highly interesting and will pave the way towards analysing the presence and function of fitness adapted ribosomes in Leishmania.

Response: We thank the reviewer for the constructive criticism. The novelty lies in the fac that we link fitness gain in culture (rather than differentiation or stress response) to post-transcriptional regulation, ncRNA expression and proteomic robustness. According to our data, these processes likely act in concertation to compensate toxic gene dosage effects. We agree that this may have been expected and will tone down our conclusions accordingly.

Reviewer #2 (Evidence, reproducibility and clarity (Required)):

Summary

The study presented in this paper investigates whether genome instability is responsible for the generation of phenotpypes responsible for survival during the changing environments that the parasite experiences throughout its life cycle. The study was performed in vitro. Parasites were extracted from infected animals, and allowed to go through five passages (Early passage, EP) or twenty passages (Late passage, LP). The authors report on a "fitness trade off" whereby selection for accelerated growth comes along impaired infectivity. The authors perform comparative genomics, transcriptomic and proteomic studies on EP and LP cells using log, metacyllic and stationary phase cells, and suggest that there is a complex regulatory network that links changes in gene dosage with post transcriptional changes generating parasite fitness. The authors report that gene dosage reduction in flagellar transcripts is coordinated with an increase in non-coding RNAs involved in ribosome biogenesis and protein translation. The authors show a correlation between expression of snoRNAs with changes in rRNA modifications that affect ribosome assembly.

This is an interesting study that provides a broad range of experimental data, leading to their conclusion that Leishmania fitness gain may be controlled by post-transcriptional processes combined with epi-transcriptomic regulation. It is still not clear how these affect the changes in gene dosage though. The authors invested an impressive amount of work, which is important to the community, and may imply on other organisms as well.

Some of the experimental data require a better description, as indicated below.

Some of the data are over-interpreted.
Response: We thank the reviewer for the positive feedback and the constructive comments. We apologize for the over-interpretation and have toned down the manuscript as indicted below.

Major comments

Figure 2C&D - the figure shows the differential expression profiling of LP.1 and EP.1 log parasites grouped according to their stage. The legend does not fit the text and the figure.

Response: We thank the reviewer for this comment and apologize for the confusion. We modified the text and the figure legend accordingly.

Also - it is not clear why the authors include transcripts that their FC change is less significant ($p>0.01$) and do not state the range of p values that they relate to in the figure.

Response: We thank the reviewer for this comment. In the Figures 2 C and D (volcano plot in the left panel) the transcripts with lower fold changes and adj. p-values > 0.01 are shown as black and grey dots but they were not considered for the functional analyses. Therefore, Figures 2 C and D (middle and right panels) refer to transcripts with FC > 1.5 and adj. p-value < 0.01. We clarified this in the figure legend and the text.

The legend of figure 2C does not indicate what is "up" and "down" that is shown in Figure 2C

Response: We have added this information in the legend and the figures 2 C and D.

Eventually, it appears that a decrease in LP1 log parallels an increase in EP1 log, but this is not indicated clearly neither in the text or in the legend. These should be written more clearly. Same clarification is required for the legend/text of Figure 2D.

Response: We thank the reviewer for this comment. Indeed, our comparative analysis of expression changes between EP and LP expression changes implies that a decrease in one is mirrored by an increase in the other (and vice versa). We chose to express and discuss all expression changes with respect to LP parasites (LP up or LP down) as the publication is geared towards the fitness gain in culture and the fitness cost in vivo that characterize the LP parasites. We clarified the legend to state clearly that the transcripts more abundant in EP correspond to transcripts less abundant in LP.

Table 2e - the authors ran a manual inspection of the transcript categories. They should therefore categorize the transcripts into groups, otherwise it is difficult to follow their analysis.

We thank the reviewer for this comment. We added the following tables: Supplementary Table 2-i and -m corresponding to ‘Category’ for transcripts more abundant in LP log (LP log up) and LP stat (LP stat up) and Supplementary Table 2-j and -n corresponding to ‘Category’ for transcripts less abundant in LP log (LP log down) and LP stat (LP stat down). Likewise, we added sheets to all the other Supplementary tables to group genes or proteins by category.

It is not surprising that LP1 log cells under rich nutritional conditions retool their gene expression pathways towards energy production rather than for motility functions. As metacyclic cells are equipped with a long flagellum and are known to be highly motile, as in the fly they must leave the hind gut and move towards the from parts of the mouth. This comparison should be mentioned.

Response: We thank the reviewer for this suggestion and have modified the text as follow: "These pathways suggest a potential retooling of the LP.1 log energy metabolism in the nutrition-rich culture environment, and selection against motility, which is not essential in culture and may liberate the energy required for faster growth."

Figure 2C - In the text (page 15 of the text) the authors relate to a group that is not represented in the GO analysis.

Response: We thank the reviewer for this comment and corrected the text.

They claim that there are 437 transcripts showing reduced abundance in LP.1 log phase, but they don't see them in the GO analysis. Is the reduction statistic?

Response: We thank the reviewer for this comment. We confirm that the 437 transcripts used for the GO analysis showed indeed statistically significant reduced abundance in LP.1 log parasites (fold changes > 1.5 and adj. p-values < 0.01). As judged by the GO analysis, this dataset simply doesn't show any enrichment for a given GO term. We clarified in the text and legend that only the transcripts with statistically significant reduction were considered.

How can one judge the results? perhaps it would be helpful if they included a manual categorization of the transcripts, indicating the degree of change and p value. These are basic factors that are required for making meaningful conclusions.

Response: We thank the reviewer for this comment. We manually assigned genes or proteins with statistically significant changes (FC > 1.5 and p-values <0.01) to categories. Categories are presented in Supplementary Tables 2, 2bis, 4, and 5. In Supplementary Table 6 the manual categorization was performed on genes/proteins whose changes are associated with p-values < 0.01.

Regarding the statement that there is a decrease in 5S protein - do the authors relate to a specific protein of the small ribosomal subunit or to 5S RNA?

Response: We thank the reviewer for this comment. We referred to the 5S RNA and made it clear in the text.

The authors relate to the "quality or type of ribosomes" - they should be more specific. How do they determine the quality of ribosomes? Do they relate to a stoichiometric comparison between the different ribosomal proteins? This is not indicated.

Response: We thank the reviewer and extended the discussion on the possibility of specialized ribosomes by adding the following text: "The existence of such structurally distinct, specialized ribosomes has been observed in *Plasmodium* spp., where stage-specific expression of certain rRNA isoforms allows for the establishment of A-type (asexual stage specific) and S-type (sporozoite specific) ribosomes (McCutchan et al., 1988; Li et al., 1997). Likewise, stage-specific modification of rRNA has been linked to the transition of *T. brucei* from the procyclic insect to the mammalian bloodstream forms (Chikne et al 2016). Finally, changes in expression and modification of different rRNA genes, ribosomal proteins, and translation factors indeed can control preferential translation of different subsets of mRNAs in other organisms'.

Figure 2C - Can the authors explain why the "up" genes in LP1 log include genes that increase the ribosome population, and why the "down" genes in this stage include genes involved in translation.

Response: We apologize for the misleading information presented in figure 2C. All the panels of this figure refer to transcripts more abundant in LP. We clarified the figure and the corresponding legend.

The authors should be more specific on the type of translation factors that are decreased. Are these involved in translation initiation? Elongation? tRNA synthetases? etc.

Response: Components of the translation machinery (ribosome/translation category) are decreased in LP log down but not the translation factors (Supplementary Table 2-j). Inversely, transcripts in this category are more abundant in LP log up including for the gene Ld1S_330607800 encoding for the translational initiation factor IF-2 (Supplementary Table 2-i).

Figures 2C and D - The text in the Results section and the legends should be written more clearly.

Response: We thank the reviewer and made many changes (also in response to the reviewers' comments) that will render the Results section and legends clearer.

Figure 2D - It is not clear how the analysis identified genes in the arg biosynthesis process, while Leishmania do not synthesize arginine. Please elaborate.

Response: We thank the reviewer for this comment. Three genes were associated with the GO term arginine biosynthesis process, LD1S_160116600 (carbamoyl-phosphate

synthase), LD1S_230252800 and LD1S_230252400_PSEUDOGENE (argininosuccinate synthase, putative). The carbamoyl-phosphate synthase acts very early in the arginine biosynthetic process to generate carbamoyl phosphate that is also part of pyrimidine biosynthesis process which applies for Leishmania. The argininosuccinate synthase participates in the urea cycle and was associated to the parasite pathogenesis (Lakhal-Naouar et al 2012) which could explain its reduced abundance in the less infectious LP parasites. However, we removed the link to arginine biosynthesis process from the text as only 3 genes were concerned. The argininosuccinate synthase was manually assigned to the virulence category.

Figure S4 - The authors should discuss the ploidy of chr 31 and its related gene clusters.

Response: We thank the reviewer for this comment. We included chromosome 31 in Figure S4C and have added the following: "In contrast, the tetrasomy of chr 31 is stable and has been observed in all *Leishmania* species (Rogers et al, 2011) and in *ex vivo L. donovani* amastigotes (Prieto-Barja et al, 2017; Dumetz et al, 2017). Given the stability of this tetrasomy, regulation of expression via karyotype-dependent gene-dosage effects seems not to apply to chr 31. Thus, the expression changes between EP.1 and LP.1 observed for 144 genes are likely regulated at post-transcriptional levels (Supplementary Table 4-b)".

Also - some choromosomes appear to have a mixed ploidy (1, 9, 12, 15, 23, 32). Does this indicate that the populations are mixed? Would it be possible that these populations are in the process of increasing the ploidy of these chromosomes? The authors should discuss this possibility.

Response: We thank the reviewer for this comment. We have added the following: "An increased somy score was observed for these chromosomes already in EP.1, indicating a mosaic of disomic and trisomic sub-populations, the latter one showing full penetrance in LP.1. Indeed, we previously observed that tissue amastigotes (in infected hamster spleens) represent a mosaic karyotype, with monosomies and trisomies observed for all analyzed chromosomes (Prieto et al, 2017)".

With respect to gene dosage changes - the authors examined only the formation of trisomic chromosomes. Did they examine the possibility of gene amplification within a given chromosome, or the appearance of episomes that are responsible for amplification of genes expected to increase their expression.

Response: We thank the reviewer for this comment. We have added an additional figure (S4A right panel) showing the genomic ratio LP/EP over the entire genome and added the following information to the text: "In contrast to the dynamic changes in karyotype, no significant fluctuations in LP.1/EP.1 read depth ratio was observed across the genome mapping the reads to 300bp genomic bins, thus ruling out episomal or intra-

chromosomal amplifications as drivers of culture adaptation, at least during the first 20 passages (i.e. in LP parasites)."

Tables 1-6 : Functional clustering of the different genes would help follow the conclusions indicated in the text, i.e. where can one follow the different clusters indicated in Table 6?

Response: We thank the reviewer for this comment. We manually assigned genes or proteins with statistically significant changes (FC > 1.5 and p-values <0.01) to categories. Categories are presented in Supplementary Tables 2, 2bis, 4, and 5. In Supplementary Table 6 the manual categorization was performed on genes/proteins whose changes are associated with p-values < 0.01.

Table 6 it appears that there are different clusters with respect to the mechanism that regulates protein expression: RNA turnover, Translation regulation, Gene dosage, etc. Can the authors indicate whether there is any biological logic between the different mechanisms?

Response: We thank the reviewer for this comment. Aside from the presence of 6 chaperones from chromosomes 5 and 26 in cluster 1 that was highlighted in the text we did not identified any other biological signs. We have added the following in the text: "The increase in protein abundance is the combined result of increased gene dosage and increased mRNA abundance for the vast majority of proteins (83%) encoded on trisomic chr 5 and 26."

Figure 4D&E - The increase in chaperone expression, as part of Cluster 1 on chr 5 and 26 cannot indicate that these genes are involved in inducing aneuploidies. The use of designated mutant could support this hypothesis, but as of now - this is an over-interpretation of the data that should be avoided.

Response: We thank the reviewer for this comment and toned down the text accordingly. Indeed, it would be interesting in a future study to genetically test the role of chaperones in culture adaptation using loss- and gain-of-function studies.

The authors target the reader to Table 6, but it is impossible to "dig" the relevant genes without their categorization. As indicated before - all excel tables should include categorized into groups, to allow the reader to follow the author's statements.

Response: We thank the reviewer for this comment. We manually assigned genes or proteins with statistically significant changes (FC > 1.5 and p-values <0.01) to categories. Categories are presented in Supplementary Tables 2, 2bis, 4, and 5. In Supplementary Table 6 the manual categorization was performed on genes/proteins whose changes are associated with p-values < 0.01.

In cases in which the trisomy is incomplete, did the authors consider the possibility that the increase of gene dosage was done on episomes rather than on whole chromosomes?

Response: We thank the reviewer for this comment. We have included an additional figure (S4A right panel) showing the genomic ratio LP/EP over the entire genome and added the following information to the text: 'In contrast to the dynamic changes in karyotype, no significant fluctuations in LP.1/EP.1 read depth ratio was observed across the genome mapping the reads to 300bp genomic bins, thus ruling out episomal or intra-chromosomal amplifications as drivers of culture adaptation, at least during the first 20 passages (i.e. in LP parasites).'

Discussion: The conclusion that Leishmania harbor "specialized ribosomes" is indeed tempting and novel but lacks sufficient experimental data. There is not enough experimental data to support this conclusion. To further elaborate on such a possibility the authors should have shown that different ribosomes are loaded with distinct transcripts. Without such data the authors should mellow down their conclusions on the generation of "specialized ribosomes" as part of the fitness process that they describe. This is an over-interpretation that should be avoided.
Discussion: The authors try to explain why they see that in LP1 there is an increase in ribosome metabolism proteins, but not in translation. It is still not fully clear how they determine differences in the quality of ribosome components, and how this is related to translation. Do they see a difference in polysomes?

Response: We thank the reviewer for this comment and have toned down these statements. We also extended our discussion on this aspect, which lends more support to the existence of specialized ribosomes (see response above). Finally, in a separate study performed in *L. major*, we have shown that depletion or over-expression of a single snoRNA and the respectively increased and decreased modification of its rRNA target indeed changes the ribosome as it affected the translation of only a minor and highly reproducible subset of proteins (and determined by quantitative LC analyses). These data are prepared for publication. We have added below a confidential figure showing these data.

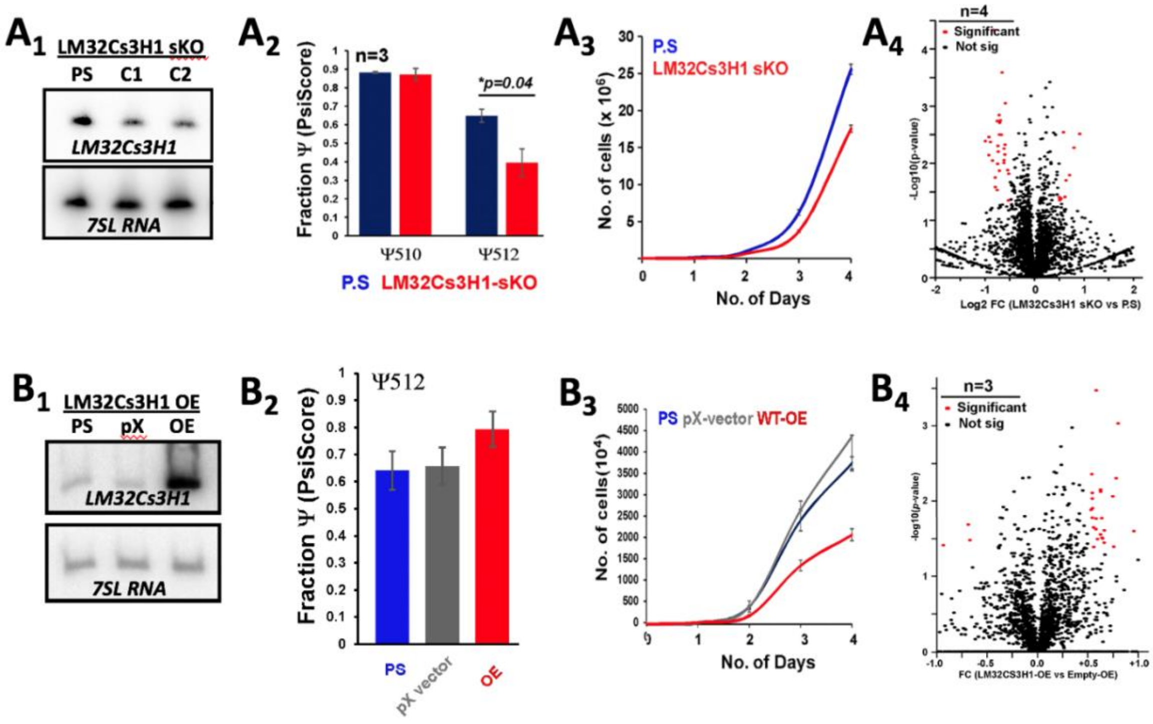

Changes in rRNA modification affects ribosomal translation specificity in *L. major*. (A) Single knock out (sKO) for the snoRNA LM32Cs3H1. (A1) Northern analysis demonstrating the reduction in the level of snoRNA in the sKO clones C1 and C2. (A2) Reduction in the Ψ level in sKO using Hydra Ψ-seq. (A3) Growth curve of a sKO clone. (A4) Volcano plot showing deregulated proteins in red in the two sKO clones compared to the parental stain. (B) Gain-of-function analysis by over-expression (OE) of the snoRNA LM32Cs3H1. (B1) Northern analysis showing the overexpression of LM32Cs3H1. (B2) Increased Ψ level in OE strain using Hydra Ψ-seq. (B3) Effect of snoRNA overexpression on growth. (B4) Proteome analysis showing 28 de-regulated proteins upon snoRNA overexpression.

Minor comments

Figure 1E and S2A-C - Please describe how the ratio between the flagellum and cell length values was determined.

Response: We have added the following details in the methods section and the figure legend: "Length and width of the parasite cell body, and flagellum length were measured for at least 200 promastigotes using the Image J Fidji software package (https://imagej.net/). The ratios flagellum over body length and body length over body width were determined for the 200 parasites and the Kruskal-Wallis test was used for statistical analysis."

Figure 1E - the authors should provide a clear description of the different labels in the legend.

Response: We have added information in the figure and the legend

Figure S2A - please show a broad field of the cells that served for the measurements of the flagellum and cell dimensions. It is not sufficient to show a single cell.

Response: We have added broad field images in the figure.

Figure 2C - right panel - why is it grey?

Response: We have modified the color to dark cyan which corresponds to LP

Reviewer #2 (Significance (Required)):

Leishmania are known to change their infectivity following long-term passages in vitro. The biological basis for this reduction is not fully understood.
The genome of Leishmania is known to show a high plasticity, especially under selective conditions. Following gene clusters that are encoded on chromosomes that increased their dosage from diploid to trisomy, is of importance to the field. Parasite virulence is a factor that is not fully understood in terms of complex regulatory networks that combine, gene dosage, regulation of RNA level and translation. This paper sheds light on this complex and unstable feature.

The modification of small non-coding RNAs, such as snoRNAs and their effect on ribosomal RNA pseudo-uridylation is a novel observation that deserves additional experimental attention in the future.
The paper is of great importance to researchers working on Leishmania and trypanosomes, but could have implications on research on other fields as well.

Response: We very much appreciate the reviewer's interest in our data and the highly constructive comments.

Reviewer #3 (Evidence, reproducibility and clarity (Required)):

Summary

The study investigates changes and mechanisms involved in Leishmania "long-term" (20 passages) adaptation to promastigote culture and its consequences on subsequent in vitro macrophage infection. It observes a fitness tradeoff of the evolved culture adaptation between both environments with faster growth in culture leading associated with very reduced capability to infect in vitro macrophage culture. For this example of Leishmania adaptation to a new (lab) environment they address the question how Leishmania adaptation, which has often been associated with copy number changes in a subset of chromosomes can benefit adaptation

without the typical negative effects that other species experience upon aneuploidy changes. To address these questions, they use a good range of techniques including DNA-seq, RNA-seq, proteomics and analysis of the small RNome by RNA-seq. With this approach they observe chromosome copy number changes, differential gene expression between short and long-term lab evolved Leishmania. They also identify possible mechanisms that can contribute to the indirectly observed posttranscriptional control of transcript and protein abundance, a mechanisms that sheds light on how Leishmania can benefit from aneuploidy changes during adaptation, while not suffering from the chromosome wide effects of gene dosage change.

Major comments

Transparency of the experimental (evolution) design:

The experimental evolution design lacks clarity and how it was generally designed only because apparent step by step while reading through the methods and results. I think it would benefit greatly if there would be a paragraph and even a (supplemental)figure on the design in the beginning of the methods stating the number of replicate populations used for the experimental evolution experiment and from which exact stating material they were set up and if replicates were run in parallel or sequentially. Then sample material and sample type taken at different time points and of different evolutionary replicates should be clearly indicated. It would even be helpful for the reader to state the rationale behind the different type of samples taken to be aware of all those aspects upfront.

Response: We thank the reviewer for this comment. We have added two figures (S8 and S9) that summarize the experimental workflows and information in the method section

That being said, from reading the manuscript I understood that needed replication for the experimental evolution aspect was not done. This seemed to be the case for the phenotypic measurements (only sampled from evolutionary replicate 1, EP.1. and LP.1), for DNA-seq to estimate chromosome copy number (EP.1 and LP.1, LP.6, LP.7), for RNA-seq (EP.1 and LP.1, not sure how the 3 replicates shown were taken but I guess they only sample variation from within evolutionary replicate 1). Only protein abundance was measured based on three evolutionary replicates (P.2-5 and LP.2-5).
To obtain robust conclusions evolutionary replication with n=1 is simply not sufficient, so generally replication typically of n=3 should be present. This is particularly the current study for the RNA-seq data, were some expression changes could have occurred by neural mutations in the culture environment that are nevertheless picked of as a signal in the analysis. To eliminate expression changes that do not contribute to the culture adaptation variation from several evolutionary replicates have to be taken into account. If replicates cannot be added for the different types of measurements cannot longer be added the limitations of the shown results have to be very clearly stated. Particularly RNA-seq results cannot be interpreted to have a general functional relevance for the indicated fitness changes.

Response: We thank the reviewer for this comment. Indeed, we have performed and analyzed several in vitro evolution experiments, only one of which was shown in the original manuscript. We have now included independent datasets for phenotypic and RNAseq analyses obtained by additional evolutionary experiments (Figures S1C and S3D respectively), which provide strong evidence for the reproducibility of our observations. The detailed description of these samples is presented in Figure S8 and S9. For the phenotypic analysis, we have included the data of one new evolutionary experiment (EP.luc nd LP.luc) and cited the independent evolutionary experiment that we previously published (Prieto-Barja et al 2017). For the transcriptomic analysis, we have added the RNAseq data set of an additional, independent evolutionary experiment (EP.8 and LP.8) and two independently analyzed EP and LP samples (EP.9 and LP.10). The details are presented in figure S8.

Some specific remarks to this general topic:

p5 "Promastigotes, derived from splenic amastigotes, were serially passaged once stationary phase was reached"
How many independent "replicates" were made this way and where they all made from the same extraction of splenic amastigotes? Please state exact differences between independent cultures in more detail.

Response: We thank the reviewer for this comment. We have added two figures (S8 and S9) that summarize the experimental workflows and present all the biological samples that were used, including the infected hamsters used to purify the amastigotes and the different EP/LP parasites we derived from these amastigotes. For the transcriptomic analysis of EP.1 and LP.1 as well as for all the phenotypic analyses we have used three cultures generated from three different frozen aliquots (see Figure S9). As previously mentioned, we have now included independent datasets for phenotypic and RNAseq analyses. The details are presented in Figure S8.

p5 "seeded at 2x106 (EP) or 1x105 (LP) parasites per ml"
Why at different densities? I understood the difference in both treatments is the number of passages but not the starting densities or the interval lengths between passages.
It would also be good to state at this part in the methods why these different treatments were taken, i.e. which hypothesis / aspect was going to be tested with it.

Response: We thank the reviewer and have added the following explanation: "Promastigotes in exponential growth phase were seeded at $2x10^6$ (EP) or $1x10^5$ (LP) parasites per ml in M199 complete medium. The different seeding densities allowed to compensate for the difference of EP and LP parasites in growth and to guarantee that they both reach stationary growth phase at the same time, which is essential for the comparative analyses of stationary-phase and metacyclic parasites."

p6 "EP.1 and LP.1, LP.6 and LP.7 promastigotes"
Why these experiment numbers and how many were there in total. What do you mean with the term "experiment" compared to "replicates"?

Response: We have included the experimental workflows that should clarify this point for the readers (Figure S8 and S9). Strains issued from independent experimental evolution assays are identified by number (i.e. EP.1 and LP.1 are the strains resulting from experiment 1, see Figure S8 for details). For comparative analyses DNA, RNA or proteins were extracted from the different EP and LP logarithmic, stationary or metacyclic-enriched parasites as presented in Figure S8 and S9. Phenotypic characterization were performed on three different cultures prepared from EP.1, LP.1, EP.luc and LP.luc using frozen aliquots as starting material (see Figure S9). This information has been included in Material and Methods.

p9 "A generalized linear model including the replicate effect as blocking factor was set in order to test for the differential expression between EP.1 log, EP.1 stat, EP.1 meta, LP.1 log and LP.1 stat."
The design for the expression analysis is unclear to me. The details of it and how replication was exactly taken should also be included in the supplemental experimental design figure asked for above.

Response: We have added Figure S9 for the experimental workflow.

Minor comments

p1 "Post-transcriptional regulation of Leishmania fitness gain"
I do not find this title appropriate. It is not clear what it should even mean?
In the study they identify / observe that posttranscriptional mechanisms affect observed transcript and protein abundance. Those can also be important to modulate effects on expression of somy changes that are observed during promastigote culture adaptation. "fitness gain" is not appropriate and very unclear as used. Fitness gain where? I suggest to replace with sth like "adaptation to promastigote culture" or similar.

Response: We thank the reviewer for this comments and have changed the title to ‘Experimental evolution reveals post-transcriptional regulation as a novel driver of *Leishmania* fitness gain’

p2 "selection for accelerated growth (fitness gain)"
Change to "selection for accelerated growth in promastigote culture (fitness gain)"

Response: Done in the text.

Although selection for maximal selection for accelerated growth you would always passage while still in the exponential growth phase.

Response: Our intention was not to select for maximum growth but just allow parasites to adapt to culture using a classical protocol, which implies to split cells once they reach stationary phase.

Also keep in mind that using "fitness gain" and "fitness cost" is always environment dependent the observed gain is only in promastigote culture and the observed cost is only when infecting in vitro macrophages. Throughout the manuscript these are used in a bit unspecific way making the environment aspect not very clear, which can especially be misleading for readers with a non-evolutionary background.

Response: We appreciate this comment and have specified now different environments as suggested by the reviewer.

p2 "... as a potential novel source for biomarker discovery."
Not clear how this is meant.

Response: We changed to '…identifies the *Leishmania* transcriptome and non-coding small RNome as potential novel sources for the discovery of biomarkers that may be associated with parasite phenotypic adaptation in clinical settings.'

p3 "Most Leishmania species show a digenic life cycle"
I don't think digenic is correct here, implying it is caused by two genes?!

Response: We thank the reviewer and corrected to the correct term "digenetic".

p4 "evolving to higher fitness in culture"
Suggest to change to "adapting to culture conditions"

Response: We thank the reviewer and corrected the text.

p9 "For this purpose the nucleotide coverage was normalized by the median genomic coverage."
How, divided by it and assumed it corresponds to diploidy?

Response: The following information was added to Material and Methods: "Samtools was run with options "-q 50 -F 1028" to discard reads with a low map quality score or potential duplicates, while BEDTools genomecov was run with options "-d -split" to compute the coverage of each nucleotide. The coverage of each nucleotide was divided by the median genomic coverage. This normalization is done to account for library size differences." This precision was added to the method section.

"window coverage was further normalized by chromosome 36 median coverage"
Why chromosome 36 median? Would be helpful for some audience not only say what was done but add the rationale behind it.

Response: We thank the reviewer for this comment. We have added the following in the method section: "As previously published (Prieto-Barja et al, 2017), the stably disomic chromosome 36 was used to normalize chromosome read depth and to estimate chromosome polysomy levels in each sample".

p9 "with default parameters, was used for alignment on the reference genome (GCA_002243465.1)"
It seems there were two different reference genomes used for genomic (DNA) and RNA-seq analysis with the one for DNA being from the same strain as used in the experiments. What was the reason for it?

Response: We apologize for the confusion. The two numbers given in the text correspond to the same reference genome (GCA_002243465.1) but the link (https://www.ncbi.nlm.nih.gov/bioproject/PRJNA396645) was referring to the bioproject. This was changed to "https://www.ncbi.nlm.nih.gov/bioproject/PRJNA396645, GCA_002243465.1".

p11 The methods descriptions for the lists of L. donovani GO terms built in house is not easy to understand and based on what is written not really repeatable. It would be helpful to provide the code for the used pipeline.

Response: We now provide the code used for this pipeline in supplementary data. However, this code was generated in the context of our cluster in Pasteur, it may not be directly functional under another environment.

p14 Fig1 It is not clear on which level the comparisons between EP.1 and LP.1 were replicated. Which are the different measurements to assess within treatment (e.g. within EP.1) variation? Were there individual cultures replicating e.g. EP.1? The units of replication are crucial to know which level of biological variation (e.g. between vs. within evolving cultures) could be assessed. This comment also relates to the major comments to "Transparency of the experimental design"

Response: We thank the reviewer for this comment. As said above, we have added the experimental workflows and information in the materiel and method section for clarification. For all analyses performed EP.1/LP.1 and EP.luc/LP.luc should be considered as technical replicates as each sample corresponds to cultures prepared from different frozen aliquots (Figure S9).

Any reason why subfigures B and C have axis labels on the right hand side?

Response: We thank the reviewer and corrected the figures.

p14 "fitness tradeoff occurring during L. donovani culture adaptation, with accelerated in vitro growth causing virulence attenuation"

Inadequate wording and details of the conclusion: the observed fitness tradeoff is between environments. E.g. change to "fitness tradeoff between growth in vitro culture and infectivity of macrophages in vitro evolved during culture adaptation."

Response: We corrected throughout the text.

p14 "using enriched EP.1 meta rather than EP.1 stat"

Unclear what is exactly meant.

Response: We clarified in the text as follows: "Surprisingly, unlike observed when passaging EP.1 stat parasites, metacyclogenesis was maintained in cultures that were passaged using metacyclic-enriched parasites (EP.1 meta) (Figure 1F)".

p14 "L. donovani culture adaptation causes a fitness tradeoff between proliferation and infectivity"

Would change to "Long-term L. donovani culture adaptation results in a fitness tradeoff between in vitro proliferation and infectivity". I guess if a fitness tradeoff would already been apparent when not only comparing EP to LP but also the starting population(s) used for experimental evolution with EP.

Response: The text was changed accordingly.

p15 "obtained from three replicates of EP.1 and LP.1 log, stat, and EP.1 meta parasites"

Unclear as before what is meant. What is the level of replication, how many samples is this in total.

Response: As indicated above, we have added the experimental workflows (Figure S8 and S9) and information in the materiel and method section for clarification

p17 "Direct comparison of the normalized transcript output in EP.1 versus LP.1 revealed a global, gene dosage-independent increase in transcript abundance in LP.1 log, as would be expected for the increased metabolism in these fast-growing cells"

Not sure what is meant here. For the typical library size normalization it is not possible to observe a general expression increase across all genes as these differences typically would be erased by library size normalization when using RNA-seq.

Response: We thank the reviewer for this very important comment and fully agree. We have changed the text as follows: "Direct comparison of the normalized transcript output in EP.1 versus LP.1 revealed a gene dosage-independent increase in transcript

abundance for a large number of genes in LP.1 log (Figure 3C, left panel).”

Reviewer #3 (Significance (Required)):

This is a timely and interesting study addressing molecular mechanisms to be able to tolerate and benefit from aneuploidy changes during adaptation. From an evolutionary and population genomics perspective, my major critique is about the experimental evolution design used, which is insufficient if I understood the methods correctly.

Response: We thank the reviewer for the generous assessment and the very useful critical points raised.
