# Supplementary material for: Experimental evolution links post-transcriptional regulation to Leishmania fitness gain
Source: PLoS Pathog. 2022 Mar 16;18(3):e1010375. doi: 10.1371/journal.ppat.1010375 (PMC8959184; doi:10.1371/journal.ppat.1010375)
Supplement: Attachment — Submitted filename: Response letter PPATHOGENS-D-21-02620.docx [file ppat.1010375.s021.docx]

**Response letter**

We want to thank the Editor and the Reviewer for the positive evaluation of our manuscript. Please find below the detailed answers to the outstanding queries.

**Comments:**

- We found that some of the major conclusions/interpretations that dominate the Title and the abstract remain overly speculative (fine for the Discussion section). The ‘fitness-adapted ribosome’ model is especially speculative at this stage.

Response: We thank the reviewer for this comment and have toned down these statements. The title changed to ‘Experimental evolution links post-transcriptional regulation to *Leishmania* fitness gain’ and ‘fitness-adapted ribosomes’ in the abstract was changed into ‘modified ribosomes‘ to better reflect the data we show.

- It seems that all of the (transcriptome, small RNA-ome, proteome) changes observed may be either directly or indirectly due to gene-dosage changes. The authors refer to “gene-dosage independent” changes in their abstract, and the title appears to reflect this interpretation. Are the authors interpreting potentially ‘indirect’ effects as ‘independent’ effects here?

Response: We thank the reviewer for this comment and agree that this statement in the abstract was unclear. We changed the abstract as follows: ‘Comparative genomics, transcriptomics and proteomics analyses revealed a complex regulatory network associated with parasite fitness gain, with genome instability causing highly reproducible, gene dosage-independent and -dependent changes. Reduction of flagellar transcripts and increase in coding and non-coding RNAs implicated in ribosomal biogenesis and protein translation were not correlated to dosage changes of the corresponding genes, revealing a gene dosage-independent, post-transcriptional mechanism of regulation. In contrast, abundance of gene products implicated in post-transcriptional regulation itself correlated to corresponding gene dosage changes. Thus, RNA abundance during parasite adaptation is controlled by direct and indirect gene dosage changes.’

- As requested in the Review commons review, inclusion of an independent replicate of the experimental evolution data is important. For the transcriptomic analysis of the second RNAseq data set, were there also technical replicates for these samples, or were EP.8 / EP.9 and LP.9/LP.10 used as biologic replicates? In either case, a PCA plot would be helpful.

Response: We thank the reviewer for this comment. EP.8/EP.9 and LP.9/LP.10 are biological (and not technical) replicates as each of the two EP and two LP strains are derived from a different amastigote isolate. This is detailed in the flow chart shown in Figure S8. We added the PCA plot (Figure S3D) and clarified this in the results section.

- There appears to be no independent experimental replicate samples for the ncRNA analysis. Is this correct?

Response: The shown results are representative of two (Northern blot analysis, two repeats were performed per culture) or three independent cultures (rRNA pseudouridinylation level) derived from the same frozen stock (see Figure S9 for details). This is now specified in the legend.
