# Supplementary material for: Experimental evolution links post-transcriptional regulation to Leishmania fitness gain
Source: PLoS Pathog. 2022 Mar 16;18(3):e1010375. doi: 10.1371/journal.ppat.1010375 (PMC8959184; doi:10.1371/journal.ppat.1010375)
Supplement: Peer review history

# Author comment: Author response to previous submission

**DOI:** 10.1371/journal.ppat.1010375.r001

30 Dec 2021

Attachment Submitted filename: full-revision RC-2021-00937.pdf Click here for additional data file.

# Editor report: Decision Letter 0

**DOI:** 10.1371/journal.ppat.1010375.r002

**Authors:** David Sacks

18 Jan 2022

Dear Dr. Spaeth,

Thank you very much for submitting your manuscript "Experimental evolution reveals post-transcriptional regulation as a novel driver of Leishmania fitness gain" for consideration at PLOS Pathogens. As with all papers reviewed by the journal, your manuscript was reviewed by members of the editorial board and by an independent reviewer.  Based on the reviews, we are likely to accept this manuscript for publication, providing that you modify the manuscript according to the review recommendations.

We found that some of the major conclusions/interpretations that dominate the Title and the abstract remain overly speculative (fine for the Discussion section). The ‘fitness-adapted ribosome’ model is especially speculative at this stage.

It seems that all of the (transcriptome, small RNA-ome, proteome) changes observed may be either directly or indirectly due to gene-dosage changes. The authors refer to “gene-dosage independent” changes in their abstract, and the title appears to reflect this interpretation. Are the authors interpreting potentially ‘indirect’ effects as ‘independent’ effects here?

As requested in the Review commons review, inclusion of an independent replicate of the experimental evolution data is important. For the transcriptomic analysis of the second RNAseq data set, were there also technical replicates for these samples, or were EP.8 / EP.9 and LP.9/LP.10 used as biologic replicates? In either case, a PCA plot would be helpful.

There appears to be no independent experimental replicate samples for the ncRNA analysis. Is this correct?

Please prepare and submit your revised manuscript within 30 days. If you anticipate any delay, please let us know the expected resubmission date by replying to this email.

When you are ready to resubmit, please upload the following:

[1] A letter containing a detailed list of your responses to all review comments, and a description of the changes you have made in the manuscript.

Please note while forming your response, if your article is accepted, you may have the opportunity to make the peer review history publicly available. The record will include editor decision letters (with reviews) and your responses to reviewer comments. If eligible, we will contact you to opt in or out

[2] Two versions of the revised manuscript: one with either highlights or tracked changes denoting where the text has been changed; the other a clean version (uploaded as the manuscript file).

Important additional instructions are given below your reviewer comments.

Thank you again for your submission to our journal. We hope that our editorial process has been constructive so far, and we welcome your feedback at any time. Please don't hesitate to contact us if you have any questions or comments.

Sincerely,

David Sacks

Section Editor

PLOS Pathogens

Kasturi Haldar

Editor-in-Chief

PLOS Pathogens

​orcid.org/0000-0001-5065-158X

Michael Malim

Editor-in-Chief

PLOS Pathogens

orcid.org/0000-0002-7699-2064

***********************

We found that some of the major conclusions/interpretations that dominate the Title and the abstract remain overly speculative (fine for the Discussion section). The ‘fitness-adapted ribosome’ model is especially speculative at this stage.

It seems that all of the (transcriptome, small RNA-ome, proteome) changes observed may be either directly or indirectly due to gene-dosage changes. The authors refer to “gene-dosage independent” changes in their abstract, and the title appears to reflect this interpretation. Are the authors interpreting potentially ‘indirect’ effects as ‘independent’ effects here?

As requested in the Review commons review, inclusion of an independent replicate of the experimental evolution data is important. For the transcriptomic analysis of the second RNAseq data set, were there also technical replicates for these samples, or were EP.8 / EP.9 and LP.9/LP.10 used as biologic replicates? In either case, a PCA plot would be helpful.

There appears to be no independent experimental replicate samples for the ncRNA analysis. Is this correct?

Reviewer Comments (if any, and for reference):

Figure Files:

While revising your submission, please upload your figure files to the Preflight Analysis and Conversion Engine (PACE) digital diagnostic tool, https://pacev2.apexcovantage.com. PACE helps ensure that figures meet PLOS requirements. To use PACE, you must first register as a user. Then, login and navigate to the UPLOAD tab, where you will find detailed instructions on how to use the tool. If you encounter any issues or have any questions when using PACE, please email us at figures@plos.org.

Data Requirements:

Please note that, as a condition of publication, PLOS' data policy requires that you make available all data used to draw the conclusions outlined in your manuscript. Data must be deposited in an appropriate repository, included within the body of the manuscript, or uploaded as supporting information. This includes all numerical values that were used to generate graphs, histograms etc.. For an example see here: http://www.plosbiology.org/article/info%3Adoi%2F10.1371%2Fjournal.pbio.1001908#s5.

Reproducibility:

To enhance the reproducibility of your results, we recommend that you deposit your laboratory protocols in protocols.io, where a protocol can be assigned its own identifier (DOI) such that it can be cited independently in the future. Additionally, PLOS ONE offers an option to publish peer-reviewed clinical study protocols. Read more information on sharing protocols at https://plos.org/protocols?utm_medium=editorial-email&utm_source=authorletters&utm_campaign=protocols

References:

Please review your reference list to ensure that it is complete and correct. If you have cited papers that have been retracted, please include the rationale for doing so in the manuscript text, or remove these references and replace them with relevant current references. Any changes to the reference list should be mentioned in the rebuttal letter that accompanies your revised manuscript. If you need to cite a retracted article, indicate the article’s retracted status in the References list and also include a citation and full reference for the retraction notice.

# Author comment: Author response to Decision Letter 0

**DOI:** 10.1371/journal.ppat.1010375.r003

10 Feb 2022

Attachment Submitted filename: Response letter PPATHOGENS-D-21-02620.docx Click here for additional data file.

# Editor report: Decision Letter 1

**DOI:** 10.1371/journal.ppat.1010375.r004

**Authors:** David Sacks

15 Feb 2022

Dear Dr. Spaeth,

We are pleased to inform you that your manuscript 'Experimental evolution links post-transcriptional regulation to Leishmania fitness gain' has been provisionally accepted for publication in PLOS Pathogens.

Before your manuscript can be formally accepted you will need to complete some formatting changes, which you will receive in a follow up email. A member of our team will be in touch with a set of requests.

Please note that your manuscript will not be scheduled for publication until you have made the required changes, so a swift response is appreciated.

IMPORTANT: The editorial review process is now complete. PLOS will only permit corrections to spelling, formatting or significant scientific errors from this point onwards. Requests for major changes, or any which affect the scientific understanding of your work, will cause delays to the publication date of your manuscript.

Should you, your institution's press office or the journal office choose to press release your paper, you will automatically be opted out of early publication. We ask that you notify us now if you or your institution is planning to press release the article. All press must be co-ordinated with PLOS.

Thank you again for supporting Open Access publishing; we are looking forward to publishing your work in PLOS Pathogens.

Best regards,

David Sacks

Section Editor

PLOS Pathogens

David Sacks

Section Editor

PLOS Pathogens

Kasturi Haldar

Editor-in-Chief

PLOS Pathogens

​orcid.org/0000-0001-5065-158X

Michael Malim

Editor-in-Chief

PLOS Pathogens

orcid.org/0000-0002-7699-2064

***********************************************************

Reviewer Comments (if any, and for reference):

# Editor report: Acceptance letter

**DOI:** 10.1371/journal.ppat.1010375.r005

**Authors:** David Sacks

11 Mar 2022

Dear Dr. Späth,

We are delighted to inform you that your manuscript, "Experimental evolution links post-transcriptional regulation to Leishmania fitness gain," has been formally accepted for publication in PLOS Pathogens.

We have now passed your article onto the PLOS Production Department who will complete the rest of the pre-publication process. All authors will receive a confirmation email upon publication.

The corresponding author will soon be receiving a typeset proof for review, to ensure errors have not been introduced during production. Please review the PDF proof of your manuscript carefully, as this is the last chance to correct any scientific or type-setting errors. Please note that major changes, or those which affect the scientific understanding of the work, will likely cause delays to the publication date of your manuscript. Note: Proofs for Front Matter articles (Pearls, Reviews, Opinions, etc...) are generated on a different schedule and may not be made available as quickly.

Soon after your final files are uploaded, the early version of your manuscript, if you opted to have an early version of your article, will be published online. The date of the early version will be your article's publication date. The final article will be published to the same URL, and all versions of the paper will be accessible to readers.

Thank you again for supporting open-access publishing; we are looking forward to publishing your work in PLOS Pathogens. 

Best regards,

Kasturi Haldar

Editor-in-Chief

PLOS Pathogens

​orcid.org/0000-0001-5065-158X

Michael Malim

Editor-in-Chief

PLOS Pathogens

orcid.org/0000-0002-7699-2064
